# Rejuvenation of Senescent Cells, In Vitro and In Vivo, by Low‐Frequency Ultrasound

**DOI:** 10.1111/acel.70008

**Published:** 2025-03-03

**Authors:** Sanjay K. Kureel, Rosario Maroto, Maisha Aniqua, Simon Powell, Ekta Singh, Felix Margadant, Brandon Blair, Blake B. Rasmussen, Michael P. Sheetz

**Affiliations:** ^1^ Biochemistry and Molecular Biology University of Texas Medical Branch Galveston Texas USA; ^2^ Visiting Scientist, Biochemistry and Molecular Biology University of Texas Medical Branch Galveston Texas USA; ^3^ Barshop Institute for Longevity and Aging Studies, Center for Metabolic Health University of Texas Health Science Center at San Antonio San Antonio Texas USA; ^4^ Department of Cellular and Integrative Physiology University of Texas Health Science Center at San Antonio San Antonio Texas USA

**Keywords:** aging, autophagy, calcium signaling, low frequency ultrasound, rejuvenation, senescence

## Abstract

The presence of senescent cells causes age‐related pathologies since their removal by genetic or pharmacological means, as well as possibly by exercise, improves outcomes in animal models. An alternative to depleting such cells would be to rejuvenate them to promote their return to a replicative state. Here we report that treatment of non‐growing senescent cells with low‐frequency ultrasound (LFU) rejuvenates the cells for growth. Notably, there are 15 characteristics of senescent cells that are reversed by LFU, including senescence‐associated secretory phenotype (SASP) plus decreased cell and organelle motility. There is also inhibition of β‐galactosidase, p21, and p16 expression, telomere length is increased, while nuclear 5mC, H3K9me3, γH2AX, nuclear p53, ROS, and mitoSox levels are all restored to normal levels. Mechanistically, LFU causes Ca^2+^ entry and increased actin dynamics that precede dramatic increases in autophagy and an inhibition of mTORC1 signaling plus movement of Sirtuin1 from the nucleus to the cytoplasm. Repeated LFU treatments enable the expansion of primary cells and stem cells beyond normal replicative limits without altering phenotype. The rejuvenation process is enhanced by co‐treatment with cytochalasin D, rapamycin, or Rho kinase inhibition but is inhibited by blocking Sirtuin1 or Piezo1 activity. Optimized LFU treatment parameters increased mouse lifespan and healthspan. These results indicate that mechanically induced pressure waves alone can reverse senescence and aging effects at the cellular and organismal level, providing a non‐pharmacological way to treat the effects of aging.

## Graphical abstract

Optimized low‐frequency ultrasound rejuvenates induced senescent cells and aged mice. Our results show that LFU reverses 15 aspects of senescent cells including growth and revitalizes aged mice.

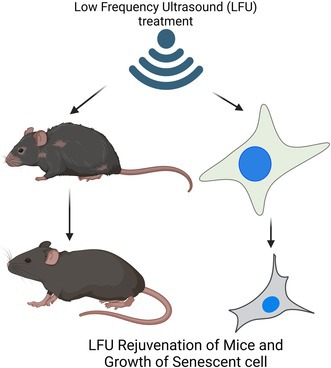

## 1 Introduction

Cell senescence is one of the hallmarks of the aging process that is purportedly defined by a permanent block to cell growth (Hernandez‐Segura et al. 2018; Lopez‐Otin et al. 2013). It was first noted in 1961 by Hayflick and Moorhead that primary cells in culture stopped growing after a certain number of divisions; that is, they became senescent (Hayflick and Moorhead 1961). Transplantation of senescent cells into young mice caused physical deterioration and age‐related pathologies (Xu et al. 2017), whereas depletion of these cells in aged mice genetically or with senolytics slowed the development of age‐related pathologies and enhanced lifespan (Bussian et al. 2018; Cai et al. 2020; Caland et al. 2019; Chae et al. 2021; Chang et al. 2016; Jeon et al. 2017; Patil et al. 2019; Peilin et al. 2019; Xu et al. 2018). These results indicated that cellular senescence was a major driver of the aging process and removing senescent cells was critical for improving performance in aged organisms. Because senescence was believed to be a state of permanent cell cycle arrest in which cells were still metabolically active (Childs et al. 2015), the selective lysis of senescent cells was considered the best way to remove their negative effects (Chen et al. 2021; Kirkland and Tchkonia 2020; Kirkland et al. 2017; Lorenzo et al. 2023; Yamaura et al. 2023).

While cell senescence is typically considered detrimental, it should be noted that senescence has general physiological significance in preventing the propagation of damaged cells, suppressing tumor progression, in early development (Storer et al. 2013), wound healing (Demaria et al. 2014) and in tissue repair processes (Munoz‐Espin and Serrano 2014). Despite the benefits of senescent cells, they are linked to many age‐associated maladies (Borghesan et al. 2020; Childs et al. 2017; McHugh and Gil 2018), including in the lung, adipose tissue, aorta, pancreas, and osteoarthritic joints (Adams 2009; He and Sharpless 2017; Herranz and Gil 2018). Thus, there are potentially many benefits from decreasing the fraction of senescent cells in tissues during aging. Senescent cells secrete pro‐inflammatory molecules, growth factors, chemokines, extracellular matrices, proteases, and cytokines, collectively known as the senescence‐associated secretory phenotype (SASP) (Tchkonia et al. 2013; Young and Narita 2009). An increase in the level of SASP catalyzes many age‐related problems (Coppe et al. 2008; da Silva et al. 2019). Therefore, targeted elimination of senescent cells by senolytics may improve age‐associated pathologies, including osteoarthritis (Peilin et al. 2019), diabetes (Thompson et al. 2019), osteoporosis, neurodegenerative diseases (Penney and Tsai 2018) and overall lifespan (Xu et al. 2018). Senolytics are considered a way to diminish specific effects of aging in tissues (Yousefzadeh et al. 2018; Zhu et al. 2015), and they have entered clinical trials. However, there is currently no approved senolytic‐based treatment for humans (Cai et al. 2020; Libertini et al. 2018; Neves et al. 2017).

Autophagy inhibition and mitochondrial dysfunction are hallmarks of aging and cellular senescence (Hernandez‐Segura et al. 2018; Kaushik et al. 2021). Dynamic changes in mitochondrial fusion and fission are essential for healthy mitochondrial function, and increased fusion contributes to senescence (Bartolak‐Suki et al. 2017). There is evidence that exercise decreases senescence (Englund et al. 2021) possibly due to increased mitochondrial fission (Bartolak‐Suki et al. 2017; Helle et al. 2017). Although changes in lysosomal and mitochondrial functions may be linked to each other, it appears that inhibition of lysosomal autophagy is a driver of aging (Carosi et al. 2022; Cassidy and Narita 2020; Rubinsztein et al. 2011).

Mutations in genomes can increase lifespan in worms and flies, and they are largely linked to metabolism and proteostasis pathways that inhibit the onset of senescence but do not reverse senescence (Zhang et al. 2022). Strategies to prolong lifespan, including the use of small molecules (e.g., rapamycin and metformin) and caloric restriction, often activate autophagy.

An alternative to senolysis is to block the transition to the senescent state, but such interventions need to be relatively early in the aging process (Kvell and Pongracz 2012). Many studies have shown that senescence markers can be reversed using various strategies, such as vitamin E treatment (Ezquer et al. 2014), PI3K inhibitor (Le et al. 2022), inhibition of cyclin‐dependent kinases (CKIs) (Jeanblanc et al. 2012), protease inhibitor (PIs) (Lefevre et al. 2010), PARTP‐1 inactivation, proteasomal degradation (Selle et al. 2007) and inactivation of FOXO4 by FOXO4‐DRI peptide (Krimpenfort and Berns 2017) cause some degree of marker reversal but not growth of rigorously defined senescent cells. Extracellular vesicles derived from the young MSCs reduced the senescence markers of cocultured aged epithelial cells in vitro and promote angiogenesis and vascular repair (Wang et al. 2020) in vivo. In a recent study by Li et al. 2021, they showed that transient electric current reduces the senescence markers of BMSCs derived from old patients. However, these studies do not show growth of truly senescent cells as we found with low‐frequency ultrasound (LFU).

Even so, physical exercise is accepted as an early intervention that slows aging (Chubanava and Treebak 2023; De Sousa Lages et al. 2022; Galkin et al. 2023) and is possibly a senolytic (Chen et al. 2021). Although the effects of exercise could be purely physical, exercise affects the brain and other organs through increased secretion of myokines, such as irisin (Ning et al. 2022).

Ultrasound creates pressure waves in cells that cause mechanical stresses. These effects are safe for normal tissues and functions (Ahmadi et al. 2012). Other studies use low‐intensity pulsed ultrasound for healing (Leighton et al. 2017) and for transiently opening the blood‐brain barrier (Balbi et al. 2022). Previously, we found that ultrasound could activate apoptosis selectively in tumor cells (Singh et al. 2021); and it was logical to test if it had similar effects on senescent cells. When ultrasound was applied to senescent cells, LFU treatment restored the growth of chemically induced and replicatively senescent cells. Mechanistically, LFU activated autophagy and Piezo1 channels, while inhibited mTORC1, SASP secretion, β‐galactosidase expression, and decreased cell size plus mitochondrial length. Notably, normal cells treated with LFU, secreted factor(s) that activated the growth of senescent cells. Thus, purely mechanical stimuli can selectively rejuvenate senescent cells.

## 2 Results

### 2.1 Development and Optimization of LFU

Since previous studies showed that LFU caused apoptosis of cancer cells (Singh et al. 2021; Yao et al. 2022), we treated senescent Vero cells with an LFU apparatus (Figure S1A,B) to determine if they behaved similarly. Surprisingly, the senescent cells grew and increased in number after LFU treatment with no apoptosis (Figure S1C–F). In the LFU bath, cells were positioned 7–10 cm above the ultrasound transducer in the far field of the ultrasound (Figure S1A,B). After testing different ultrasound frequencies and power levels for inducing cell growth, the greatest growth was with LFU of 32.2 kHz, not 39 kHz, and with a power of 4 kPa as measured by a calibrated hydrophone (Figure S1C,D). Similarly, the duration of LFU and the duty cycles (on/off times) affected the growth rate (Figure S1E,F). An LFU of 32.2 kHz at 4 kPa for 30 min with a duty cycle of 1.5 s on and 1.5 s off was optimal for inducing the growth of the senescent cells; thus, these values were used for subsequent cell and mouse experiments.

**VIDEO 1:** This video illustrates that bleomycin sulphate (BS) treated HFF senescent cells are truly senescence. Time‐lapse video of BS treated cells after 22 days of incubation shows no cell division which confirms that these cells are truly senescent. Images were taken every 30 min for 24 h. Video content can be viewed at https://onlinelibrary.wiley.com/doi/10.1111/acel.70008

**VIDEO 2:** This time‐lapse video illustrates cells previously treated with BS and incubated for 22 days, now treated with LFU for 30 min. Images were captured every 30 min for 24 h. We can see the three of the original senescent cells undergo cell division showing that LFU activates the growth of senescent cells. Video content can be viewed at https://onlinelibrary.wiley.com/doi/10.1111/acel.70008

**VIDEO 3:** This time lapse video illustrates the zoomed view, showing LFU treatment rejuvenates the BS‐treated senescent cells. Video content can be viewed at https://onlinelibrary.wiley.com/doi/10.1111/acel.70008

**VIDEO 4:** This is the time lapse video of senescent cells videoed for 48 h before LFU treatment. These cells were incubated for 4 days after inducing senescence. Video content can be viewed at https://onlinelibrary.wiley.com/doi/10.1111/acel.70008

**VIDEO 5:** This is the time lapse video of 30 min LFU treated cells. These cells are from Video 4. Images were captured every 30 min for 48 h after LFU treatment. Video content can be viewed at https://onlinelibrary.wiley.com/doi/10.1111/acel.70008

To test the generalizability of LFU treatments for reversing cell senescence, was induced by doxorubicin (Dox), hydrogen peroxide (H_2_O_2_), sodium butyrate (SB) or bleomycin sulfate (BS) for 36–48 h, followed by 4 days of incubation in medium (Alessio et al. 2021). Senescence was characterized by a block of cell growth (statistically, not different from zero growth), high β‐galactosidase activity (> 90% of cells were positive), a larger cell size, and production of SASP that inhibited the growth of normal cells (Herranz and Gil 2018; Young and Narita 2009) All four criteria were met with cells treated by each type of drug (Figure S2A–G) (Alessio et al. 2021). Thus, by all criteria, the 4 drugs were able to induce senescence in Vero cells.

### 2.2 Senescent Cells Are Rejuvenated for Growth by LFU

**FIGURE 1:**
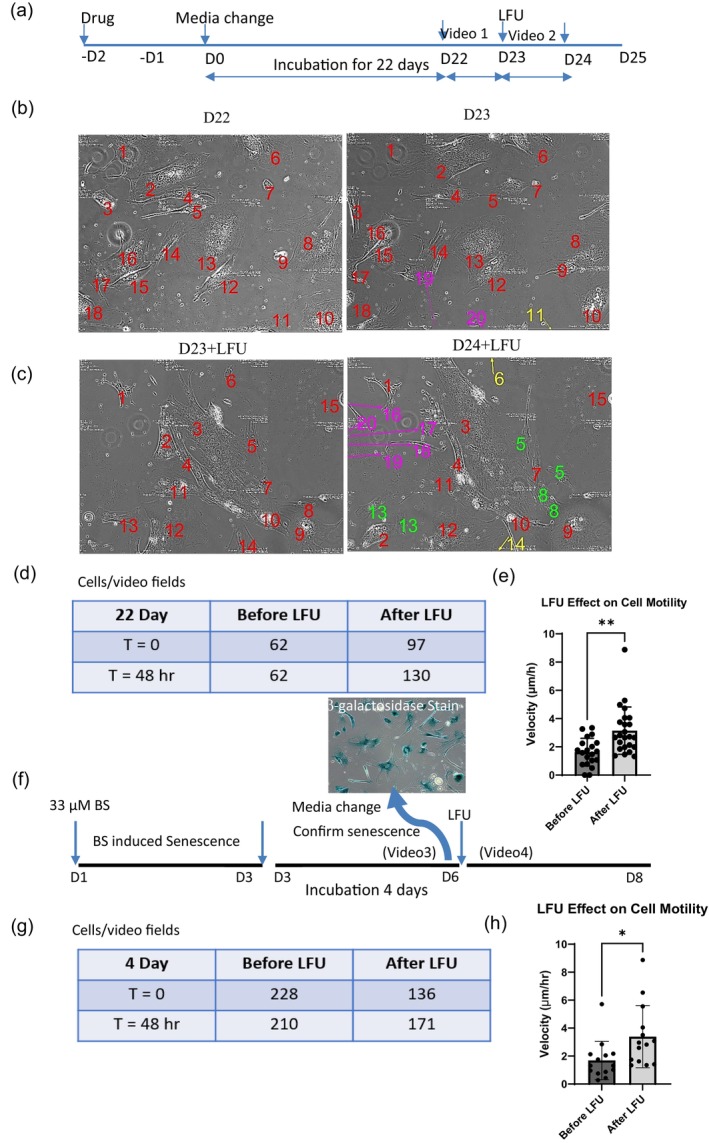
Fully senescent cells are rejuvenated for growth and motility by LFU. (a) The diagram shows the protocol used to produce fully senescent cells. Note that the timing of the Videos 1, 2, 3 are 48 h before and 48 h after LFU treatment for 30′, respectively. (b) Time‐lapse video images of a field with 18 Bleomycin sulfate (BS) treated cells incubated for 22 days at the beginning and the end of a 24 h observation period (red numbers). Images were taken every 30′. Two cells that entered the field during that period are marked with purple numbers and one that left the field with yellow. (c) Time‐lapse images of a field of 14 cells after a 30' LFU treatment at the beginning and the end of a 24 h observation period (red numbers). Three of the original cells divided (#s 5, 8 and 13) and the daughter cells are noted by green numbers. Five cells entered the field (purple numbers) and two left the field (yellow numbers). (d) Twenty‐eight image fields were tracked for 48 h before and for 48 h after LFU treatment and the number of cells in those fields were counted. (e) Cell velocity (determined by displacements of nuclei over each hour for 24 h). (f) Diagram of 4 days incubation of BS stressed cells with an image of β‐galactosidase staining of the cells after 4 days of incubation. Note the timing of the Videos 4 and 5 are for 48 h before and after LFU treatment. (g) Twenty‐eight image fields were tracked for 48 h before and after LFU treatment and the number of cells in the fields were counted. (h) The velocity of the cell movements was determined by measuring the displacements of the nuclei over each hour of imaging for 24 h. Results are shown as mean ± SD, at least 108 cells were analyzed, *n* > 3 experiments, and significance was determined using two‐tailed unpaired *t*‐test. *p < 0.05, **p < 0.002.

It was possible that many drug‐treated cells were quiescent and only a fraction was senescent. However, quiescent cells lacked β‐galactosidase activity, whereas over 95% of drug‐treated cells stained for β‐galactosidase activity after 4‐day incubation in media (Figure S2F,G) (Alessio et al. 2021). Thus, it seemed that the fraction of quiescent cells was very low in these preparations. To further test if cells were senescent and if the senescent cells were reactivatable, human foreskin fibroblast (HFF) cells were analyzed by time‐lapse video microscopy. In particular, BS‐treated HFF cells were incubated for 22 days in media after a 2‐day drug treatment to assure senescence (Figure 1a). Time‐lapse videotaping of many cells for 48 h determined that senescent cells did not divide (Figure 1b,d) and motility was limited (Figure 1e). After LFU treatment for 30′, however, cells grew (Figure 1c,d) and motility increased (Figure 1e). The velocity of senescent cell nucleus migration was 1.6 μm/h, but after LFU treatment, the velocity of the same cells increased nearly two‐fold to 3.2 μm/h (Figure 1e). Thus, fully senescent cells were activated for growth and motility by LFU without senolysis.

**FIGURE 2:**
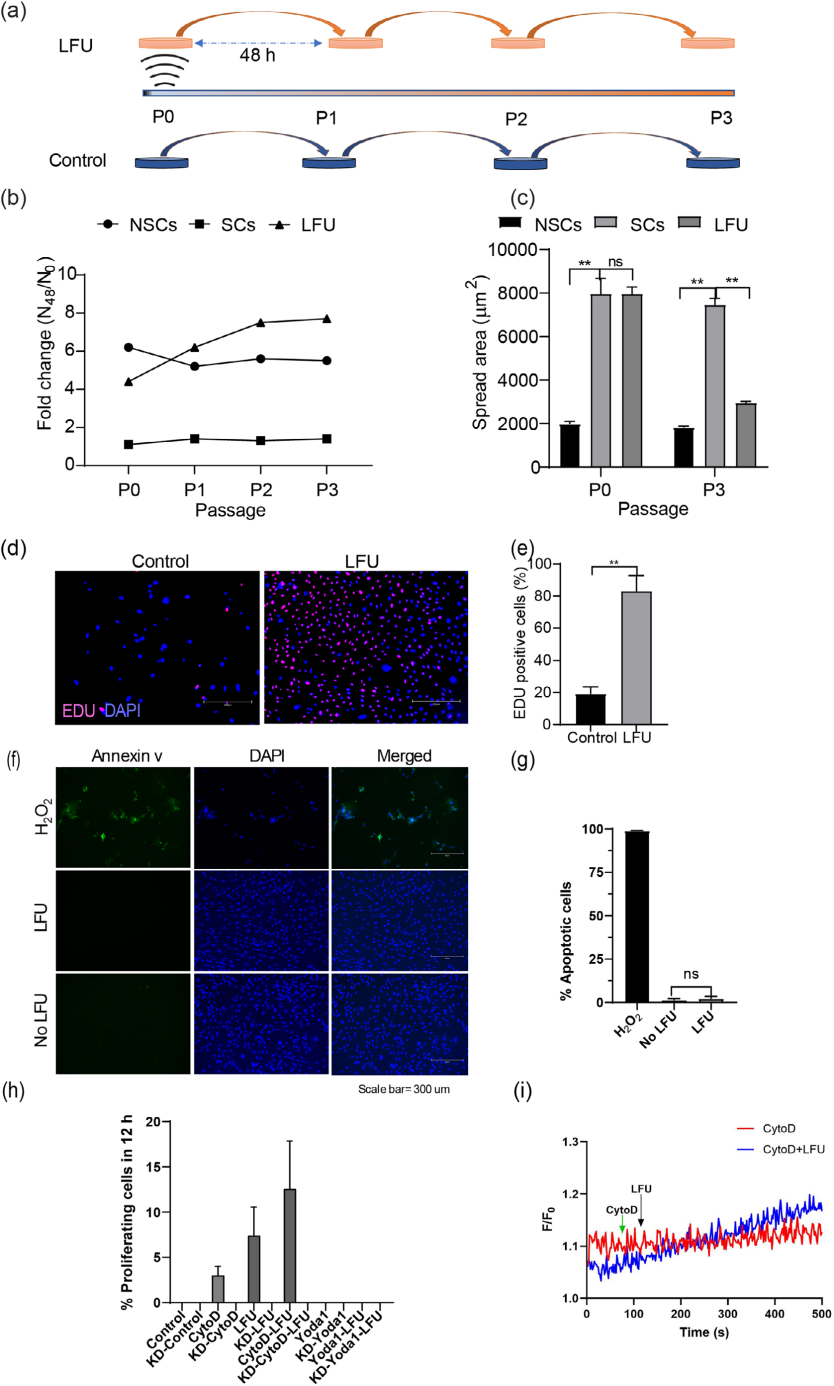
Low frequency ultrasound (LFU) reverses cell senescence. (a) Schematic illustration showing experimental design of LFU‐induced reversal of senescence after Sodium Butyrate (SB) treatment for 48 h followed by incubation for 4 days. Cells were treated with LFU for 30′ and then passaged every 2 days. (b) Growth of SB treated senescent Vero cells after LFU treatment and passaged every 48 h for 8–10 days. NSCs are the non‐senescent Vero cells. Graph shows growth of normal Vero cells (NSC), BS senescent (SC) and LFU‐treated (LFU) senescent cells as fold change in cell number over 48 h for passages from P0 to P3 every 48 h. (c) Cell area of LFU‐treated senescent cells (LFU) is largely restored to normal by P3. (d) Representative EDU‐stained images of senescent P3 control and LFU treated senescent P3 cells. Scale bar = 300 μm. (e) Quantification of EDU‐stained senescent and LFU treated BS senescent P3 cells shown as mean ± SD, for > 200 cells in each condition. (f) Annexin V staining for apoptosis of H_2_O_2_ (killed with 200 mM of H_2_O_2_) or of senescent (made senescent with 200 μM of H_2_O_2_) cells treated w/wo LFU after 48 h. (g) Percentage of viable cells after 200 mM of H_2_O_2_ or senescent cells (Doxorubicin 500 nM, and H_2_O_2_ 200 μM) treated with LFU. Results are plotted as mean of three replicates and ± SD. At least 35–50 random cells were analyzed from each of the three replicates. Non‐parametric Mann Whitney test was used to determine the statistical difference between the two groups. (h) Proliferation of BS senescent cells with piezo1 or after siRNA knockdown of piezo1 (noted by KD) was determined from 12 h timelapse videos of the cells. The percentage of dividing cells was calculated by dividing the number of cell divisions by the total number of cells captured from 25 to 35 fields of view (> 200 cells). The drug concentrations were 25 nM of cytochalasin D and 10 μM of Yoda 1. Results are plotted as the mean of three independent experiments ± SD. (i) 25 nM cytochalasin D (black arrow). Administration of 25 nM of cytochalasin D (green arrow) does not change the basal Ca^2+^ level in senescent cells (red trace). However, when senescent cells were pre‐treated with 25 nM of cytochalasin D for 10 min and LFU was applied (black arrow), there was a slow increase in the basal Ca^2+^ level (blue trace). All graphs were plotted by mean ± SD and *p* values: Ns *p* > 0.05, *p < 0.05, **p < 0.002. Minimum 200 cells were analyzed from three independent experiments.

As a further test of rejuvenation, BS‐treated cells were incubated in media for 4 days after a 2‐day drug treatment (Figure 2), (a time period that was sufficient for treated cells to move to a senescent state (Chitaley and Webb 2001)). After a 4‐day incubation protocol, all BS‐treated cells expressed β‐galactosidase, and time‐lapse videos showed fewer cells after 48 h, indicating that some drug‐treated cells apoptosed (Figure 1g). After LFU, drug‐treated cells grew, and the growth rate was a similar to that of the 22‐day incubated cells. Also, cell motility increased after LFU compared to before (Figure 1h).

### 2.3 LFU Causes Return to Normal Size and High Growth Rates Without Senolysis

To determine if LFU caused sustained cell growth and near normal behavior, the LFU rejuvenated SB Vero cells were followed for several passages (Figure 2a). After only 30 min of LFU, Vero cells grew until at least passage 3 without a significant loss in growth rate as determined by cell counting (Figure 2b). By the second passage, the growth rate exceeded the parental rate, non‐senescent line (Figure 2b). In addition, rejuvenated cells became smaller with growth, reaching their normal cell size by the third passage (Figure 2c). Another measure of growth was an EdU incorporation assay, and a large fraction of cells incorporated EdU into their DNA (Figure 2d,e). A concern was that the LFU treatment might cause some apoptosis. As a positive control, H_2_O_2_ senescent cells (200 μM) were treated with a lethal dose of H_2_O_2_ (200 mM) that resulted in high levels of annexin V staining (Figure 2f,g). When H_2_O_2_ senescent cells were analyzed before and after LFU treatment, there were only background levels of staining, indicating that LFU did not cause senolysis (Figure 2f,g). Thus, it appeared that LFU treatment activated the growth of senescent cells without apoptosis.

To determine if all forms of senescent cells could be rejuvenated, we treated the other three types of chemically induced senescent cells (hydrogen peroxide, bleomycin sulfate and doxorubicin) with LFU. In all cases, LFU caused a significant increase in the growth rate of senescent cells (Figure S3A–C). In the first 2 days after LFU treatment, large cells divided, and the size distribution shifted to smaller sizes (Figure S3D–F).

### 2.4 LFU Activates the Mechanosensitive Ion Channel Piezo1, Needed for Growth, but Actin Dynamics Are Important

The first component of a cell that encounters LFU is the plasma membrane. In the case of tumor cell treatment with LFU, piezo1 ion channels were activated and contributed to tumor cell apoptosis (Singh et al. 2021). Further, piezo1 regulated Ca^**2+**^ flux and Ca^**2+**^ signaling affected autophagy (Sukumaran et al. 2021; Velasco‐Estevez et al. 2020). Thus, if LFU stimulated growth by activating autophagy downstream of Ca^**2+**^ entry, then Ca^**2+**^ loading should have followed LFU treatment. Within 1–2 min of starting LFU, cytoplasmic Ca^**2+**^ levels often spiked for about a minute (Figure S4A,B) but the timing and frequency of spikes varied stochastically, giving on average about 2 spikes/10 min. Inhibition of Ca^**2+**^ channels with Rutheneum Red, an inhibitor of many mechanosensitive channels, including piezo1 (Gnanasambandam et al. 2017) or GsMTX4 blocked Ca^**2+**^ spiking (Figure S4A). To know if channels were involved in LFU‐induced growth, EdU incorporation into the senescent cell DNA was measured after LFU treatment with or without Rutheneum Red, which significantly inhibited growth (Figure S4C,D). To test replicatively senescent cells, HFF cells passage 21 (P21) were LFU treated with/without GsMTx4 and assayed for growth by EdU. LFU significantly increased the growth of the P21 HFFs, but that effect was inhibited by GsMTx4 (Figure S4E); although GsMTx4 did cause a modest increase in growth, perhaps by altering Ca^2+^ homeostasis. In separate experiments, knockdown of piezo1 by siRNA also inhibited growth, indicating that piezo1 was important (Figure S4F–I and Figure 2h). Thus, it appeared that LFU stimulation of growth relied upon the mechanosensitive ion channel, piezo1.

The increased motility of senescent cells after LFU indicated that actin dynamics were increased by LFU. Further, there were earlier reports that the growth of aged immune cells was increased by adding an inhibitor of actin polymerization (Brock and Chrest 1993). To test the possibility that an inhibitor of actin polymerization could aid rejuvenation of senescent cells, we added cytochalasin D to senescent cells. High concentrations of cytochalasin D used to block actin polymerization caused cell rounding as expected, but low concentrations that allowed actin polymerization stimulated growth. Low cytochalasin D concentrations, along with LFU, caused a major stimulation of growth consistent with the hypothesis that the reversal of senescence involved activation of actin dynamics (Figure 2h). When Ca^2+^ levels were measured in the cells treated with cytochalasin D and LFU, the Ca^2+^ spikes were blocked, but Ca^2+^ levels slowly increased (Figure 2i). Thus, it seemed that increased Ca^2+^ levels and actin dynamics were important for the reversal of senescence.

### 2.5 LFU Activates Mitochondrial Dynamics and Motility Along With Lysosomes

**FIGURE 3:**
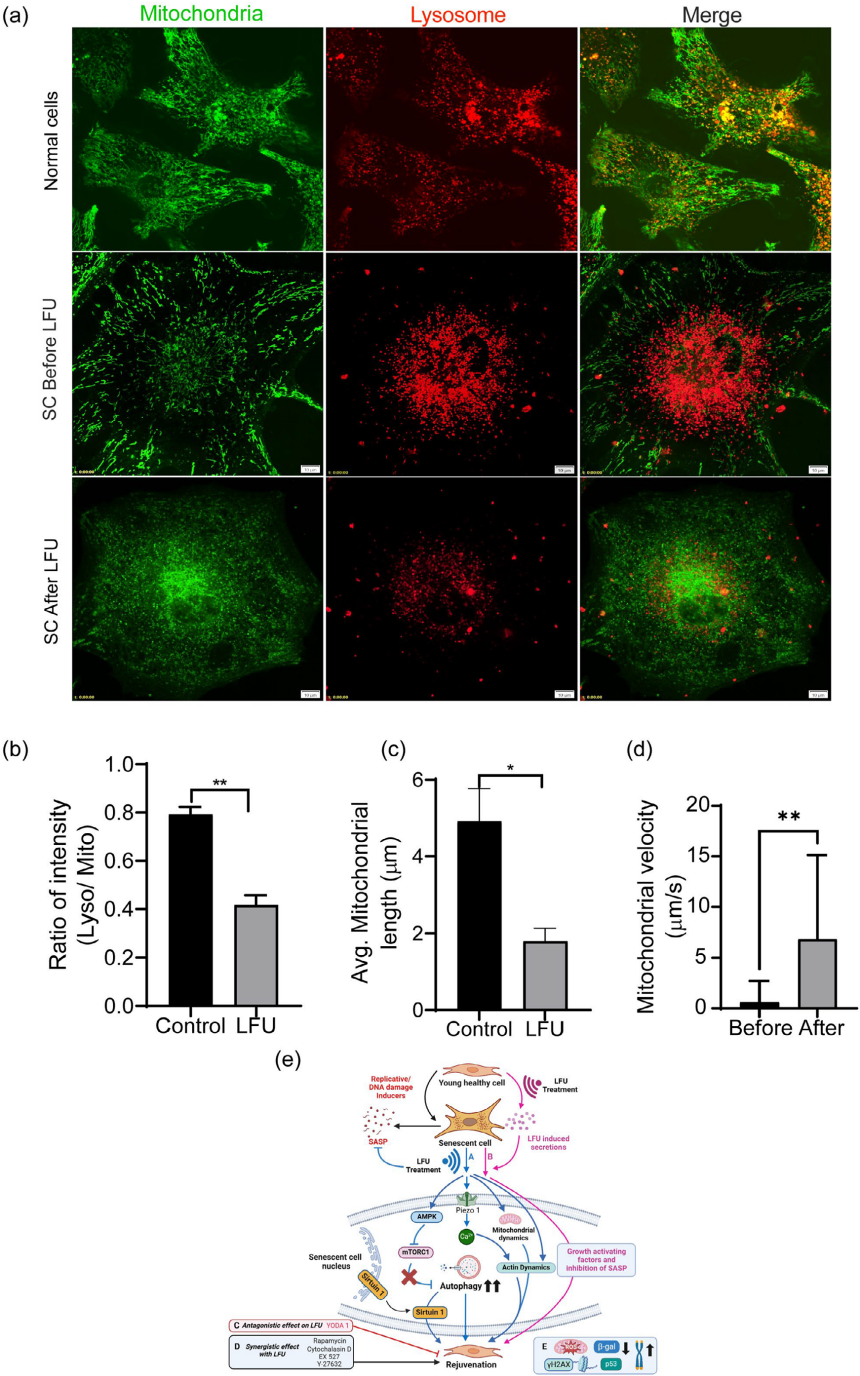
Low frequency ultrasound decreases mitochondrial length and lysosome staining intensity in senescent cells. (a) Representative immunofluorescent images of mitochondria and lysosome morphology in normal, senescent (BS–treated for 30 h and incubated for 3 days) and LFU–treated senescent Vero cells using Mitotracker and Lysosome tracker. Scale bar = 10 μm. (b) Ratio of total cell intensities of lysosomal to mitochondrial staining is decreased by LFU treatment of senescent cells. (c) Quantification of mitochondrial length shows decreased length after LFU treatment. Results are shown as mean ± SD, minimally 108 cells were analyzed, *n* > 3 experiments, and significance was determined using two‐tailed unpaired *t*‐test. *p < 0.05, **p < 0.002. (d) Velocity of mitochondria before and after LFU treatment. Images were capture every 5 s for 10 min. A minimum of 10–12 mitochondrial puncta were manually tracked using ImageJ software. (e) Illustration of a working model for the rejuvenation of senescent cells by LFU depicting the different pathways involved. The two main routes ‘A and B’ are represented by the blue and pink arrows, respectively. A involves the direct stimulation of senescent cells by LFU treatment. B implies a paracrine mode of rejuvenation achieved via secretory factors produced by treating normal cells with LFU. ‘C’ Inhibitors that show an antagonistic effect towards LFU treatment. ‘D’ Inhibitors and drugs that show a synergistic effect with LFU and thereby enhance rejuvenation. ‘E’ Various hallmarks of senescent cells that can be modulated by LFU treatment in the rejuvenated cells.

To determine if organelle motility was activated, we followed the movements of mitochondria. The mitochondria of senescent cells were often longer than in normal, growing cells (Figure 3a). LFU treatment caused fragmentation of mitochondria and a decrease in the lysosome staining intensity (Figure 3b,c). To determine movement velocities, the positions of Mitotracker‐labeled mitochondria were recorded every 5–10s. After LFU treatment, mitochondria were significantly smaller (Figure 3a,c) and linear velocities were significantly greater (by over 5‐fold) than before (Figure 3d). In addition, there were many mitochondrial fission and fusion events after LFU (data not shown). For consistency, only the velocities of mitochondria that did not undergo fission or fusion during the observation period were tracked. Lysosomes in the same cells decreased in staining intensity and moved from the center to the periphery (Figure 3a) supporting the hypothesis that LFU caused rejuvenation by activating autophagy through phagosome fusion with lysosomes as part of a larger scheme of rejuvenation (Figure 3e). Thus, LFU treatment activated whole‐cell, lysosomal, and mitochondrial dynamics, indicating that both actin‐ and microtubule‐based motility were promoted by LFU.

### 2.6 LFU Inhibits SASP Secretion

To determine if LFU treatment of SASP‐secreting BS‐induced senescent cells blocked further secretion of SASP, 24 h supernatants were collected before and after LFU treatment (Figure S5A). The supernatant from before LFU treatment (S0) inhibited the growth of and increased the size of normal HFF cells whereas the 24 h supernatant after LFU (S24) supported normal growth without altering size (Figure S5B–D). In a separate experiment where the levels of SASP components in supernatants from replicatively senescent HFFs were measured before and after LFU treatment, the levels of eight SASP components were lower after LFU exposure, including interleukins (IL‐6, IL‐8, IL‐10 and IL‐15), as well as the pro‐inflammatory molecules (TNF‐α, IFN‐γ, VEGF and MIP‐1a) (Figure S5E). Thus, LFU‐mediated rejuvenation of senescent cells blocked the secretion of SASP components.

### 2.7 LFU of Normal Cells Causes the Secretion of Components That Activate Senescent Cell Growth

**FIGURE 4:**
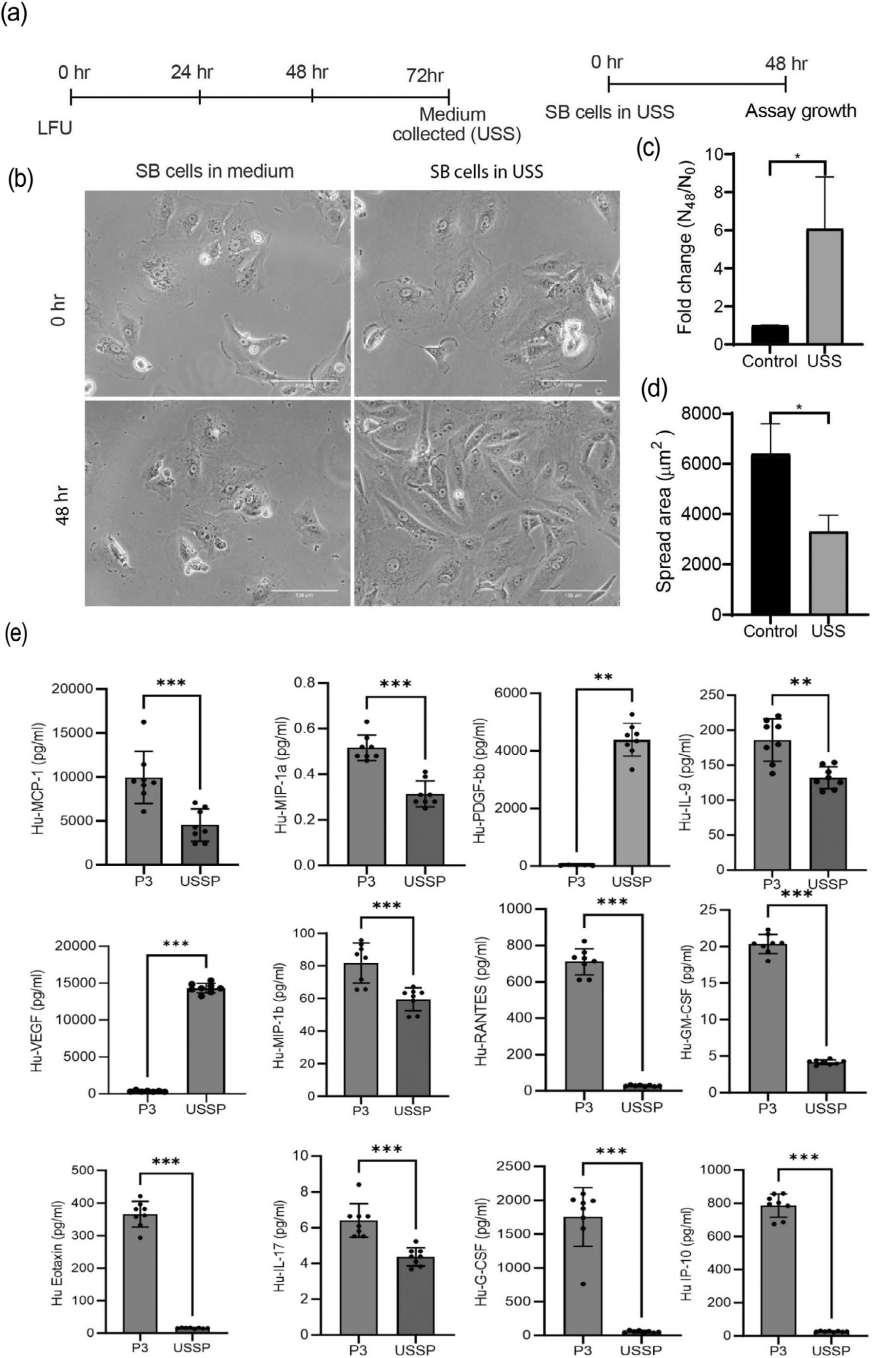
Normal cells treated with LFU secrete growth activating factors. (a) Timeline and strategy of LFU treatment in normal proliferating cells (passage 3 HFF cells). schematic showing normal cells that were treated with LFU four times in the same media. The supernatant was then collected (USS) for incubation with senescent cells for 48 h. (b) Brightfield images show changes in the morphology of senescent cells cultured in the supernatant collected from LFU‐treated normal cells and after 48 h. Senescent cells in normal growth medium were used as controls. (c) The graph shows that the growth of SCs in USS increased; (d) whereas the spread area of senescent cells decreased with USS. (e) Chemokines and cytokines in supernatants collected from untreated and LFU‐treated cells (P3 HFF) were analyzed using a Multiplex immunoassay. Results are plotted as mean ± SD, *n* = 6 replicates. Mann–Whitney Test was used to determine the statistical significance, ns = not significant, *p < 0.05, **p < 0.01, ***p < 0.0001, and ****p < 0.00001. Graphs were plotted as mean ± SD; *p < 0.05, **p < 0.002, ***p < 0.0001. At least 200 cells were analyzed from three independent experiments for graphs C and D. Scale bar = 300 μm.

Many studies have shown that physical exercise delays peripheral tissue (Ning et al. 2022) and brain (Penney and Tsai 2018) aging. To test the possibility that LFU could benefit senescent cells through an indirect effect on normal cells, that is, the production of a pro‐rejuvenation factor(s), we treated normal HFF cells with LFU for 3 days (1 h/day) and collected the supernatant after ultrasound treatment (USS) (Figure 4a). When BS‐induced senescent HFF cells were treated with USS, the senescent cells grew (Figure 4a–c) and decreased their spread area (Figure 4d). The levels of chemokines and cytokines in the supernatants from the third passage (P3) of treated HFFs after 3 days (USSP) differed from the levels in the supernatant of P3 non‐treated HFFs (USS) (Figure 4e). Levels of IL‐9, IL‐17, MCP‐1, MIP‐1a, MIP‐1b, RANTES, GM‐CSF, EOTAXIN, G‐CSF, and IP‐10 were lower, while levels of PDGF‐bb and VEGF were higher in USSP versus the control supernatants (Figure 4e). Thus, LFU treatment of normal cells stimulated the secretion of factors that activated the growth of senescent cells.

### 2.8 LFU Activates Autophagy

To determine if LFU activated autophagy in senescent cells, a GFP‐LC3‐RFP construct was expressed in cells that were made senescent. Autophagy levels correlated with a decrease in the GFP‐to‐RFP fluorescence ratio, indicating that GFP was in an acidic compartment that decreased GFP fluorescence. When senescent cells were treated with LFU, the GFP:RFP ratio dropped (Figure S11A,B), indicating that GFP‐LC3‐RFP entered an acidic lysosomal compartment that decreased GFP fluorescence. When chloroquine diphosphate (CCD) was added, LFU‐activated autophagy was inhibited (Figure S11B).

### 2.9 Inhibition of Sirtuin1 Blocks LFU‐Induced Rejuvenation

Another protein linked to senescence was Sirtuin1 (SIRT1), an analog of the yeast deacetylase ser1 that inhibited senescence (Liu et al. 2018). After LFU treatment, SIRT1 moved from the nucleus to the cytoplasm and its level of staining decreased (Figure S6A,F). Inhibition of SIRT1 with EX‐527 during LFU treatment blocked rejuvenation and decreased cytoplasmic SIRT1 levels (Figure S6C–F). Further SIRT1 inhibition decreased autophagy activity. The SIRT1 inhibitor, EX‐527, either alone or with LFU, caused a significant increase in the GFP:RFP ratio of the GFP‐LC3‐RFP construct relative to LFU treatment alone, indicating that autophagy was inhibited (Figure S6B). Thus, SIRT1 activity, a NAD^+^–dependent deacetylase, was needed for the strongest activation of autophagy after LFU treatment.

Since GFP fluorescence of GFP‐LC3‐RFP decreased in the presence or absence of CCD after LFU (Figure S6B), LFU increased autophagy possibly by inhibition of mTORC1. Rapamycin activated autophagy by inhibiting mTORC1 (Carosi et al. 2022) and acted synergistically with LFU to increase growth (Figure S6G) indicating that LFU inhibition of mTORC1 was increased by rapamycin (Figure S6G). Thus, LFU pressure oscillations reversed the senescence‐related decrease in autophagy and increase in mTOR activity.

### 2.10 Markers of Senescence Are Restored to Normal by LFU Treatment of Senescent Cells

To further explore the effect of LFU on senescence, diagnostic markers of senescent cells were examined. Notably, BS‐induced senescent Vero cells had high nuclear levels of p53 staining, γH2AX foci, H3K9me3, ROS, and MitoSOX that were all decreased by LFU treatment (Figure S7A–H). Thus, by many criteria, LFU treatment caused a dramatic reversal of the senescent state.

Other factors linked to cell senescence included telomere shortening (Nadri et al. 2022), DNA hypomethylation (Gentilini et al. 2013) and increased H3K9me3. After LFU, there was a striking increase in telomere length of replicatively senescent HFF cells (Figure S7I) and a small increase in replicatively senescent mesenchymal stem cells (MSCs) (Figure S7J). Further, the low DNA methylation levels of replicatively senescent HFF cells increased with LFU (Figure S7K,L). Finally, levels of H3K9me3 decreased after LFU (Figure S7D,E). Thus, LFU treatment of senescent cells reversed senescence‐dependent changes in the nucleus, consistent with restoration of the normal cell state by LFU.

### 2.11 RNA‐Seq Analysis Reveals Major Transcriptional Changes After LFU

RNA‐seq analysis of LFU‐treated senescent cells showed that the expression levels of 50 genes were upregulated by a factor of two or more upon rejuvenation, whereas 140 genes were downregulated. Of particular interest were SASP proteins that were downregulated upon rejuvenation, which included IGF2, IGFBP2, FGF7, and C1Q‐TNF7. Pathway analysis found that many of the genes activated by LFU were in pathways induced by viral infections (Tables S1 and S2). This was consistent with the activation of growth upon rejuvenation.

### 2.12 Rejuvenation Is Increased by Rho Kinase Inhibition

Previous studies from our laboratory showed that a different ultrasound treatment caused apoptosis of tumor cells but not of normal cells (Singh et al. 2021; Yao et al. 2022). In that case, LFU‐dependent tumor cell apoptosis increased after microtubule depolymerization by nocodazole, and the Rho kinase inhibitor (Y‐27632) blocked LFU‐activated apoptosis in correlation with myosin contractility (Chitaley and Webb 2001). In contrast, senescent HFF cells had greater LFU rejuvenation with Y‐27632, and nocodazole had no effect (Figure S6H). Thus, the reversal of senescence by LFU was different from the activation of tumor cell apoptosis since they involved different cytoskeletal elements (Chitaley and Webb 2001).

### 2.13 Expansion of Replicative Senescent Cells by LFU

**FIGURE 5:**
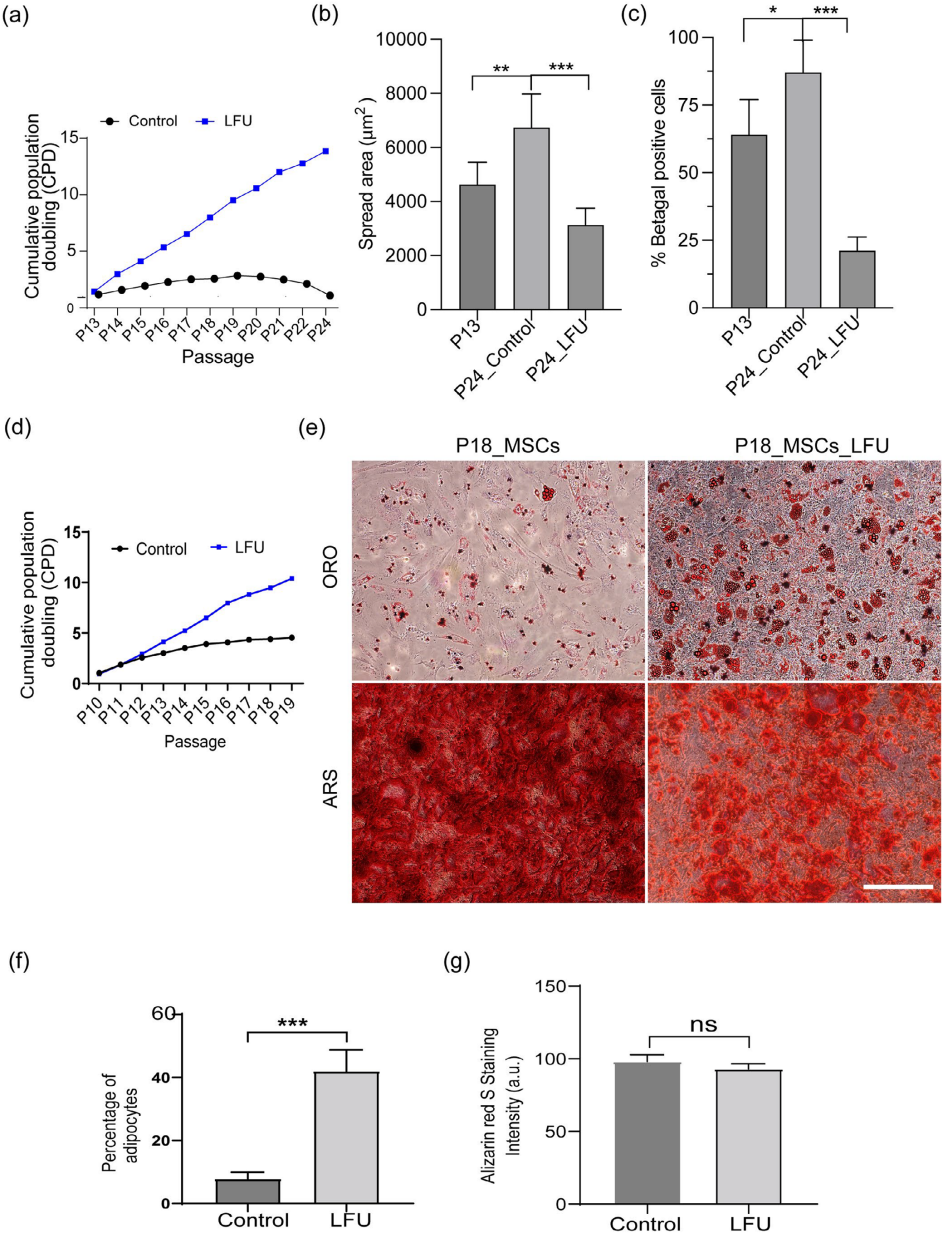
Ultrasound reversal of replicative senescence increases the number of cells. (a) Growth rate is shown as cumulative population doublings (CPD) for Control HFF and LFU treated HFF cells passaged every 48 h from P13 to P24 passage. LFU treatment was applied every other passage. (b) LFU treated cells were smaller than the P24 control and even than P13 cells. (c) Percentage of SA‐β‐galactosidase positive cells decreased after LFU treatment. (d) Similarly, LFU treatment of mesenchymal stem cells (MSCs) expanded the cell number between P10 and P19 passages. LFU treatment was also applied every other passage. (e) LFU treated MSCs showed normal differentiation to (ORO) adipocytes or (ARS) osteocytes. Oil red O staining dye marked lipid droplets (ORO) and alizarin red S dye marked osteogenesis (ARS). (f) Percentage of adipocytes were quantified in P18 MSCs treated with and without LFU. Results are plotted as mean of three independent experiments, a minimum of 100 cells were counted. (g) Quantification of osteocytes was determined by the intensity of alizarine Red S staining. Mean intensity was calculated from 10 random images of three independent experiments. Results are shown as mean ± SD, a minimum of 200 cells for spread area and 150 cells for percentage β‐galactosidase analysis were assessed, *n* > 3 experiments. Significance was determined using two tailed unpaired t‐test. *p < 0.05, **p < 0.002, ***p < 0.0001.

Because replicative senescence commonly limited normal cell growth (Hayflick and Moorhead 1961), LFU could possibly extend the growth of normal cells. After 13 passages, HFFs grew slowly, had a greater average cell size, and increased β‐galactosidase activity (Figure 5a–c). After LFU treatment at every other passage, for passages 15–24, HFFs behaved like normal cells and continued to grow without a significant change in growth rate until at least passage 24 (Figure 5a). This made it possible to grow > 8000 (2^13^)‐fold more HFFs than without LFU, while spread area was normal (Figure 5b) as was the level of β‐galactosidase (Figure 5c). When P24 LFU‐treated cells were cultured on soft matrices, they ceased to grow, showing that expanded cells still maintained rigidity‐dependent growth (Figure S13). Similarly, we expanded MSCs with LFU beyond their normal replicative limit (Figure 5d). Upon treatment with differentiation medium, the LFU‐expanded P18‐MSCs differentiated into adipocytes or osteocytes, depending upon the medium (Figure 5e–g) although the level of adipocyte differentiation was higher in LFU‐treated cells than in replicatively senescent MSCs.

### 2.14 Mouse Healthspan and Lifespan Are Improved by LFU in a Dose‐Dependent Manner

To determine if LFU can be utilized in vivo to improve the performance of older mice, we followed a sham‐treated (placed in the water bath without LFU for 30 min every day) and 5 LFU‐treated groups of mice (*n* = 10 mice per group) (the 5 LFU‐treated groups had different dose schedules: either daily (D1), every other day (D2), or every third day (D3) with 1X power or daily with 1.3X (1.3X) or 2X (2X) power levels for 30 min) (Figure S8A). After two treatment periods of 2 weeks each, with a 2‐week break between periods, physical performance improved with all LFU conditions and was statistically significant for all groups on the treadmill and 3 of 5 in the inverted cling test (Figure S8B,C). Plotting the performance of the male mice separately from the females revealed no consistent differences in the improvement with LFU (Figure S8D–G). While LFU treatment for 4 weeks appeared to be more effective than treatment for 2 weeks, there was no clear difference between the different regimens.

We sacrificed two mice from each group 5 days after the last LFU treatment and inspected their kidneys and pancreas (Figure S9A). After sectioning, fixing, and staining for β‐galactosidase, ~70% of the kidney area and ~70% of the pancreas area were stained in sham animals (Figure S9B–E). In contrast, sections of organs from LFU‐treated animals had only 10%–20% of the area stained for β‐galactosidase (Figure S9B–E). Overall, LFU‐treated animals had a significantly smaller fraction of cells in these two tissues expressing β‐galactosidase, which correlated with improved performance.

In an experiment to determine if LFU decreased senescence markers in 22‐month‐old mice, they were treated for two rounds (separated by 30 days) of 10 LFU treatments every third day. After 20 treatments, treated mice and sham‐treated controls were sacrificed, organs were removed, and flash frozen. Frozen organs were sectioned and processed by freeze substitution for immunostaining with antibodies to p21 and p16 markers of senescence and analyzed by super‐resolution confocal microscopy (Figure S10A,D). There was a consistent increase in the p16 and p21 staining intensity in the aged mice compared with the young mice, and LFU treatment of the old mice decreased the level of staining to that of the young mice or less in some cases (Figure S10B,C,E,F). Males showed no consistent difference from females. Thus, although individual cell senescence was not measured in the organs (kidney and pancreas), it was clear that the expression of the senescence marker proteins, p16 and p21, was much greater in older mice and was reversed to near normal levels by LFU.

**FIGURE 6:**
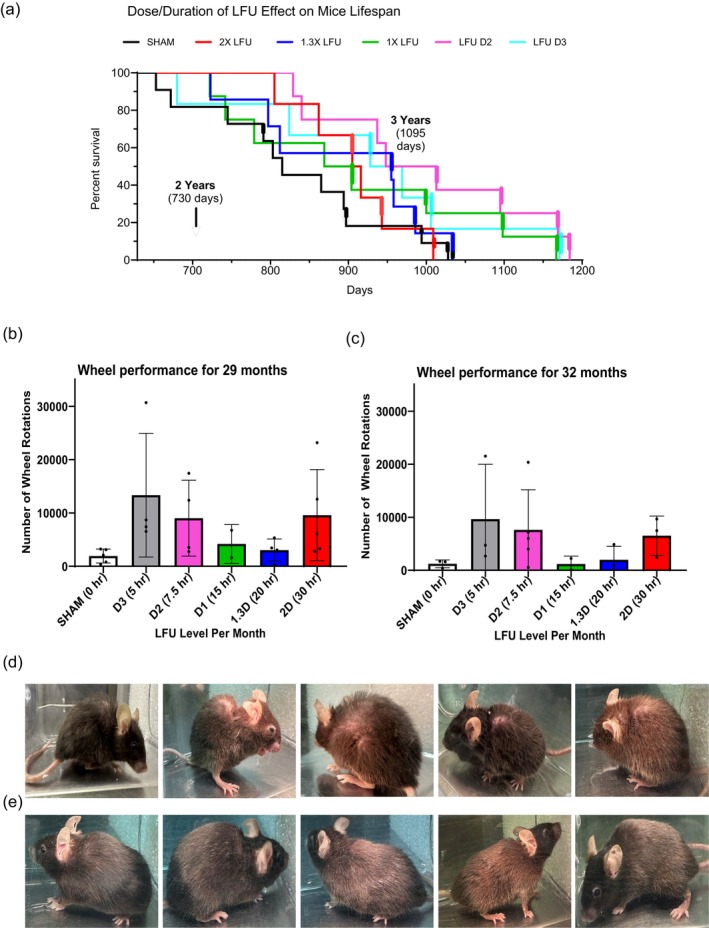
LFU significantly improves the physical performance and lifespan of old mice. (a), The graph shows the percentage survival curves of sham (11 mice) and LFU treated mice (with increasing LFU dosages, D3, D2, D1, 1.3X, and 2X). There were 8‐D1 mice with daily, 8‐D2 mice with every 2nd day, 6‐D3 mice with every 3rd day treatment at 1X power, plus 7–1.3X mice at 1.3X power and 6‐2X mice at 2X power treated daily. The sex of the mouse that died is denoted by an arrow for females and a vertical line for males. (b, c) Bar graphs showing mouse wheel running activity for sham, and all LFU treated mice cohorts at (c) 29 months and (d) 32 months age. Mice were treated with various LFU doses and then placed in the wheel cages. Wheel activity was measured for 3 days. Results are plotted as mean ± SD. Sham mice *n* = 6 and LFU treated mice *n* = 6–8, *p < 0.05 using two tailed unpaired *t*‐test. (d) The pictures show the side view of sham and (e), 2X LFU treated mice at 30 months of age. There is a video of a representative sham and a 2X mouse at 30 months that further illustrates the difference in activity of the mice (Videos 6 and 7, respectively). *p < 0.05.

To test the effect of LFU on mouse lifespan, the 46 mice in the six groups outlined above (Figure S8A) were treated over 300 days (some mice reached 3 years of age) and we added 4 mice that were kept in a cage the whole time (negative control group). In the five LFU‐treated groups, the best survivors had the lowest doses of ultrasound (D2 and D3) with about 50% survival at 1000 days (~33 months of age) (Figure 6a) and 3 mice in the D2 cohort of 7 mice survived until 3 years (> 40%). Autopsies of the mice that died revealed no tumors or obvious cause of death. Thus, combined D2 and D3 groups had a statistically significant increase in longevity, but individual groups (7 or 8) did not give statistical significance. There was a significantly greater lifespan for the females, but others have noted that the sex‐related differences are observed in both directions with different species (Austad and Fischer 2016).

The differences in the activity levels of the sham and treated groups were very striking as seen in videos of their movements (Video 6) and by their spontaneous turning of a wheel in their cage (Figure 6b,c). The videos show that two representative sham‐treated mice at 29 months of age were slow in their movements and had poor fur density with a balding spot on their backs (Videos 6 and 7), whereas two representative mice of the 2X power‐treated group were much more attentive and had denser and darker fur coats (Videos 8 and 9). These results were indicative of other members of their respective groups (Figure 6d,e). When the spontaneous activity of each group was measured in a cage with a wheel for 3 days, the LFU‐treated animals were particularly active in the D2 and D3 groups and the level of activity correlated with their survival. At 29 months of age, the sham mice turned the wheel only 1500 turns on average with the best performers at 2000 turns, whereas the D3 LFU‐treated mice had an average of ~12,000 turns with the best at 30,000 (Figure 6c). D2 mice generated 9000 turns on average (Figure 6b) and these two groups had the highest survival levels (Figure 6b). Three months later, the performance of D3 dropped to 10,000 turns and D2 to ~8,000 turns, whereas the sham mice generated ~1,000 turns (Figure 6c). Thus, the activity levels of the LFU‐treated mice were 7–10 fold greater than the activity levels of the sham mice; whereas the survival levels were greater on average and correlated with activity.

**VIDEO 6:** This is the video of 30‐months‐old untreated mouse (Sham1) illustrating low fur density, bald spots, and slow movements. Video content can be viewed at https://onlinelibrary.wiley.com/doi/10.1111/acel.70008

**VIDEO 7:** This is the video of 30‐months‐old untreated mouse (Sham2) showing the low fur density and slow activity. Video content can be viewed at https://onlinelibrary.wiley.com/doi/10.1111/acel.70008

**VIDEO 8:** This video shows a 30‐month‐old mouse treated with LFU (rejuvenated 1) showing thicker and darker fur and increased movement compared to the sham mice. Video content can be viewed at https://onlinelibrary.wiley.com/doi/10.1111/acel.70008

**VIDEO 9:** This video illustrates another 30–months‐old mouse treated with LFU (rejuvenated 2) showing similar outcomes, with thicker and darker fur and increased movement compared to the sham mice. Video content can be viewed at https://onlinelibrary.wiley.com/doi/10.1111/acel.70008

In terms of the safety of LFU, half of the mice in the longevity study were treated daily with LFU for over 300 days without damage or evidence of harm from the LFU treatment. Further, the treated mice maintained a normal weight, whereas the weight of the sham mice was declining with age, and the animals that died had no tumors or obvious cause of death upon autopsy (data not shown). Thus, there was no evidence of damage caused by LFU, and the highest dosage of LFU (2X) had the effect of keeping the fur thick and dark even at 29 months of age (Figure 6e); whereas the sham controls had a bald spot on their backs and a lower fur density (Figure 6d). This strongly supported the hypothesis that LFU rejuvenated the skin and hair cells, enabling them to produce fur like much younger animals.

## 3 Discussion

Senescent cells, as rigorously defined by many markers, including the expression of β‐galactosidase, can be mechanically rejuvenated by LFU without transfection or other biochemical manipulations. The ultrasound pressure waves restore normal behavior irrespective of whether senescence is induced by chemical treatment or by repeated replication. There is no apoptosis with LFU, and videos of senescent cells show a dramatic increase in cell and mitochondrial motility, as well as in growth after LFU treatment. Many features of senescent cells are all reversed by LFU, including the increase in β‐galactosidase activity, p16 and p21 expression, decreased telomere length, increased H3K9me3 levels, decreased 5mC levels, increased cell size, secretion of SASP, and inhibition of growth (summarized in Figure S12). Restoration of normal behavior correlates with a decrease in mitochondrial length and lysosomal volume. We also optimized the values of LFU power, frequency, and duty cycle for rejuvenation of senescent cells, and those values belie an unknown set of processes that are mechanically activated by LFU. Surprisingly, ultrasound treatment of normal cells causes secretion of growth‐stimulating factors that partially restore normal behavior in senescent cells. Because replicatively senescent cells are restored to a normal phenotype by LFU, they can be cultured for longer periods to produce increased numbers of cells without major alteration in their phenotype.

It is perhaps surprising that fully senescent cells can be rejuvenated by pressure waves. This raises the question of how a senescent cell is defined. Cells that were made senescent by toxic compounds or repeated replications were incubated for long periods, and time‐lapse video microscopy verified the absence of any growth. After such treatments, quiescent cells were not present since over 95% of the cells expressed β‐galactosidase (Alessio et al. 2021) and many of the larger senescent cells grew and divided in the videos after LFU. By tracing individual cells, we were able to determine that growth was occurring in over 30% of the originally non‐dividing cells after 4–5 days. Such robust growth is inconsistent with the growth of just a subpopulation of cells that are not senescent. Further, there is no apoptosis after LFU treatment of the senescent cells, and over fifteen characteristics of senescence are reversed. Thus, all of these objective criteria indicate that LFU reverses senescence, and we suggest that LFU actually rejuvenates senescent cells. This opens many new possibilities in the aging research field, including the possibility of rejuvenating aged cells in vivo to inhibit age‐dependent disorders, which appears to be true based on the results of the mouse studies reported here.

The selective lysis of senescent cells is an alternative approach to reducing the effects of aging, and it has been shown to improve the performance of older mice (Chang et al. 2016; Farr et al. 2017; Mendelsohn and Larrick 2018; Zhu et al. 2015). The obvious difficulty is that the loss of senescent cells is hard to reverse. In these longevity studies, LFU treatment increases lifespan and the physical performance of aged mice. Thus, it seems that LFU can be used to rejuvenate aged animals without the use of senolytics.

Mechanical effects on cell behavior have been known for a long time. However, recently it has become clear that controlled mechanical perturbations can reproducibly alter cell functions and phenotypic behaviors. Tumor cells are mechanosensitive since either stretching, fluid shear, or ultrasound can cause apoptosis in vitro (Sheetz 2019; Singh et al. 2021; Tijore et al. 2021). In addition, exercise appears to inhibit tumor growth in vivo (Rundqvist et al. 2020). Normal cells appear to do better with exercise, and myokines that are released with exercise benefit the organism. In the studies presented here, LFU stimulation of normal cells causes the release of beneficial factors that stimulate the growth of senescent cells and perhaps could augment the LFU‐mediated rejuvenation of aged cells in tissues. The released factors from normal cells are not responsible for senescent cell growth in vitro since there are no normal cells in in vitro senescent cell experiments. Instead, LFU‐induced changes in actin dynamics and calcium levels correlate best with the rejuvenation of the senescent cells.

There is evidence of a correlation between exercise and a reversal of senescent cells in older animals and humans (Englund et al. 2021). Individual cells have not been followed in such studies, and it is not clear if exercise reverses senescence or is a senolytic (Chen et al. 2021). Here, it is clear that ultrasound pressure waves alone can reverse senescent cell behavior to that of normal cells; that is, rejuvenate them, without causing cell death in vitro. We suggest that both exercise and LFU can rejuvenate cells in situ without apoptosis and thereby increase the performance of aged animals. Because LFU can easily penetrate the whole human body with only a significant loss of power in bone and lung, it can rejuvenate most of the tissues, including internal organs, such as the pancreas and kidney, as documented here, that are not particularly sensitive to exercise. In this way, LFU may have advantages over exercise to improve healthspan and possibly lifespan. But whatever the intervention is, the critical issue for improving the performance of aged animals is the need to decrease the level of SASP and other inhibitory factors, whether it is through the use of senolytics, exercise, or LFU, or a combination.

The senescent cell state has been extensively studied, but the molecular bases for the changes are not fully understood. It is noteworthy that LFU causes increased cytosolic Ca^2+^ both with and without cytochalasin D, and Ca^2+^ is needed for the activation of autophagy (Velasco‐Estevez et al. 2020). In addition to autophagy, there are major roles for changes in mitophagy in senescence (Carosi et al. 2022; Cho et al. 2017; Escobar et al. 2019; Kaushik et al. 2021; Liu et al. 2018; Xu and Wan 2022). Accordingly, most models of the senescence process postulate complicated roles for autophagy and mitophagy to couple changes in the activity of lysosomes, mitochondria, and other cellular organelles with the cell cycle (Huang et al. 2022; Moltedo et al. 2019). The subcellular effects of ultrasound appear to be mediated by actin‐cytoskeleton and Ca^2+^ effects on mechanically dependent mitochondria‐ER‐lysosome interactions, which activate lysosomal autophagy and mitochondrial fission that are requisite for mitophagy. Thus, we suggest that LFU‐induced physical distortions act on organized elements of the cytoplasm to reverse molecular complexes induced by aging, and the great enhancement with low concentrations of cytochalasin D indicates that actin cytoskeleton complexes are an important part. Similarly, the increase in motile activity caused by LFU indicates that there is a general effect on cells to restore normal functions that are slowed in senescent cells by the accumulation of damaged material. At a molecular level, senescence is associated with active mTORC1 binding to lysosomes, thereby inactivating autophagy (Bartolome et al. 2017; Zi et al. 2022). Rapamycin inhibition of mTORC1 is synergistic with LFU‐induced rejuvenation, and rejuvenation involves activation of autophagy plus a decrease in lysosomal staining. In the case of SIRT1, loss of activity is associated with an increase in senescence, which fits with the need for SIRT1 activity in rejuvenation through increased autophagy (Liu et al. 2018). In addition, AMPK plays a major role in mitophagy and autophagy, and its activation by AICAR increases the performance of aged mice (Kobilo et al. 2014). We show here that piezo1 ion channel activity is needed for optimal LFU rejuvenation of senescent cells, and it appears to have a role in endothelial cell aging (Xiao et al. 2023). The Ruthenium red and GsMTx4 inhibitors may have effects on multiple targets, and the level of cytoplasmic calcium involved in rejuvenation is not known; but with cytochalasin D, the cytoplasmic calcium levels are low. In general, much more research is needed to understand the detailed mechanisms by which LFU‐induced pressure waves activate autophagy, inhibit mTORC1, and activate SIRT1 function to stimulate cell growth.

Cellular changes with senescence are extensive and involve not only changes in organelle architecture but also in the secretory pathways that produce SASP. The surprising finding that LFU treatment of non‐senescent cells induces the secretion of growth stimulatory factor(s) indicates that the mechanical effects of LFU may be part of a larger network of functions that support systemic responses to physical activity. For example, exercise stimulates the secretion of myokines that benefit brain function and quality of life (So et al. 2014). We suggest that the effects of LFU may mimic some effects of physical activity at a cellular level.

The activation of the growth of replicative senescent cells by non‐invasive LFU has important implications for the in vitro expansion of normal cells to aid in autologous repair procedures, and it can augment the effects of nicotinamide on replicative senescence (Lim et al. 2006). Much more research is needed to understand the extent of the expansion that is possible. However, LFU‐activated expansion does not involve apparent damage to the cells or major modifications of their phenotype. This indicates that LFU‐induced reversal of senescence can have significant benefits in enabling the continued growth of normal cells beyond current limits.

As ultrasound has been approved for human exposure at power levels 10‐ to 100‐fold higher than the optimal levels used in this study, we suggest that it is practical to develop ultrasound‐based therapies that could inhibit (or reverse) the increase in senescent cells in tissues with aging and thereby inhibit the onset of many age‐related maladies. This non‐invasive procedure has advantages over senolytics in that it is not tissue selective. In addition, LFU is non‐invasive and will not directly affect biochemical or molecular biological treatments. Most importantly, these results show that mechanical treatments can augment or replace biochemical treatments to produce the desired reversal of senescence, and they are consistent with known effects of physical activity on senescence and quality of life with aging.

## 4 Materials and Methods

### 4.1 Cell Lines and Cell Culture

Human Foreskin Fibroblasts (HFFs) and Bone marrow‐derived mesenchymal stem cells (MSCs) were purchased from the ATCC. African monkey kidney‐derived Vero cells were obtained from the M. Garcia‐Blanco lab. All cell lines were cultured per the manufacturer's protocol. Vero cells and HFFs were in growth medium Dulbecco's Modified Eagle's Medium (DMEM) 10% fetal bovine serum (FBS; Gibco) and 1% Penicillin/Streptomycin. Human MSCs were cultured in MSCs‐approved medium (ATCC) and expanded by the supplier's protocol. Culture medium was changed every 48 h unless otherwise stated. Cells were plated at 20%–40%. Cells were passaged every 48–72 h using Trypsin/EDTA (Gibco).

### 4.2 Senescence Induction and Quantification

Senescence was induced by stressors, including 200 μM H_2_O_2_, 4 mM of sodium butyrate (SB), 25 μM bleomycin sulphate (BS) or 200 nM doxorubicin by 36–48 h incubation. Afterwards, cells were incubated in growth media for 4 days to establish senescence. Senescence was detected by the β‐galactosidase staining kit per the manufacturer's protocol. Briefly, sub‐confluent senescent cells were stained by the SA‐β‐galactosidase staining solution and incubated overnight at 37°C. The β‐galactosidase cells appeared blue and were senescent cells.

### 4.3 LFU Treatment of Cells

Prior to LFU treatment, the plates containing senescent cells were wrapped with parafilm to avoid contamination and water influx into the plate. The samples were placed on a plastic mesh, mounted in a water tank with an ultrasound transducer. Water in the tank was degassed and heated to 35°C. The distance between the sample and transducer was approximately 9–10 cm. Output power of the transducer was measured at the plate location by a calibrated needle hydrophone (ONDA MCT‐2000). Cells were treated with LFU of optimal parameters: power (4 kPa), frequency 32.248 kHz, and a duty cycle of 1.5 on and 1.5 off for 20–30 mins. After LFU treatment, cell density was measured, and plates were incubated for 48 h to determine the growth rate.

### 4.4 Reversal of Senescence

Senescent cells were treated with LFU of optimized parameters (power, frequency, and duty cycle), counted before incubation, and recounted after 48 h to measure growth (passage P0). Cells were then trypsinized, reseeded, counted, and incubated for 48 h for the P1 passage. This process was repeated for subsequent passages. Absence of growth in number, large size, β‐galactosidase expression, and lack of EdU incorporation were measured as parameters that LFU reversed the senescence. Control cells without LFU treatment remained senescent.

Passage 15–24 HFFs were treated with LFU every other passage at optimized frequency and power. Cell proliferation was determined by counting the number of cells at the time of seeding and 48 h post LFU treatment. In the case of control P24 HFFs, they were treated with LFU and incubated for 96 h prior to trypsinization and reseeding. Growth and morphology were measured after 48 h of incubation.

### 4.5 Pharmacological Drug Treatment

The following inhibitors were used in the study: Nocodazole (1 μM), Cytochalasin D (25‐100 nM), Blebbistatin (10 μM), Y27632 (1 μM), EX‐527 (10 μM), GsMTx (1 μM) and Rapamycin (1 μM). Senescent cells were incubated in Resveratrol (100 μM) for 24 h to activate Sirtuin1 activity. L‐Leucine (10 μM) and Rapamycin (200 nM) were used to activate and inhibit phosphorylated mTOR during the 30 min LFU treatments. To assess the LFU effect on TRPV1 activation or inhibition, we used Ruthenium Red (10 μM) and Capsaicin (10 μM) during the LFU treatments. For these treatments, all inhibitors were purchased from Sigma Aldrich and prepared in DMSO and milli‐Q water as per the manufacturer's protocol. Cells were incubated in inhibitors overnight after 6–8 h of cells seeding prior to LFU.

### 4.6 EdU and Annexin V Staining

Cells were incubated with 10 mM EdU reagent for 24 h. Cells were then fixed, permeabilized, and blocked according to the manufacturer's protocol (Click‐iT EdU Alexa Fluor 555 imaging kit, Life Technologies). The number of magenta puncta divided by the total number of blue nuclei gave the percentage of EdU‐positive cells. At least 200–300 cells were counted manually using ImageJ for each analysis. Apoptosis of senescent cells was determined using Annexin V Alexa fluorophore 488 staining solution. Senescent cells were treated with LFU and after 30 m, they were stained for Annexin V according to the manufacturer's protocol, and images were taken with an Evos microscope at 10X. Senescent cells treated with 200 mM H_2_O_2_ were a positive control.

### 4.7 Animals

All mice were purchased from Jackson's lab (JAX 000664 and C57BL/6J strain) and maintained in the Animal Research Center (ARC) at UTMB. All animal‐related procedures, including housing, euthanasia, non‐survival surgery, tissue collections, and experimental procedures, were approved by the Institutional Animal Care and Use Committee (IACUC) in the protocol number 2102013. Each experimental group started with 22–25‐month‐old mice, both males and females. Equal numbers of mice were used in each group unless otherwise stated.

### 4.8 LFU Treatment of Aged Mice

Aged mice (> 22 months old) were treated in a 4 L glass beaker with an internal plastic cylinder of 13 cm height and 15.2 cm in diameter. A plastic mesh was placed on top of the cylinder that supported the mice and enabled them to rest with their four limbs and body in the water. Degassed, 32°C–35°C water filled the beaker to a level 1 in. above the plastic mesh so that half of them were in water. Once in the water, LFU treatment was applied to the mice. After treatment, the animals were placed in a separate cage with tissue paper for drying and then returned to their home cage. Control mice were placed in the same water bath for 30′ without ultrasonication.

### 4.9 Statistical Analysis

All experiments were repeated with a minimum of three samples per group as mentioned in the figure legends. Data was represented as the mean ± standard deviation, and Statistical analysis was performed using GraphPad prism 10.0. Differences in the two groups were determined using the two‐tailed paired, unpaired Student's *t*‐test, Mann Whitney tests, and Kruskal Wallis, and One way ANOVA test followed by post hoc Dunn's test used for the multiple groups. Statistical significance was analyzed and reported in the figures and figure legends: *, *p* < 0.05; **, *p* < 0.002; ***, *p* < 0.001; ****, *p* < 0.0001; and non‐significant (ns) *p* > 0.05.

## Author Contributions

Conceptualization: M.P.S., S.K.K., B.B.R. Methodology: S.K.K., R.M., S.P., F.M. Writing: M.P.S., S.K.K., B.B.R. Experimentation: S.K.K., R.M., B.B., M.A., E.S.

## Conflicts of Interest

Authors (M.S., S.K.K., F.M., B.B.R. and R.M.) are co‐authors of patents related to these studies, and M.S. and F.M. have financial interests in a company, Mechanobiologics Inc. that is planning to market LFU devices suitable for senescent cell rejuvenation in vitro and in vivo.

## Supporting information


Data S1.

Data S2.

## Data Availability

All data are available in the main text or the Supporting Information.

## References

[acel70008-bib-0001] Adams, P. D. 2009. “Healing and Hurting: Molecular Mechanisms, Functions, and Pathologies of Cellular Senescence.” Molecular Cell 36, no. 1: 2–14. 10.1016/j.molcel.2009.09.021. 19818705

[acel70008-bib-0002] Ahmadi, F. , I. V. McLoughlin , S. Chauhan , and G. ter‐Haar . 2012. “Bio‐Effects and Safety of Low‐Intensity, Low‐Frequency Ultrasonic Exposure.” Progress in Biophysics and Molecular Biology 108, no. 3: 119–138. 10.1016/j.pbiomolbio.2012.01.004. 22402278

[acel70008-bib-0003] Alessio, N. , D. Aprile , S. Cappabianca , G. Peluso , G. Di Bernardo , and U. Galderisi . 2021. “Different Stages of Quiescence, Senescence, and Cell Stress Identified by Molecular Algorithm Based on the Expression of Ki67, RPS6, and Beta‐Galactosidase Activity.” International Journal of Molecular Sciences 22, no. 6: 3102. 10.3390/ijms22063102. 33803589 PMC8002939

[acel70008-bib-0004] Austad, S. N. , and K. E. Fischer . 2016. “Sex Differences in Lifespan.” Cell Metabolism 23, no. 6: 1022–1033. 10.1016/j.cmet.2016.05.019. 27304504 PMC4932837

[acel70008-bib-0005] Balbi, M. , D. G. Blackmore , P. Padmanabhan , and J. Gotz . 2022. “Ultrasound‐Mediated Bioeffects in Senescent Mice and Alzheimer's Mouse Models.” Brain Sciences 12, no. 6: 775. 10.3390/brainsci12060775. 35741660 PMC9221310

[acel70008-bib-0006] Bartolak‐Suki, E. , J. Imsirovic , Y. Nishibori , R. Krishnan , and B. Suki . 2017. “Regulation of Mitochondrial Structure and Dynamics by the Cytoskeleton and Mechanical Factors.” International Journal of Molecular Sciences 18, no. 8: 1812. 10.3390/ijms18081812. 28825689 PMC5578198

[acel70008-bib-0007] Bartolome, A. , A. Garcia‐Aguilar , S. I. Asahara , et al. 2017. “MTORC1 Regulates Both General Autophagy and Mitophagy Induction After Oxidative Phosphorylation Uncoupling.” Molecular and Cellular Biology 37, no. 23: e00441‐17. 10.1128/MCB.00441-17. 28894028 PMC5686580

[acel70008-bib-0008] Borghesan, M. , W. M. H. Hoogaars , M. Varela‐Eirin , N. Talma , and M. Demaria . 2020. “A Senescence‐Centric View of Aging: Implications for Longevity and Disease.” Trends in Cell Biology 30, no. 10: 777–791. 10.1016/j.tcb.2020.07.002. 32800659

[acel70008-bib-0009] Brock, M. A. , and F. Chrest . 1993. “Differential Regulation of Actin Polymerization Following Activation of Resting T Lymphocytes From Young and Aged Mice.” Journal of Cellular Physiology 157, no. 2: 367–378. 10.1002/jcp.1041570221. 8227168

[acel70008-bib-0010] Bussian, T. J. , A. Aziz , C. F. Meyer , B. L. Swenson , J. M. van Deursen , and D. J. Baker . 2018. “Clearance of Senescent Glial Cells Prevents Tau‐Dependent Pathology and Cognitive Decline.” Nature 562, no. 7728: 578–582. 10.1038/s41586-018-0543-y. 30232451 PMC6206507

[acel70008-bib-0011] Cai, Y. , H. Zhou , Y. Zhu , et al. 2020. “Elimination of Senescent Cells by Beta‐Galactosidase‐Targeted Prodrug Attenuates Inflammation and Restores Physical Function in Aged Mice.” Cell Research 30, no. 7: 574–589. 10.1038/s41422-020-0314-9. 32341413 PMC7184167

[acel70008-bib-0012] Caland, L. , P. Labbe , M. Mamarbachi , et al. 2019. “Knockdown of Angiopoietin‐Like 2 Induces Clearance of Vascular Endothelial Senescent Cells by Apoptosis, Promotes Endothelial Repair and Slows Atherogenesis in Mice.” Aging (Albany NY) 11, no. 11: 3832–3850. 10.18632/aging.102020. 31186381 PMC6594793

[acel70008-bib-0013] Carosi, J. M. , C. Fourrier , J. Bensalem , and T. J. Sargeant . 2022. “The mTOR‐Lysosome Axis at the Centre of Ageing.” FEBS Open Bio 12, no. 4: 739–757. 10.1002/2211-5463.13347. PMC8972043 34878722

[acel70008-bib-0014] Cassidy, L. D. , and M. Narita . 2020. “Dynamic Modulation of Autophagy: Implications for Aging and Cancer.” Molecular & Cellular Oncology 7, no. 4: 1754723. 10.1080/23723556.2020.1754723. 32944616 PMC7469470

[acel70008-bib-0015] Chae, J. B. , H. Jang , C. Son , et al. 2021. “Targeting Senescent Retinal Pigment Epithelial Cells Facilitates Retinal Regeneration in Mouse Models of Age‐Related Macular Degeneration.” Geroscience 43: 2809–2833. 10.1007/s11357-021-00457-4. 34601706 PMC8602547

[acel70008-bib-0016] Chang, J. , Y. Wang , L. Shao , et al. 2016. “Clearance of Senescent Cells by ABT263 Rejuvenates Aged Hematopoietic Stem Cells in Mice.” Nature Medicine 22, no. 1: 78–83. 10.1038/nm.4010. PMC4762215 26657143

[acel70008-bib-0017] Chen, X. K. , Z. N. Yi , G. T. Wong , et al. 2021. “Is Exercise a Senolytic Medicine? A Systematic Review.” Aging Cell 20, no. 1: e13294. 10.1111/acel.13294. 33378138 PMC7811843

[acel70008-bib-0018] Childs, B. G. , M. Durik , D. J. Baker , and J. M. van Deursen . 2015. “Cellular Senescence in Aging and Age‐Related Disease: From Mechanisms to Therapy.” Nature Medicine 21, no. 12: 1424–1435. 10.1038/nm.4000. PMC4748967 26646499

[acel70008-bib-0019] Childs, B. G. , M. Gluscevic , D. J. Baker , et al. 2017. “Senescent Cells: An Emerging Target for Diseases of Ageing.” Nature Reviews. Drug Discovery 16, no. 10: 718–735. 10.1038/nrd.2017.116. 28729727 PMC5942225

[acel70008-bib-0020] Chitaley, K. , and R. C. Webb . 2001. “Microtubule Depolymerization Facilitates Contraction of Vascular Smooth Muscle via Increased Activation of RhoA/Rho‐Kinase.” Medical Hypotheses 56, no. 3: 381–385. 10.1054/mehy.2000.1207. 11359365

[acel70008-bib-0021] Cho, J. H. , G. Y. Kim , C. J. Pan , et al. 2017. “Downregulation of SIRT1 Signaling Underlies Hepatic Autophagy Impairment in Glycogen Storage Disease Type Ia.” PLoS Genetics 13, no. 5: e1006819. 10.1371/journal.pgen.1006819. 28558013 PMC5469511

[acel70008-bib-0022] Chubanava, S. , and J. T. Treebak . 2023. “Regular Exercise Effectively Protects Against the Aging‐Associated Decline in Skeletal Muscle NAD Content.” Experimental Gerontology 173: 112109. 10.1016/j.exger.2023.112109. 36708750

[acel70008-bib-0023] Coppe, J. P. , C. K. Patil , F. Rodier , et al. 2008. “Senescence‐Associated Secretory Phenotypes Reveal Cell‐Nonautonomous Functions of Oncogenic RAS and the p53 Tumor Suppressor.” PLoS Biology 6, no. 12: 2853–2868. 10.1371/journal.pbio.0060301. 19053174 PMC2592359

[acel70008-bib-0024] da Silva, P. F. L. , M. Ogrodnik , O. Kucheryavenko , et al. 2019. “The Bystander Effect Contributes to the Accumulation of Senescent Cells In Vivo.” Aging Cell 18, no. 1: e12848. 10.1111/acel.12848. 30462359 PMC6351849

[acel70008-bib-0025] De Sousa Lages, A. , V. Lopes , J. Horta , J. Espregueira‐Mendes , R. Andrade , and A. Rebelo‐Marques . 2022. “Therapeutics That Can Potentially Replicate or Augment the Anti‐Aging Effects of Physical Exercise.” International Journal of Molecular Sciences 23, no. 17: 9957. 10.3390/ijms23179957. 36077358 PMC9456478

[acel70008-bib-0026] Demaria, M. , N. Ohtani , S. A. Youssef , et al. 2014. “An Essential Role for Senescent Cells in Optimal Wound Healing Through Secretion of PDGF‐AA.” Developmental Cell 31, no. 6: 722–733. 10.1016/j.devcel.2014.11.012. 25499914 PMC4349629

[acel70008-bib-0027] Englund, D. A. , A. E. Sakamoto , C. M. Fritsche , et al. 2021. “Exercise Reduces Circulating Biomarkers of Cellular Senescence in Humans.” Aging Cell 20, no. 7: e13415. 10.1111/acel.13415. 34101960 PMC8282238

[acel70008-bib-0028] Escobar, K. A. , N. H. Cole , C. M. Mermier , and T. A. VanDusseldorp . 2019. “Autophagy and Aging: Maintaining the Proteome Through Exercise and Caloric Restriction.” Aging Cell 18, no. 1: e12876. 10.1111/acel.12876. 30430746 PMC6351830

[acel70008-bib-0029] Ezquer, M. E. , S. R. Valdez , A. M. Seltzer , and G. A. Jahn . 2014. “Reversion by Vitamin E Treatment of the Oxidative Damage but Not of the Advancement in Reproductive Senescence Produced by Neonatal Hypoxia or Hypoxia‐Ischemia in Female Rats.” Neuroendocrinology 99, no. 3–4: 204–218. 10.1159/000365448. 25011732

[acel70008-bib-0030] Farr, J. N. , M. Xu , M. M. Weivoda , et al. 2017. “Targeting Cellular Senescence Prevents Age‐Related Bone Loss in Mice.” Nature Medicine 23, no. 9: 1072–1079. 10.1038/nm.4385. PMC5657592 28825716

[acel70008-bib-0031] Galkin, F. , O. Kovalchuk , D. Koldasbayeva , A. Zhavoronkov , and E. Bischof . 2023. “Stress, Diet, Exercise: Common Environmental Factors and Their Impact on Epigenetic Age.” Ageing Research Reviews 88: 101956. 10.1016/j.arr.2023.101956. 37211319

[acel70008-bib-0032] Gentilini, D. , D. Mari , D. Castaldi , et al. 2013. “Role of Epigenetics in Human Aging and Longevity: Genome‐Wide DNA Methylation Profile in Centenarians and Centenarians' Offspring.” Age (Dordrecht, Netherlands) 35, no. 5: 1961–1973. 10.1007/s11357-012-9463-1. 22923132 PMC3776126

[acel70008-bib-0033] Gnanasambandam, R. , P. A. Gottlieb , and F. Sachs . 2017. “The Kinetics and the Permeation Properties of Piezo Channels.” In Current Topics in Membranes, vol. 79, 275–307. Elsevier. 10.1016/bs.ctm.2016.11.004. 28728821

[acel70008-bib-0034] Hayflick, L. , and P. S. Moorhead . 1961. “The Serial Cultivation of Human Diploid Cell Strains.” Experimental Cell Research 25: 585–621. 10.1016/0014-4827(61)90192-6. 13905658

[acel70008-bib-0035] He, S. , and N. E. Sharpless . 2017. “Senescence in Health and Disease.” Cell 169, no. 6: 1000–1011. 10.1016/j.cell.2017.05.015. 28575665 PMC5643029

[acel70008-bib-0036] Helle, S. C. J. , Q. Feng , M. J. Aebersold , et al. 2017. “Mechanical Force Induces Mitochondrial Fission.” eLife 6: e30292. 10.7554/eLife.30292. 29119945 PMC5679753

[acel70008-bib-0037] Hernandez‐Segura, A. , J. Nehme , and M. Demaria . 2018. “Hallmarks of Cellular Senescence.” Trends in Cell Biology 28, no. 6: 436–453. 10.1016/j.tcb.2018.02.001. 29477613

[acel70008-bib-0038] Herranz, N. , and J. Gil . 2018. “Mechanisms and Functions of Cellular Senescence.” Journal of Clinical Investigation 128, no. 4: 1238–1246. 10.1172/JCI95148. 29608137 PMC5873888

[acel70008-bib-0039] Huang, J. , P. Meng , C. Wang , Y. Zhang , and L. Zhou . 2022. “The Relevance of Organelle Interactions in Cellular Senescence.” Theranostics 12, no. 5: 2445–2464. 10.7150/thno.70588. 35265219 PMC8899567

[acel70008-bib-0040] Jeanblanc, M. , S. Ragu , C. Gey , et al. 2012. “Parallel Pathways in RAF‐Induced Senescence and Conditions for Its Reversion.” Oncogene 31, no. 25: 3072–3085. 10.1038/onc.2011.481. 22020327

[acel70008-bib-0041] Jeon, O. H. , C. Kim , R. M. Laberge , et al. 2017. “Local Clearance of Senescent Cells Attenuates the Development of Post‐Traumatic Osteoarthritis and Creates a Pro‐Regenerative Environment.” Nature Medicine 23, no. 6: 775–781. 10.1038/nm.4324. PMC5785239 28436958

[acel70008-bib-0042] Kaushik, S. , I. Tasset , E. Arias , et al. 2021. “Autophagy and the Hallmarks of Aging.” Ageing Research Reviews 72: 101468. 10.1016/j.arr.2021.101468. 34563704 PMC8616816

[acel70008-bib-0043] Kirkland, J. L. , and T. Tchkonia . 2020. “Senolytic Drugs: From Discovery to Translation.” Journal of Internal Medicine 288, no. 5: 518–536. 10.1111/joim.13141. 32686219 PMC7405395

[acel70008-bib-0044] Kirkland, J. L. , T. Tchkonia , Y. Zhu , L. J. Niedernhofer , and P. D. Robbins . 2017. “The Clinical Potential of Senolytic Drugs.” Journal of the American Geriatrics Society 65, no. 10: 2297–2301. 10.1111/jgs.14969. 28869295 PMC5641223

[acel70008-bib-0045] Kobilo, T. , D. Guerrieri , Y. Zhang , S. C. Collica , K. G. Becker , and H. van Praag . 2014. “AMPK Agonist AICAR Improves Cognition and Motor Coordination in Young and Aged Mice.” Learning & Memory 21, no. 2: 119–126. 10.1101/lm.033332.113. 24443745 PMC3895225

[acel70008-bib-0046] Krimpenfort, P. , and A. Berns . 2017. “Rejuvenation by Therapeutic Elimination of Senescent Cells.” Cell 169, no. 1: 3–5. 10.1016/j.cell.2017.03.014. 28340347

[acel70008-bib-0047] Kvell, K. , and J. E. Pongracz . 2012. “Central Immune Senescence, Reversal Potentials.” In Senescence, edited by T. Nagata . InTech. 28045481

[acel70008-bib-0048] Le, Q. V. , S. Y. Wen , C. J. Chen , C. Y. Huang , and W. W. Kuo . 2022. “Reversion of Glucocorticoid‐Induced Senescence and Collagen Synthesis Decrease by LY294002 Is Mediated Through p38 in Skin.” International Journal of Biological Sciences 18, no. 16: 6102–6113. 10.7150/ijbs.73915. 36439879 PMC9682531

[acel70008-bib-0049] Lefevre, C. , M. Auclair , F. Boccara , et al. 2010. “Premature Senescence of Vascular Cells Is Induced by HIV Protease Inhibitors: Implication of Prelamin A and Reversion by Statin.” Arteriosclerosis, Thrombosis, and Vascular Biology 30, no. 12: 2611–2620. 10.1161/ATVBAHA.110.213603. 20884875

[acel70008-bib-0050] Leighton, R. , J. T. Watson , P. Giannoudis , C. Papakostidis , A. Harrison , and R. G. Steen . 2017. “Healing of Fracture Nonunions Treated With Low‐Intensity Pulsed Ultrasound (LIPUS): A Systematic Review and Meta‐Analysis.” Injury 48, no. 7: 1339–1347. 10.1016/j.injury.2017.05.016. 28532896

[acel70008-bib-0051] Li, G. , Q. Zhu , B. Wang , et al. 2021. “Rejuvenation of Senescent Bone Marrow Mesenchymal Stromal Cells by Pulsed Triboelectric Stimulation.” Advanced Science 8, no. 18: e2100964. 10.1002/advs.202100964. 34258884 PMC8456218

[acel70008-bib-0052] Libertini, G. , N. Ferrara , G. Rengo , and G. Corbi . 2018. “Elimination of Senescent Cells: Prospects According to the Subtelomere‐Telomere Theory.” Biochemistry (Mosc) 83, no. 12: 1477–1488. 10.1134/S0006297918120064. 30878023

[acel70008-bib-0053] Lim, C. S. , M. Potts , and R. F. Helm . 2006. “Nicotinamide Extends the Replicative Life Span of Primary Human Cells.” Mechanisms of Ageing and Development 127, no. 6: 511–514. 10.1016/j.mad.2006.02.001. 16545428

[acel70008-bib-0054] Liu, T. , X. Ma , T. Ouyang , et al. 2018. “SIRT1 Reverses Senescence via Enhancing Autophagy and Attenuates Oxidative Stress‐Induced Apoptosis Through Promoting p53 Degradation.” International Journal of Biological Macromolecules 117: 225–234. 10.1016/j.ijbiomac.2018.05.174. 29803744

[acel70008-bib-0055] Lopez‐Otin, C. , M. A. Blasco , L. Partridge , M. Serrano , and G. Kroemer . 2013. “The Hallmarks of Aging.” Cell 153, no. 6: 1194–1217. 10.1016/j.cell.2013.05.039. 23746838 PMC3836174

[acel70008-bib-0056] Lorenzo, E. C. , B. L. Torrance , and L. Haynes . 2023. “Impact of Senolytic Treatment on Immunity, Aging, and Disease.” Frontiers in Aging 4: 1161799. 10.3389/fragi.2023.1161799. 37886012 PMC10598643

[acel70008-bib-0057] McHugh, D. , and J. Gil . 2018. “Senescence and Aging: Causes, Consequences, and Therapeutic Avenues.” Journal of Cell Biology 217, no. 1: 65–77. 10.1083/jcb.201708092. 29114066 PMC5748990

[acel70008-bib-0058] Mendelsohn, A. R. , and J. W. Larrick . 2018. “Prevention of Senescence in Vasculature Through Quiescence.” Rejuvenation Research 21, no. 5: 477–481. 10.1089/rej.2018.2138. 30259779

[acel70008-bib-0059] Moltedo, O. , P. Remondelli , and G. Amodio . 2019. “The Mitochondria‐Endoplasmic Reticulum Contacts and Their Critical Role in Aging and Age‐Associated Diseases.” Frontiers in Cell and Development Biology 7: 172. 10.3389/fcell.2019.00172. PMC6712070 31497601

[acel70008-bib-0060] Munoz‐Espin, D. , and M. Serrano . 2014. “Cellular Senescence: From Physiology to Pathology.” Nature Reviews. Molecular Cell Biology 15, no. 7: 482–496. 10.1038/nrm3823. 24954210

[acel70008-bib-0061] Nadri, P. , S. Ansari‐Mahyari , F. Jafarpour , et al. 2022. “Melatonin Accelerates the Developmental Competence and Telomere Elongation in Ovine SCNT Embryos.” PLoS One 17, no. 7: e0267598. 10.1371/journal.pone.0267598. 35862346 PMC9302776

[acel70008-bib-0062] Neves, J. , P. Sousa‐Victor , and H. Jasper . 2017. “Rejuvenating Strategies for Stem Cell‐Based Therapies in Aging.” Cell Stem Cell 20, no. 2: 161–175. 10.1016/j.stem.2017.01.008. 28157498 PMC5681350

[acel70008-bib-0063] Ning, K. , Z. Wang , and X. A. Zhang . 2022. “Exercise‐Induced Modulation of Myokine Irisin in Bone and Cartilage Tissue‐Positive Effects on Osteoarthritis: A Narrative Review.” Frontiers in Aging Neuroscience 14: 934406. 10.3389/fnagi.2022.934406. 36062149 PMC9439853

[acel70008-bib-0064] Patil, P. , Q. Dong , D. Wang , et al. 2019. “Systemic Clearance of p16(INK4a) ‐Positive Senescent Cells Mitigates Age‐Associated Intervertebral Disc Degeneration.” Aging Cell 18, no. 3: e12927. 10.1111/acel.12927. 30900385 PMC6516165

[acel70008-bib-0065] Peilin, W. , T. Songsong , Z. Chengyu , et al. 2019. “Directed Elimination of Senescent Cells Attenuates Development of Osteoarthritis by Inhibition of c‐IAP and XIAP.” Biochimica et Biophysica Acta ‐ Molecular Basis of Disease 1865, no. 10: 2618–2632. 10.1016/j.bbadis.2019.05.017. 31251987

[acel70008-bib-0066] Penney, J. , and L. H. Tsai . 2018. “Elimination of Senescent Cells Prevents Neurodegeneration in Mice.” Nature 562, no. 7728: 503–504. 10.1038/d41586-018-06677-7. 30349121

[acel70008-bib-0067] Rubinsztein, D. C. , G. Marino , and G. Kroemer . 2011. “Autophagy and Aging.” Cell 146, no. 5: 682–695. 10.1016/j.cell.2011.07.030. 21884931

[acel70008-bib-0068] Rundqvist, H. , P. Velica , L. Barbieri , et al. 2020. “Cytotoxic T‐Cells Mediate Exercise‐Induced Reductions in Tumor Growth.” eLife 9: e59996. 10.7554/eLife.59996. 33095157 PMC7584454

[acel70008-bib-0069] Selle, A. , O. Ullrich , K. Harnacke , and R. Hass . 2007. “Retrodifferentiation and Rejuvenation of Senescent Monocytic Cells Requires PARP‐1.” Experimental Gerontology 42, no. 6: 554–562. 10.1016/j.exger.2006.12.004. 17314023

[acel70008-bib-0070] Sheetz, M. 2019. “A Tale of Two States: Normal and Transformed, With and Without Rigidity Sensing.” Annual Review of Cell and Developmental Biology 35: 169–190. 10.1146/annurev-cellbio-100818-125227. PMC7474971 31412209

[acel70008-bib-0071] Singh, A. , A. Tijore , F. Margadant , et al. 2021. “Enhanced Tumor Cell Killing by Ultrasound After Microtubule Depolymerization.” Bioengineering & Translational Medicine 6: e10233. 10.1002/btm2.10233. 34589605 PMC8459596

[acel70008-bib-0072] So, B. , H. J. Kim , J. Kim , and W. Song . 2014. “Exercise‐Induced Myokines in Health and Metabolic Diseases.” Integrative Medicine Research 3, no. 4: 172–179. 10.1016/j.imr.2014.09.007. 28664094 PMC5481763

[acel70008-bib-0073] Storer, M. , A. Mas , A. Robert‐Moreno , et al. 2013. “Senescence Is a Developmental Mechanism That Contributes to Embryonic Growth and Patterning.” Cell 155, no. 5: 1119–1130. 10.1016/j.cell.2013.10.041. 24238961

[acel70008-bib-0074] Sukumaran, P. , V. Nascimento Da Conceicao , Y. Sun , et al. 2021. “Calcium Signaling Regulates Autophagy and Apoptosis.” Cells 10, no. 8: 2125. 10.3390/cells10082125. 34440894 PMC8394685

[acel70008-bib-0075] Tchkonia, T. , Y. Zhu , J. van Deursen , J. Campisi , and J. L. Kirkland . 2013. “Cellular Senescence and the Senescent Secretory Phenotype: Therapeutic Opportunities.” Journal of Clinical Investigation 123, no. 3: 966–972. 10.1172/JCI64098. 23454759 PMC3582125

[acel70008-bib-0076] Thompson, P. J. , A. Shah , V. Ntranos , F. Van Gool , M. Atkinson , and A. Bhushan . 2019. “Targeted Elimination of Senescent Beta Cells Prevents Type 1 Diabetes.” Cell Metabolism 29, no. 5: 1045–1060. 10.1016/j.cmet.2019.01.021. 30799288

[acel70008-bib-0077] Tijore, A. , M. Yao , Y. H. Wang , et al. 2021. “Selective Killing of Transformed Cells by Mechanical Stretch.” Biomaterials 275: 120866. 10.1016/j.biomaterials.2021.120866. 34044258

[acel70008-bib-0078] Velasco‐Estevez, M. , S. O. Rolle , M. Mampay , K. K. Dev , and G. K. Sheridan . 2020. “Piezo1 Regulates Calcium Oscillations and Cytokine Release From Astrocytes.” Glia 68, no. 1: 145–160. 10.1002/glia.23709. 31433095

[acel70008-bib-0079] Wang, L. , J. Wei , A. Da Fonseca Ferreira , et al. 2020. “Rejuvenation of Senescent Endothelial Progenitor Cells by Extracellular Vesicles Derived From Mesenchymal Stromal Cells.” JACC. Basic to Translational Science 5, no. 11: 1127–1141. 10.1016/j.jacbts.2020.08.005. 33294742 PMC7691285

[acel70008-bib-0080] Xiao, T. , M. Sun , C. Zhao , and J. Kang . 2023. “TRPV1: A Promising Therapeutic Target for Skin Aging and Inflammatory Skin Diseases.” Frontiers in Pharmacology 14: 1037925. 10.3389/fphar.2023.1037925. 36874007 PMC9975512

[acel70008-bib-0081] Xu, M. , E. W. Bradley , M. M. Weivoda , et al. 2017. “Transplanted Senescent Cells Induce an Osteoarthritis‐Like Condition in Mice.” Journals of Gerontology. Series A, Biological Sciences and Medical Sciences 72, no. 6: 780–785. 10.1093/gerona/glw154. 27516624 PMC5861939

[acel70008-bib-0082] Xu, M. , T. Pirtskhalava , J. N. Farr , et al. 2018. “Senolytics Improve Physical Function and Increase Lifespan in Old Age.” Nature Medicine 24, no. 8: 1246–1256. 10.1038/s41591-018-0092-9. PMC6082705 29988130

[acel70008-bib-0083] Xu, Y. , and W. Wan . 2022. “Acetylation in the Regulation of Autophagy.” Autophagy 1‐9: 379–387. 10.1080/15548627.2022.2062112. PMC9851266 35435793

[acel70008-bib-0084] Yamaura, K. , A. L. Nelson , H. Nishimura , et al. 2023. “Therapeutic Potential of Senolytic Agent Quercetin in Osteoarthritis: A Systematic Review and Meta‐Analysis of Preclinical Studies.” Ageing Research Reviews 90: 101989. 10.1016/j.arr.2023.101989. 37442369

[acel70008-bib-0085] Yao, M. , A. Tijore , D. Cheng , et al. 2022. “Force‐ and Cell State‐Dependent Recruitment of Piezo1 Drives Focal Adhesion Dynamics and Calcium Entry.” Science Advances 8, no. 45: eabo1461. 10.1126/sciadv.abo1461. 36351022 PMC9645726

[acel70008-bib-0086] Young, A. R. , and M. Narita . 2009. “SASP Reflects Senescence.” EMBO Reports 10, no. 3: 228–230. 10.1038/embor.2009.22. 19218920 PMC2658552

[acel70008-bib-0087] Yousefzadeh, M. J. , Y. Zhu , S. J. McGowan , et al. 2018. “Fisetin Is a Senotherapeutic That Extends Health and Lifespan.” eBioMedicine 36: 18–28. 10.1016/j.ebiom.2018.09.015. 30279143 PMC6197652

[acel70008-bib-0088] Zhang, W. H. , S. Koyuncu , and D. Vilchez . 2022. “Insights Into the Links Between Proteostasis and Aging From *C. elegans* .” Frontiers in Aging 3: 854157. 10.3389/fragi.2022.854157. 35821832 PMC9261386

[acel70008-bib-0089] Zhu, Y. , T. Tchkonia , T. Pirtskhalava , et al. 2015. “The Achilles' Heel of Senescent Cells: From Transcriptome to Senolytic Drugs.” Aging Cell 14, no. 4: 644–658. 10.1111/acel.12344. 25754370 PMC4531078

[acel70008-bib-0090] Zi, Z. , Z. Zhang , Q. Feng , et al. 2022. “Quantitative Phosphoproteomic Analyses Identify STK11IP as a Lysosome‐Specific Substrate of mTORC1 That Regulates Lysosomal Acidification.” Nature Communications 13, no. 1: 1760. 10.1038/s41467-022-29461-8. PMC8976005 35365663

