# Supplementary material for: Rejuvenation of Senescent Cells, In Vitro and In Vivo, by Low‐Frequency Ultrasound
Source: Aging Cell. 2025 Mar 3;24(6):e70008. doi: 10.1111/acel.70008 (PMC12151899; doi:10.1111/acel.70008)
Supplement: Supplementary file 1 — Data S1. [file ACEL-24-e70008-s001.docx]

Supplemental-Figures and Tables

S1: Optimization

S2: SCs characterization

S3: LFU activates growth of various SCs types

S4: Calcium and piezo1

S5: SASP

S6: Sirtuins1 and autophagy

S7: Telomere and 5mc

S8: Mice Performance

S9: Beta-gal-Kidney-Pancreas

S10: p16 and p21 staining of kidney and pancreas

S11: Autophagy

S12: Summary figure

S13: Growth on soft surface

Table S1:

Table S2:

D

C

F

E

LFU power and Frequency

LFU power

Duty Cycle

Duration of LFU (mins)

8-9 cm


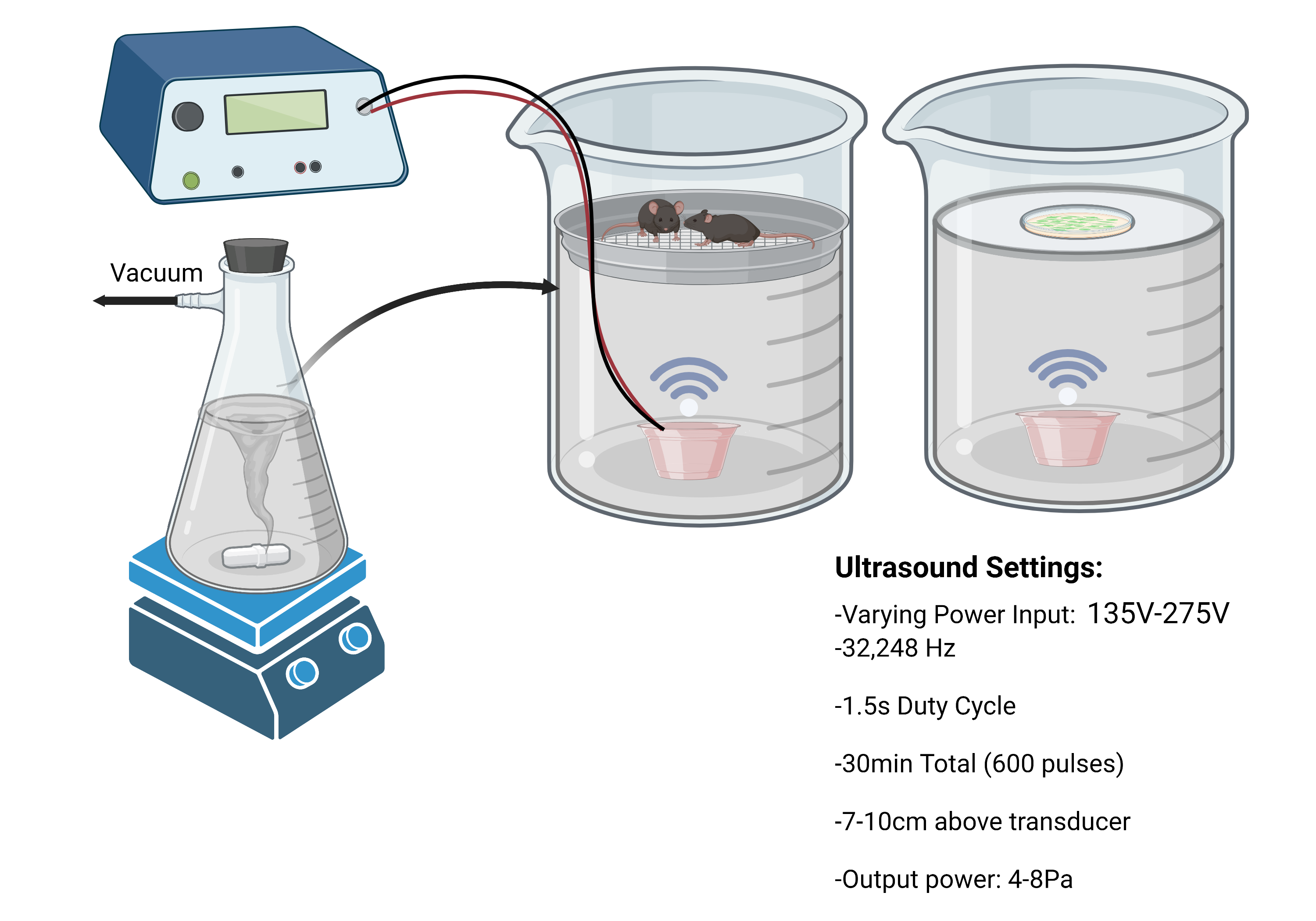

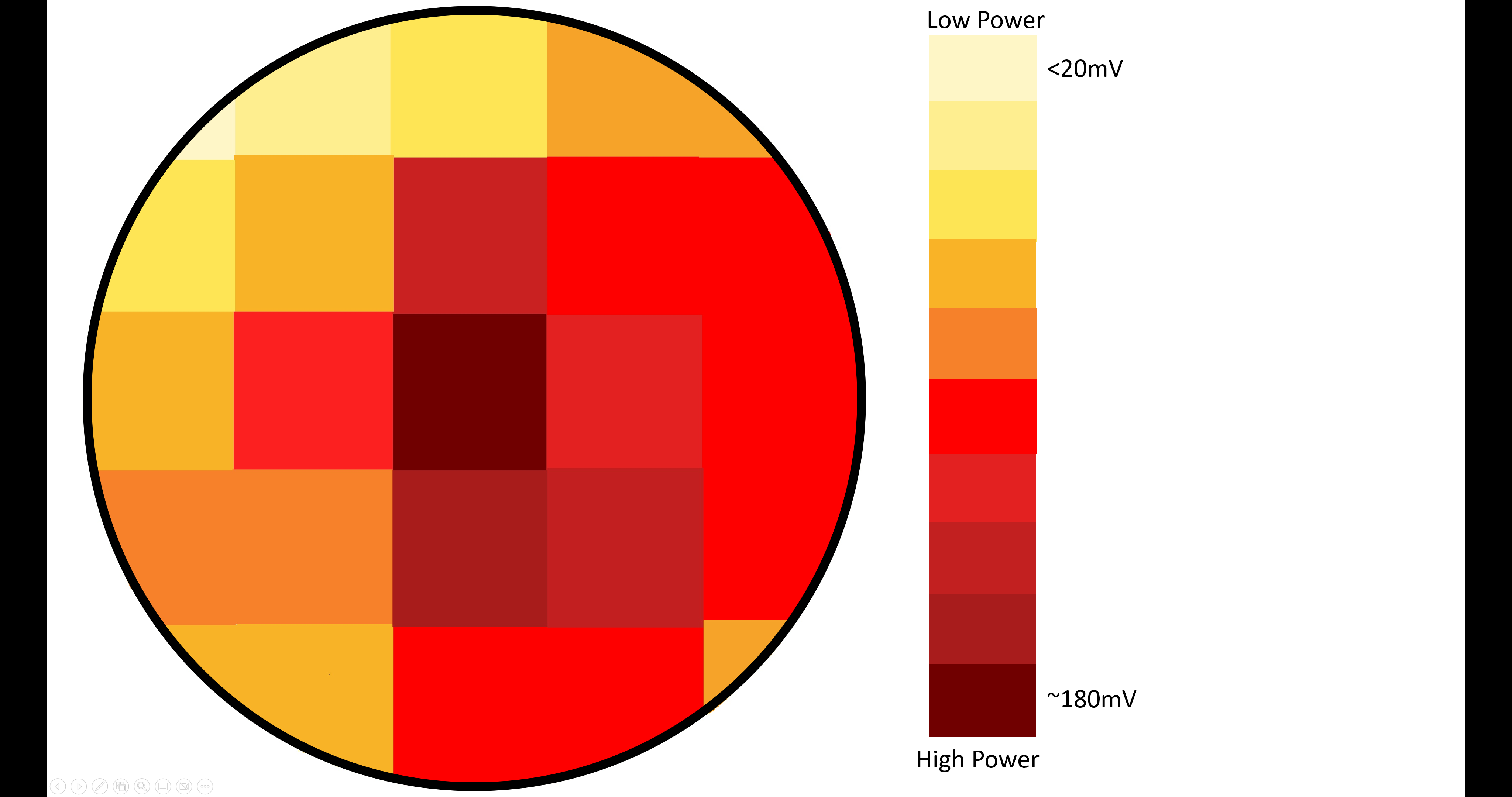


A

B

**Figure S1: Overview of LFU treatment setup and Optimization treatment parameters**

**Figure S1 Low Frequency Apparatus and Optimization Experiments** (A) Schematic of LFU treatment setup for cells and mice. (B), Heat map representing the distribution of LFU output power at the location of treatment, darkest red shows the maximum output power measured by the hydrophone and the yellow color shows the minimum output power. (C), Bar graph showing the growth of Sodium butyrate senescent Vero cells (SCs) in 48h hr after LFU treatments with various power levels at 33 & 39 kHz. Data is represented as the mean of three replicates. (D), Bar graph representing the growth of SCs in 48 h after 33 kHz LFU treatment at the designated power level. Data is represented as the mean ± s.d. of three independent experiments. ** p- value < 0.01 by unpaired two tailed student t-test. (E), Growth was determined as a function of the treatment duration. Control represents no LFU treatment. Results plotted are the mean of three independent experiments ± s.d. *p value <0.05 by unpaired two tailed student t-test. (F)**,** Effect of duty cycle on growth, where control is without LFU. The power level was 4 kPa at 33 kHz for 30’. Results plotted are the means of 3 replicates.


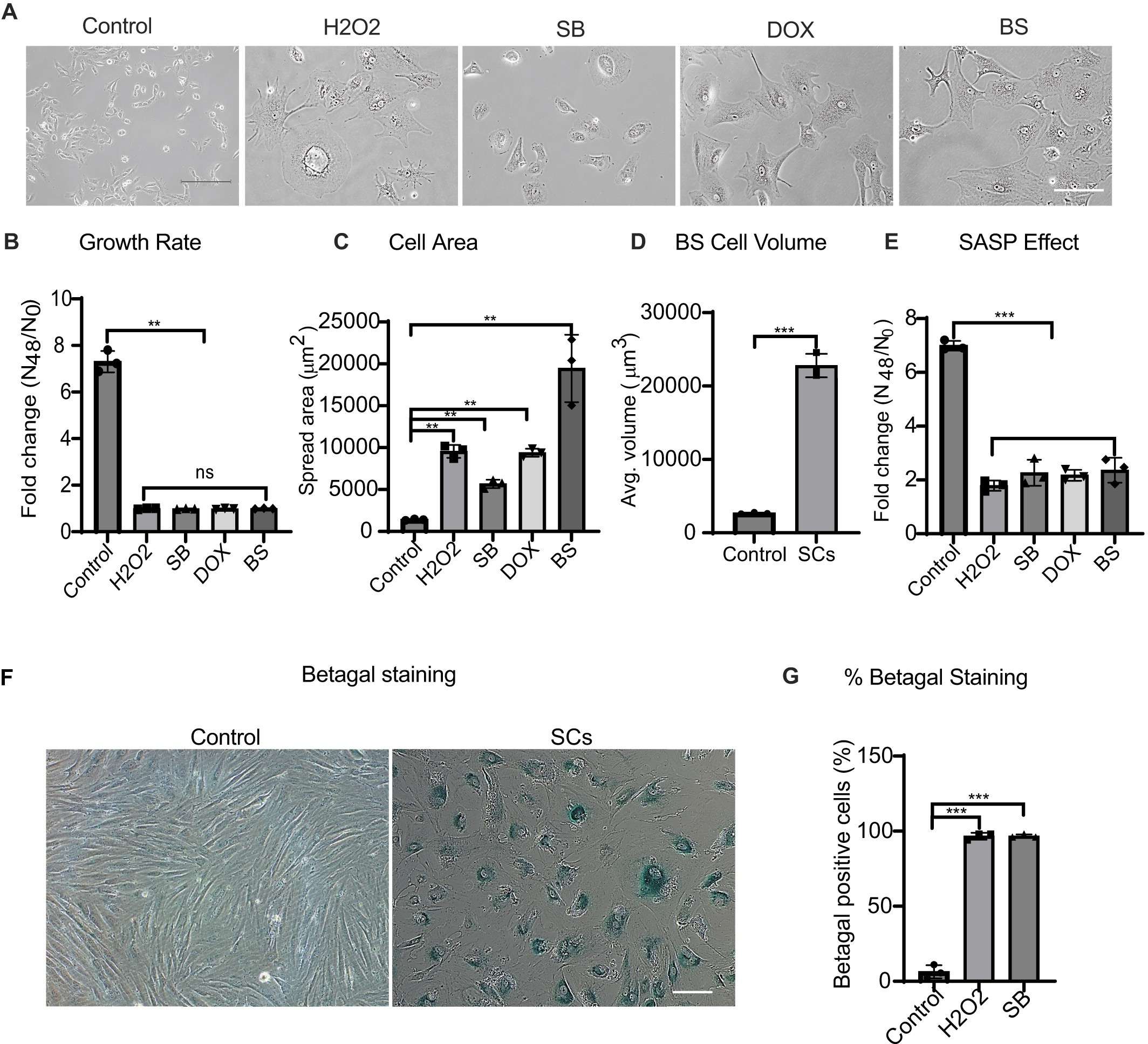
Fig. S2 Senescence Induction and characterization

**Figure S2** **Characterization of Senescent Cells.** (A) Brightfield images of control Vero cells and senescent cells induced by H_2_O_2_, sodium butyrate (SB), doxorubicin (Dox), and bleomycin sulphate (BS). Scale bar=300 µm. (B) Quantification of proliferation shows no cell growth after senescence induction in a 48 h incubation. (C)Senescent cells become enlarged compared to the normal control cells. (D) Quantification of avg. cell volume of bleomycin sulfate treated cells compared to control cells. (E) Supernatant collected from the senescent cells after 24 hour incubation was used to study the growth of normal proliferating Vero cells. Culture medium was used as a positive control. Results are the mean of three experiments ± SD. (F) SA-b-galactosidase staining of control (proliferating) and BS treated senescent cells. Scale bar= 300 µm. (G) Level of b-galactosidase senescence marker in H_2_O_2_ and SB induced SCs. Scale bar=300µm. *P<0.05, **P<0.002, *** P<0.0001; data in (B)-(E) and G are mean ± SD. A minimum of 150 cells were analyzed for spread area, cell volume and β-galactosidase from three independent experiments.

Figure S3: LFU Rejuvenates growth of different types of Senescent Cells without senolysis

BS induced

H_2_O_2_ induced treated

Dox induced treated

A **2.5**

**Fold Change (N_48_/N_0_)**

**2.0**

**1.5**

**1.0**

**0.5**

**0.0**

B **2.0**

1.5


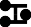


**Fold change (N_48_/N_0_)**

1.0

0.5

0.0

**BS-SCs**

**BS-SCs-LFU**

H2O2-SCs

H2O2-SCs-LFU

C **2.0**

**1.5**


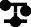


**Fold change (N_48_/N_0_)**

**1.0**

**0.5**

**0.0**

**DOX-SCs**

**DOX-SCs-LFU**

F

# D

E

**Figure S3: LFU activates Growth of various senescent cells.** A) The fold change in the number of Bleomycin Sulphate (BS) (25-33 μM), B) H_2_O_2_ (200 μM), and C) Doxorubicin (500 nM) induced senescent cells treated w/wo LFU after 48 h. Area of D) Bleomycin Sulphate (BS) (33 μM), E) H_2_O_2_ (200 μM), and F) Doxorubicin (500 nM) induced senescent cells treated w/wo LFU after 48 h. ** p value<0.001 and ns p value >0.05.


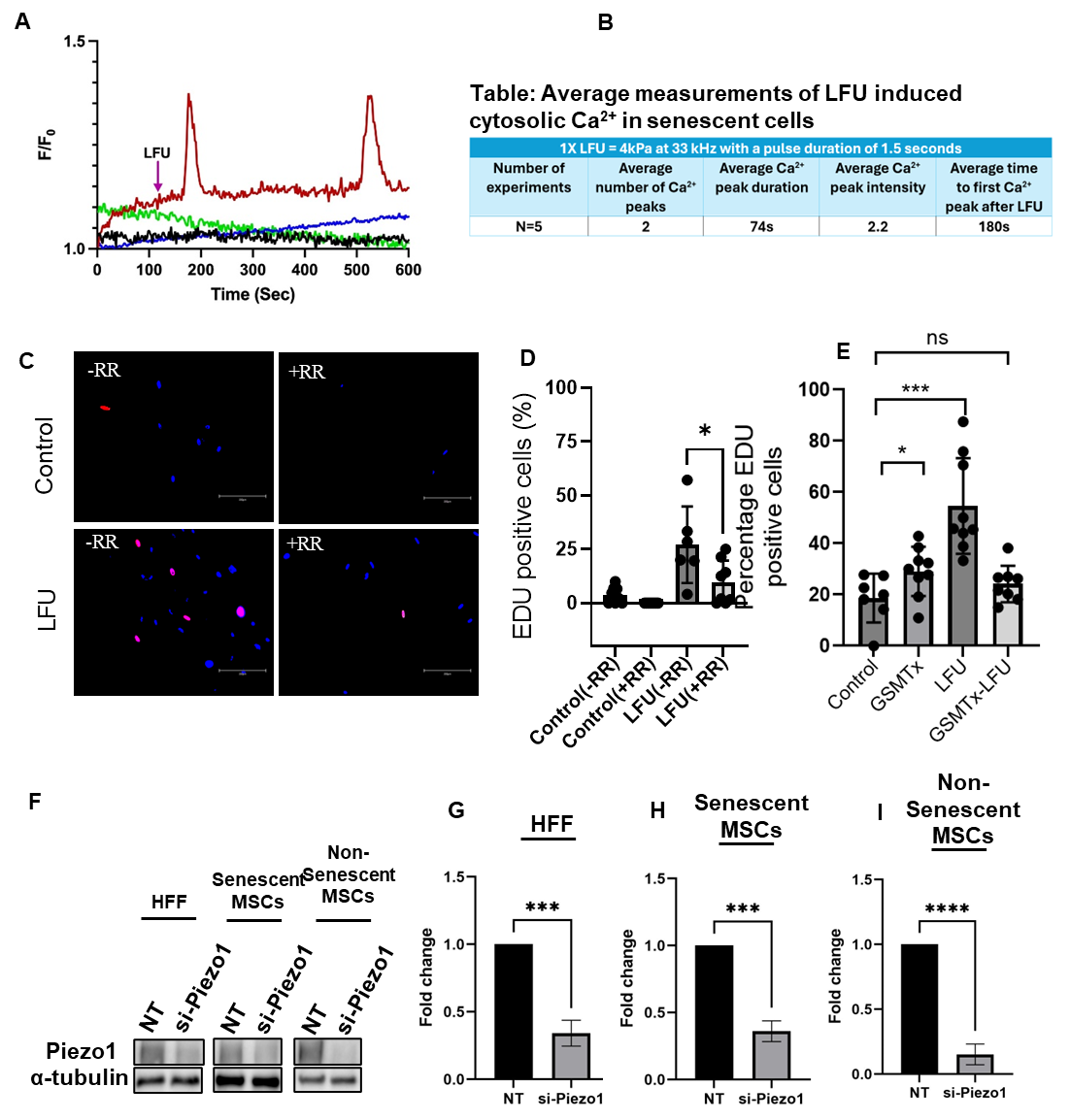


**Figure S4: LFU Stimulates Ca^2+^ entry and Inhibition of Piezo 1 and TRPV1 channels blocks rejuvenation**. (A) Time course of Ca^2+^ levels in non-senescent (P3 HFFs, black line) and senescent cells (P21 HFFs, red line) treated with Ca^2+^ channel inhibitors, GsMTx4 (green line) or Ruthenium Red (RR, blue line). (B) Table summarizing the average measurements of LFU induced cytosolic Ca^2^**^+^** waves in senescent cells. (C) Representative Immunofluorescence-stained images of 4 day old bleomycin-sulfate-induced senescent cells. Cells were treated with or without LFU in presence or absence of Ruthenium Red (RR). Scale bar= 300 μm. Cells were then incubated for 12 h in EdU reagents and stained for EdU (red staining) as well as nuclei (DAPI stained, blue). (D), quantification of proliferating cells by manually counting the red colored nuclei. EdU positive cells, and dividing by the total number of cells in the fields. (E) Quantification of cell proliferation of passage 15 HFF cells with or without piezo1 inhibitor, GSMTx4. Results are plotted as the mean of three independent experiments ± s.d. * p value < 0.05 using unpaired two tailed test, more than 100 cells were counted in each condition. F) Representative blots showing the knockdown of Piezo1 in HFFs, senescent MSCs and non-senescent MSCs. α-tubulin serves as loading control. (G-I) Quantification of the western blots shown in (F). Piezo1 knockdown is partial in most senescent HFFs and senescent MSCs, however, around 90% knockdown efficiency is achieved in non-senescent MSCs. Results are shown as mean ± SD. **** p value <0.001 and **** p value < 0.0001*. A t-test was used to obtain the results.

Figure S5:


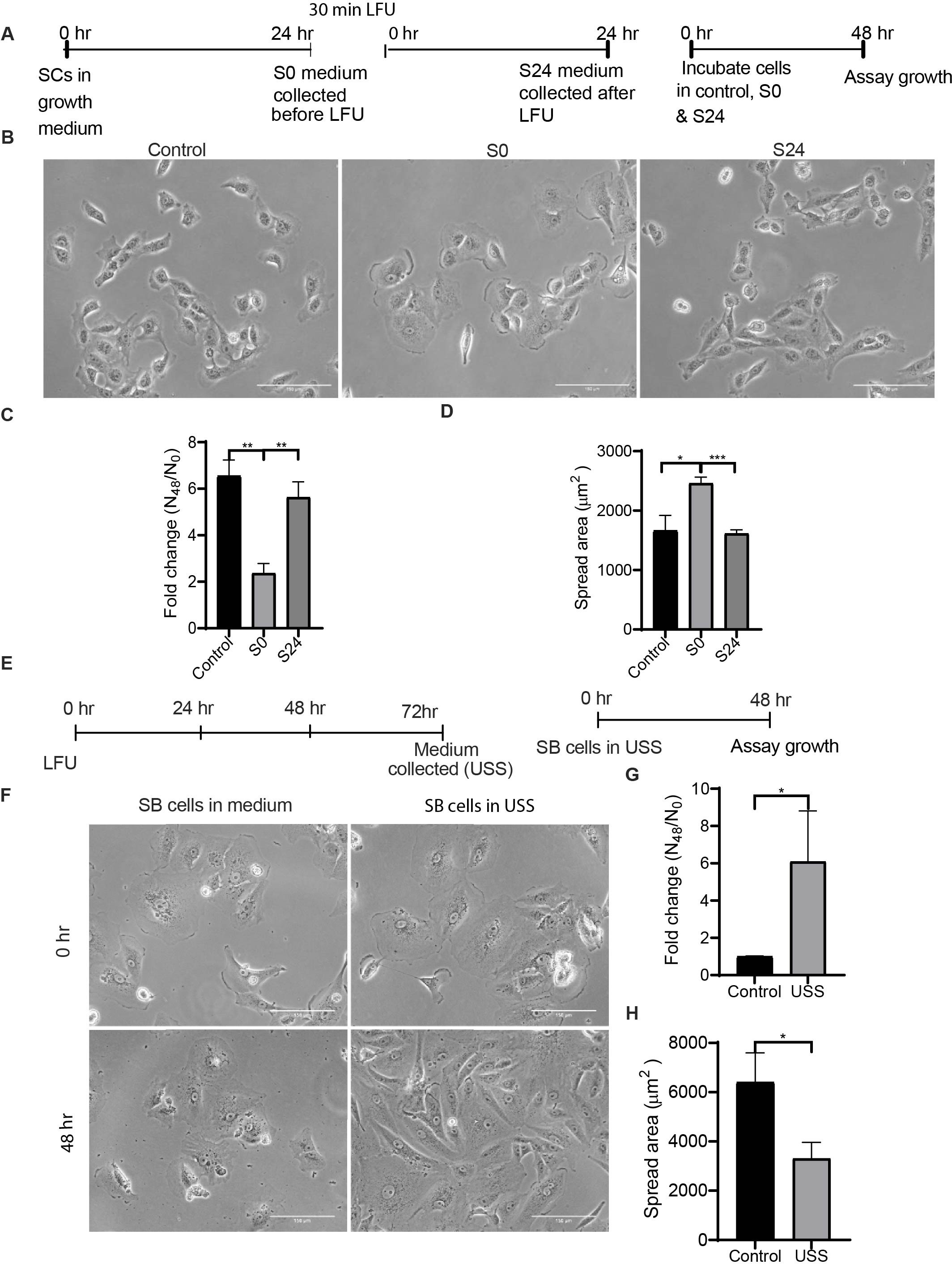

**Figure S5: LFU Treatment Decreases SASP Secretions.** (A) Schematic illustration of the experiment where BS senescent cells were cultured in growth medium for 24 hr and then they were treated with low frequency ultrasound (LFU) for 30 min. Supernatant was collected after the LFU treatment (S0) and cells were incubated for another 24 hour in fresh medium before supernatant was collected (S24). To check the effect of LFU treatment, supernatants S0 and S24 were used to check the growth of non-senescent HFF cells. (B) Representative brightfield images of normal vero cells after 48 hours of incubation in control growth, S0, or S24 medium. (C) Quantification of normal vero cell numbers after 48 in control growth, S0 or S24 medium. (D) cell areas after 48 in control growth, S0 or S24 medium. (E) Chemokines and cytokines in supernatants from untreated and LFU treated late passage HFF (P19) cells after 24 hours incubation were measured using Multiplex assay. Results are plotted as mean± s.d., n= 6 replicates, ns not significance, * p value <0.05 ** p value <0.01, *** p value 0.0001, and **** p value 0.00001 using Mann Whitney Test.

**Figure S6: LFU Rejuvenation Requires Sirtuin 1 Activation of Autophagy**

# **B**

A

LFU

Control


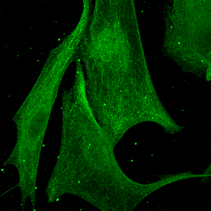

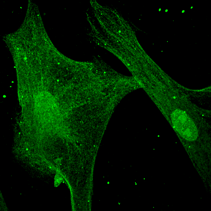


Sirt1


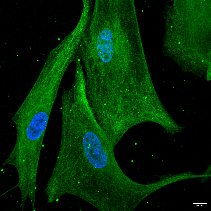

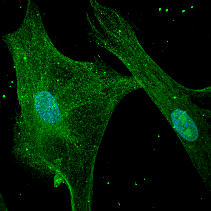


Merged

Scale bar= 10 μm


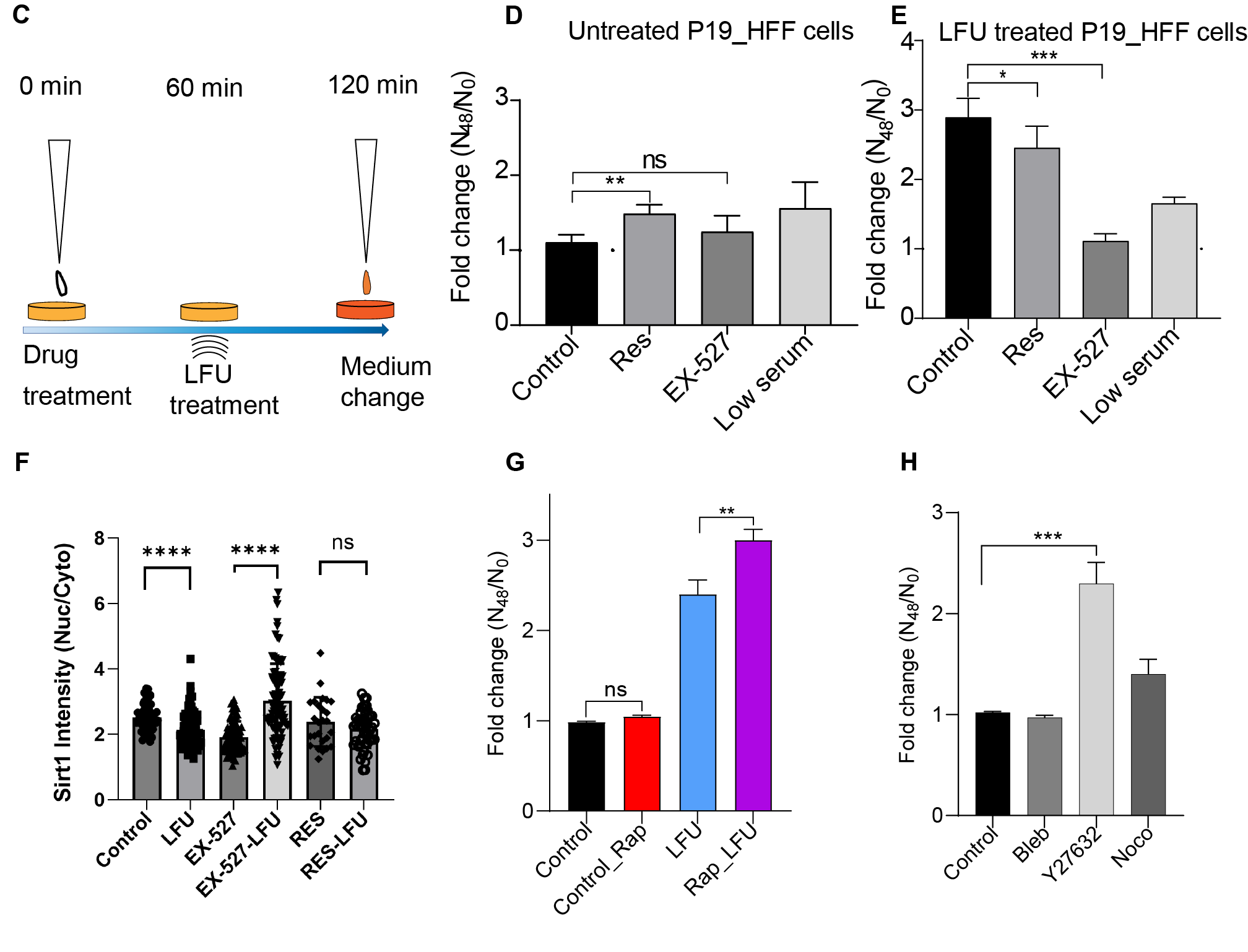


Figure S6: Sirtuin 1 Distribution and Function in Rejuvenation of Growth and Autophagy. (A) Distribution of Sirtuin 1 antibody and DAPI in senescent and LFU rejuvenated cells. (B) Ratio of GFP/RFP in BS cells transfected with GFP-LC3-RFP after treatment with nothing (Control), 10 mM Chloroquine diphosphate (CCD), 10 mM of EX-527 (EX), LFU for 30’, or EX-527 plus LFU (LFU-EX). (C) Schematic of Resveratrol and EX-527 treatment, followed by Ultrasound treatment and medium change. (D) Growth after 48 h of untreated control senescent P19_HFF cells in presence of 10 mM resveratrol (Res), 10 mM of EX-527 (EX-527), or low serum (1% serum). (E) growth after 48 h of LFU treated P19_HFF cells in the presence of the same drugs. (F) quantification of Sirtuin 1 expression by intensity ratio of Sirtuin 1 antibody fluorescence to DAPI fluorescence after no treatment (Control), LFU treatment for 30’ (LFU), 10 mM of EX-527 treatment (EX-527), EX-527 and LFU treatment (EX-527_LFU), Resveratrol treatment (RES), and Resveratrol plus LFU treatment. (G) Growth rate after 48 h of P19 HFF (Control), treated with Rapamycin (1 mM) (Control_RAP), treated with LFU (LFU) or treated with Rapamycin plus LFU (RAP_LFU) shows significantly increased growth with rapamycin and LFU treated SCs compared to with and without rapamycin controls. (H) Growth of P19 HFF SCs in presence of cytoskeleton inhibitors including Blebbistatin (10 mM), Y27632 (1 mM), and Nocodazole (1 mM).

Figure S7: LFU reverses p53, **γ**h2ax, H3K9me3, ROS and MitoSox levels in senescent cells

Control LFU

## A B


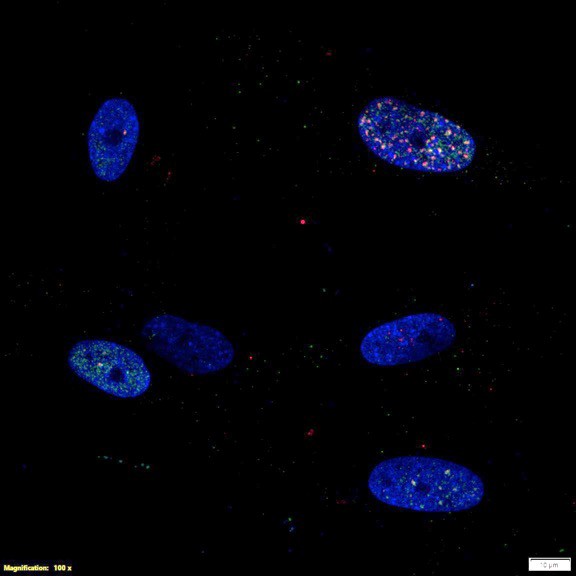

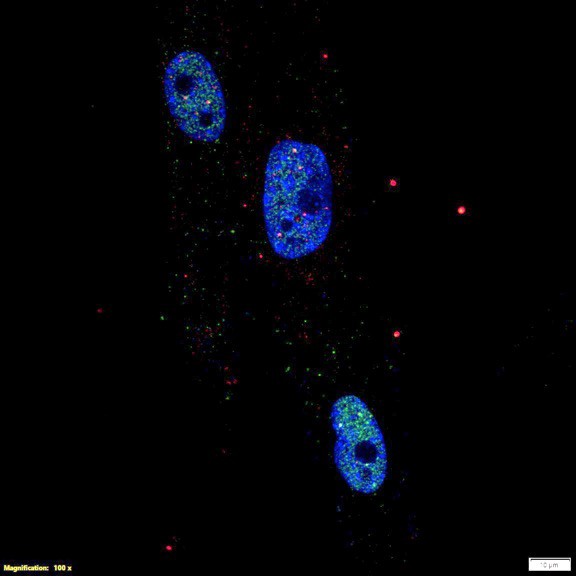

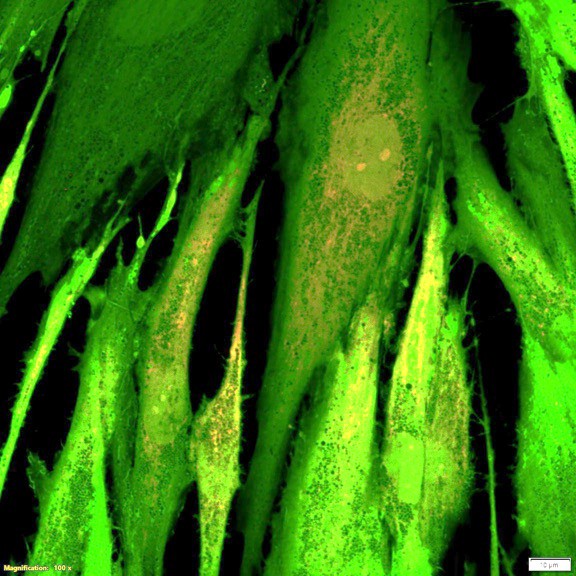

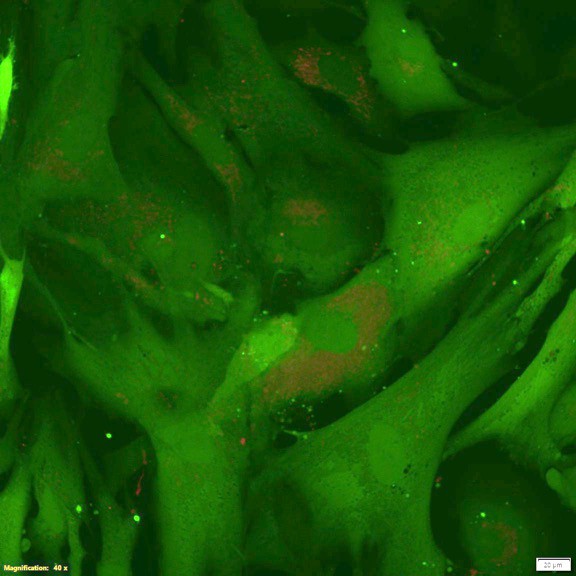

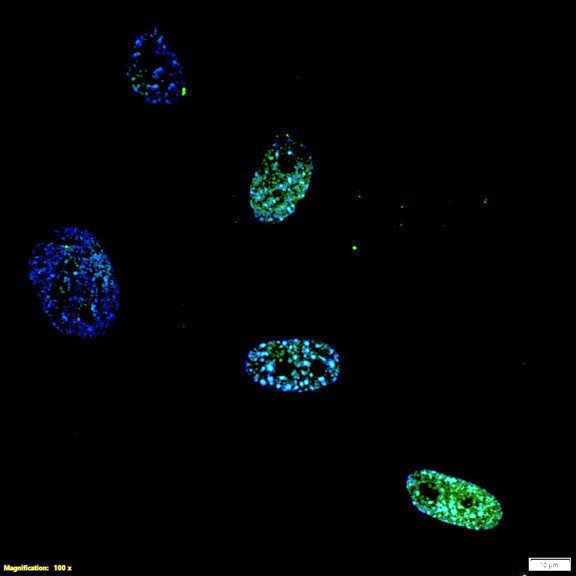

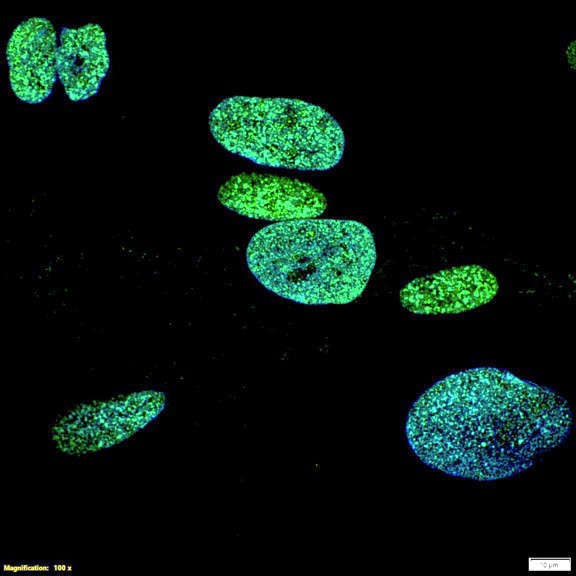

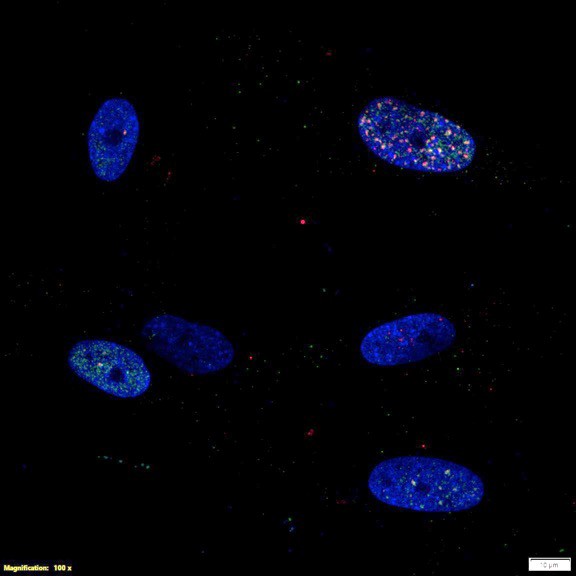


P53

γH2AX

H3k9me3

ROS

MitoSOX

D

✱


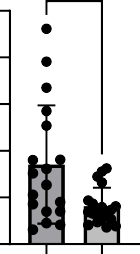
**50**

**Mean p53 Intensity (a.u.)**

**40**

**30**

**20**

**10**

**0**

**Control LFU**

## E

**Mean H3k9me3 intensity (a.u.)**

## C

**200**

**150**

✱✱✱✱

**15** ✱✱


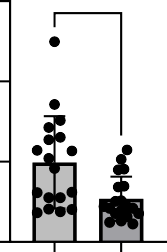
**10**

Mean γH2AX intensity (a.u.)

**5**

**0**


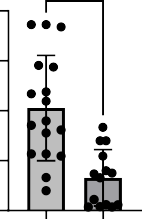
**Control LFU**

**100**

**50**

✱✱


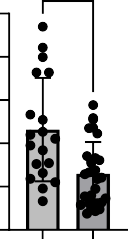
F G **250**

**Intensity of ROS (a.u.)**

**200**

**150**

**100**

**0**

**Control LFU**

## H

**Mean Inesnity of MitoSox Red (a.u.)**

**150**

**100**


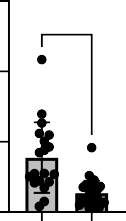


✱✱✱✱

**50**

#### ****I


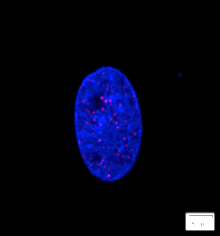

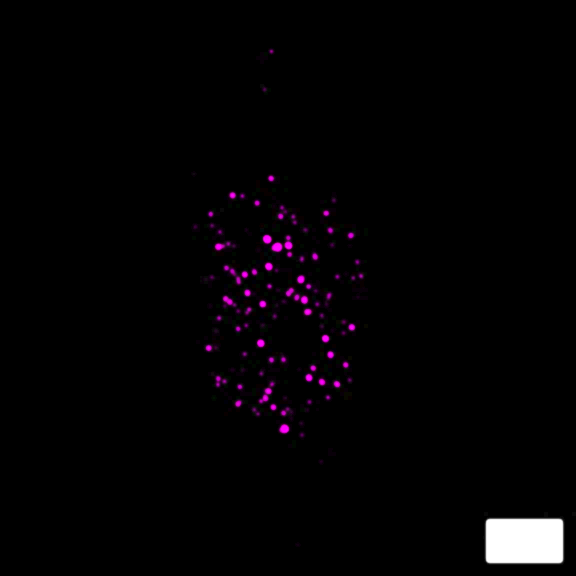

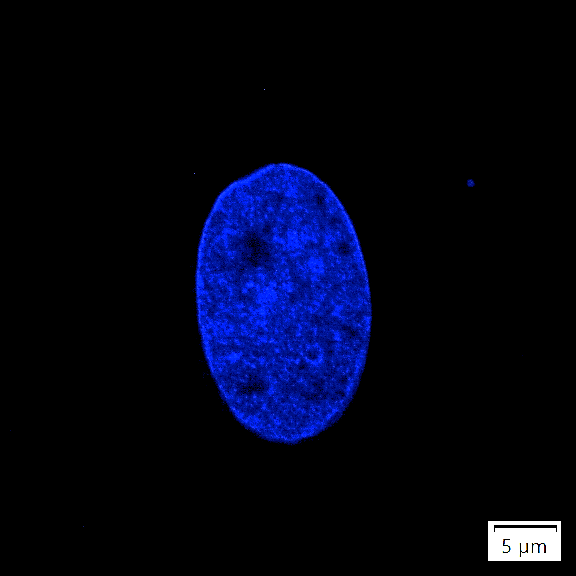

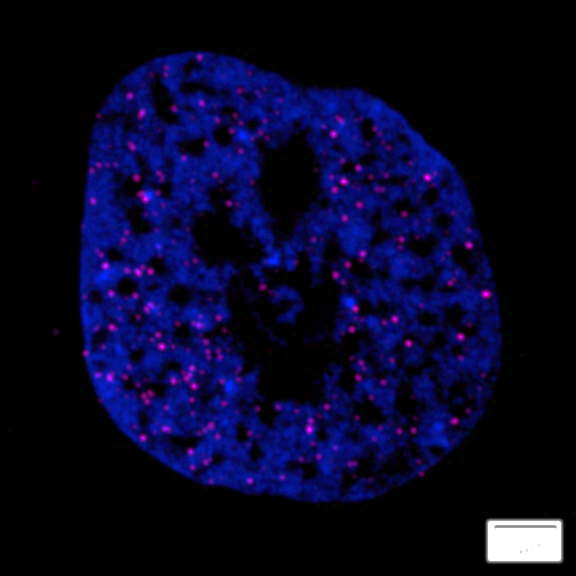

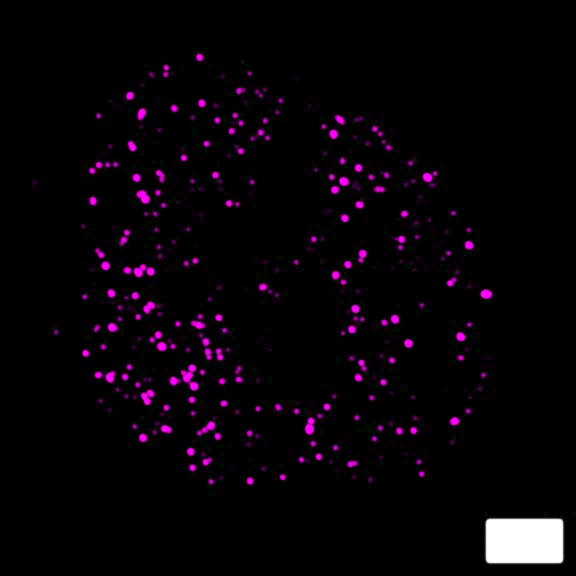

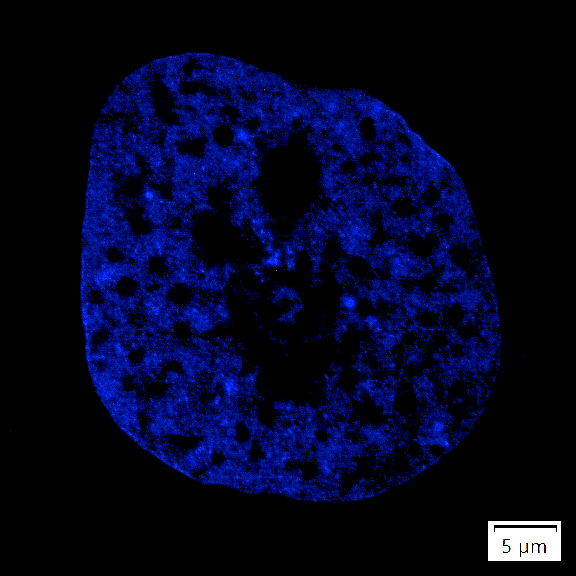


K

L

DAPI

5mC

Merged

**50**

**0**

**Control LFU**

## J

**0**

**Control LFU**

**Figure S7. Senescence Markers Are Reduced, Telomeres and 5mC Are increased after LFU Treatment**. (A) Representative images of p53 (green) and gH2AX foci (red) stained cells. Sodium Butyrate senescent Vero cells were treated two times with or without LFU and then immunostained with P53 and gH2AX antibodies. Staining was done 24 hours after LFU treatment (2^nd^ treatment that was 1 day after first). (B) Quantification of p53 (green) intensity from the three replicates. (C) Quantification of gH2AX mean intensity from three replicates. (D) Representative images of H3k9me3(green) stained cells Sodium Butyrate senescent Vero cells were treated two times with or without LFU and then stained with H3k9me3 antibody (one day separated first and second LFU treatments and staining). (E) Quantification of H3k9me3 (green) intensity from the three replicates using standard conditions. (F) Representative images of ROS (green) and MitoSOX (red) treated cells with live cell staining probe. Sodium Butyrate induced Vero cells were treated with or without LFU and images were captured after 24 h. (G) Intensity of ROS (green) staining and (H) MitoSOX (red) staining from the three replicate experiments. (I) Telomere length measurements of HFFs after passage 2 (P2) and passage 22 (P22) shows shortening but length increases with LFU (P22_LFU) n=4, N=1. Results are shown as mean ± SD.  (J) In mesenchymal stem cells, the telomere length decreases slightly from passage 3 (P3) to passage 19 (P19_C) but is increased proportionally by LFU (P19_LFU), n=6, N=1. (K) Fluorescence images of control P18 (upper images) and LFU treated P18 HFF cells stained with DAPI and anti-5mC antibody. (L) Quantification of intensity of 5mC fluorescence in nuclei of control and LFU treated P18 HFF cells. Scale bar =10 μm. Data is plotted as mean± s.d. from the three replicates. One data point represents one cell analyzed. Non-parametric Mann Whitney test was used to determine the statistical significance between two groups. * p value<0.05, ** p value <0.007, **** p value<0.0001, and ns p value >0.05.


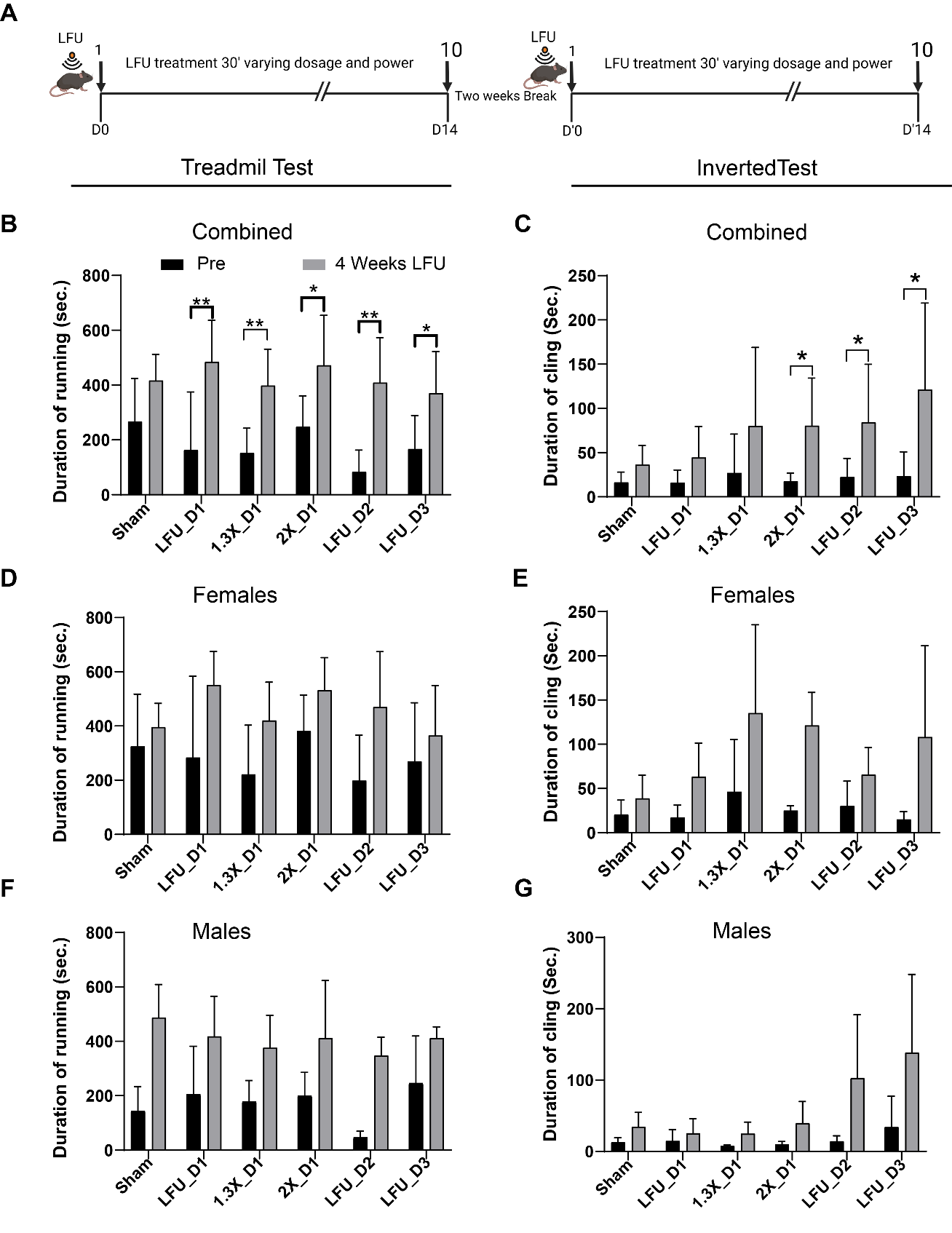


**Figure S8 Effects of LFU dosage on performance of aged mice.** (A) Schematic illustration of the treatment plan. Mice (20-24 months old mice (C57BL/6J strain)) were treated 30 min at 1X power every day (D1), every other day (D2) and every third day (D3), 1.3x power every day (1.3X_D1) or 2X power every day (2X_D1) for two weeks followed by a two week break and then two-weeks of treatment. Performance of treated mice was assessed as pre and post treatment with standard treadmill and cling tests. Each study group contained five male and five female mice. After four weeks of LFU treatment with 2 weeks break, both the treadmill test (B) and inverted cling test (C) showed significant improvements in performance in the LFU groups. The female mice showed similar relative increases in the treadmill (D) and the Inverted cling test performances of females (E) to the male treadmill (F) and inverted cling test (G). Three of fine cohorts of the males showed overall low performance in the inverted cling tests but increases with LFU, which is confusing. Results are plotted as mean ± SD. Student t-test was used to determine the statistical significance. Statistical significance was given in p-value. *P<0.05, **P<0.002, *** P<0.0001.


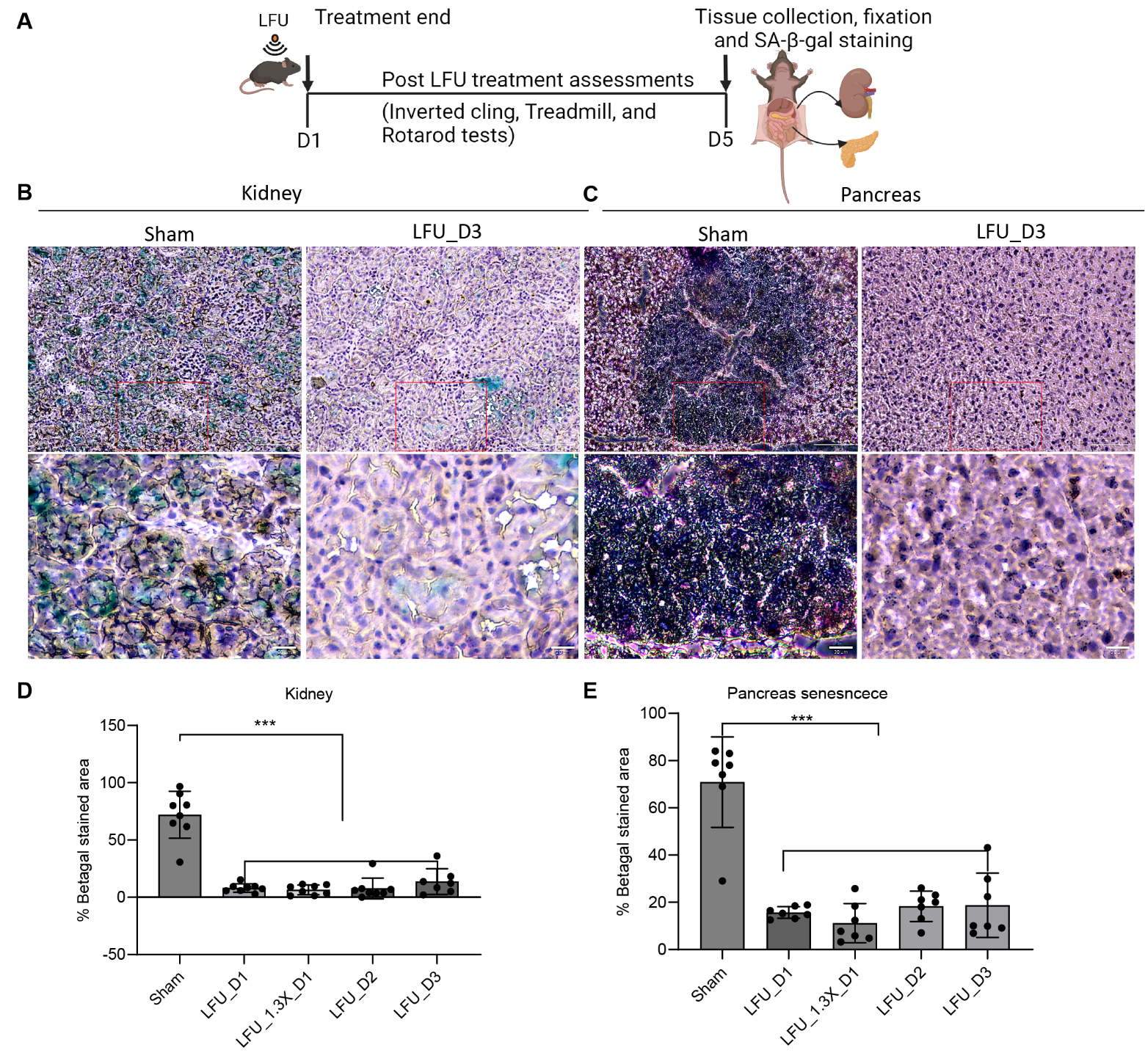


**Figure S9 LFU decreases fraction of SA-β galactosidase staining cells in kidney and pancreas.** (A) Schematic illustration of the treatment plan and tissue staining. 22 month old mice were treated 30 min every day with 1X power (LFU_D1) or 1.3X power, (LFU_1.3X_D1), every other day (LFU_D2) and every third day (LFU_ D3) day with 1X power for two weeks then two weeks break followed by a second two-week treatment. Each study group contained five male and five female mice. After 4 weeks of treatment followed by physical assessment, mice were euthanized, and kidney and pancreas were collected for SA-β galactosidase staining. (B) SA-β-galactosidase-stained kidney sections of sham and LFU treated mice. (C) SA-β-galactosidase-stained pancreas sections of sham and LFU treated mice. Blue color indicates b-galactosidase activity, scale bar=150 µm and 20 µm. (D) Quantification of b-galactosidase staining of kidney sections in sham and LFU treated mice. At least 10 images from each mouse’s kidney sections for the two mice in each category were analyzed.  (E) Quantification of b-galactosidase staining of pancreas sections in sham and LFU treated mice. At least 10 images from each mouse’s kidney sections for the two mice in each category were analyzed. Results are plotted as mean ± S.D. Two tailed unpaired Student t-test was used to determine the statistical significance. P-value greater than 0.05 is represented by ns. Statistical significance was given in p-value. *P<0.05, **P<0.002, *** P<0.0001.


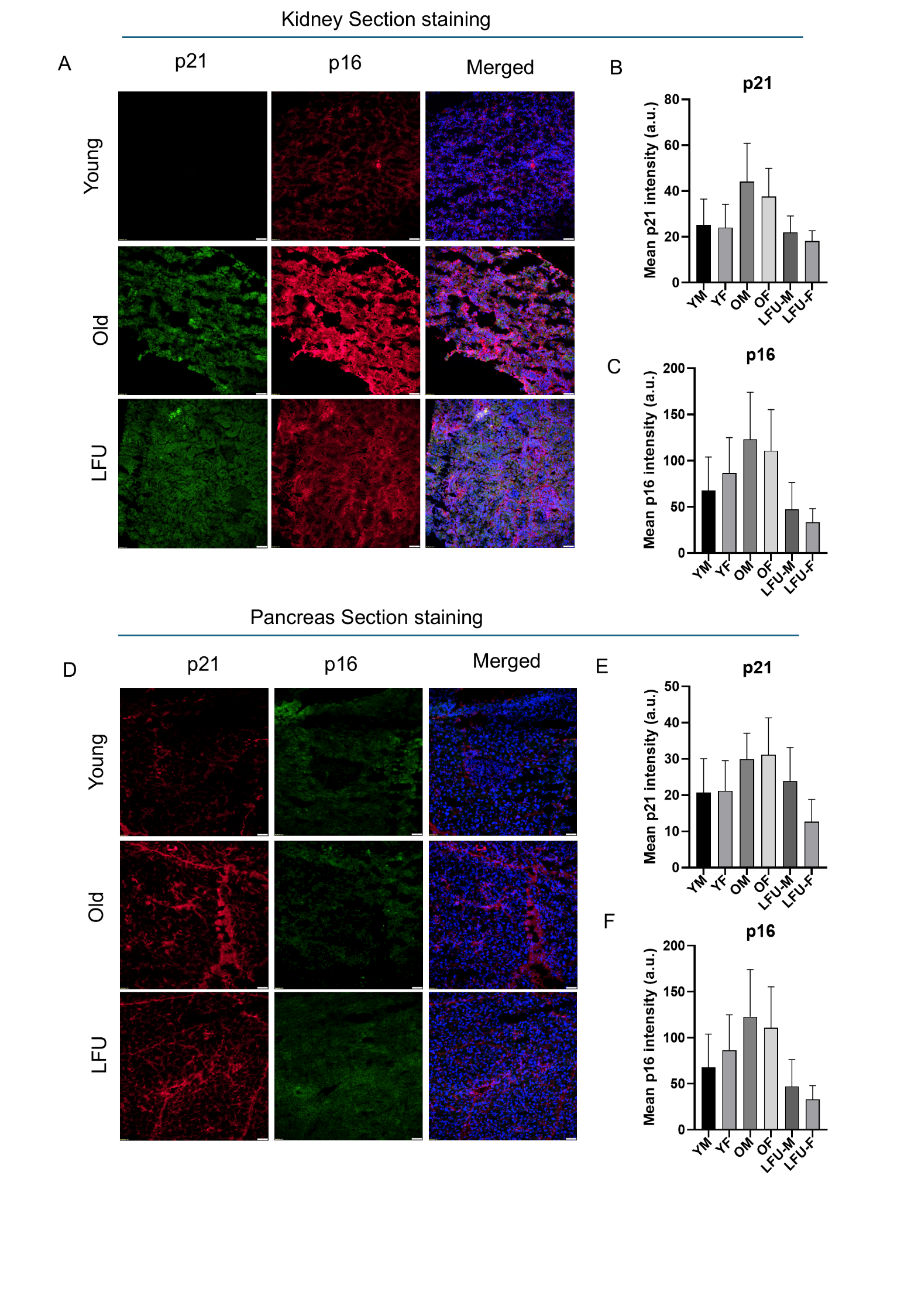


**Figure S10: LFU reduces p16 and p21 staining in Kidney and Pancreas. 22-month-old mice** were treated with LFU for two months (10 treatments in the first and third months) and were euthanized 5 days after the last LFU treatment. In addition, two untreated young mice (8 weeks of age) were euthanized. Organs were quick frozen and then frozen sectioned. Tissue sections of the pancreas and kidney were freeze substituted and stained for the senescent cell markers, p16 and p21. (A) Representative fluorescence images of p16 and p21 immunostaining in a kidney section. Scale bar= 10 μm. (B) Representative fluorescence images of p16 and p21 immunostaining in a pancreas section. Scale bar= 10 μm. (C) Quantification of p16 and p21 staining per area in kidney section, and (D) Quantification of p16 and p21 staining per area in Pancreas. Results are shown as mean ± SD. *p value<0.05, **p value<0.001, and *** p value<0.0001. YM-young male, YF-young female, OM-old male, OF-old female, LFU-M treated old male, and LFU-F treated female.


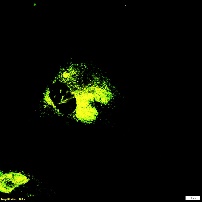

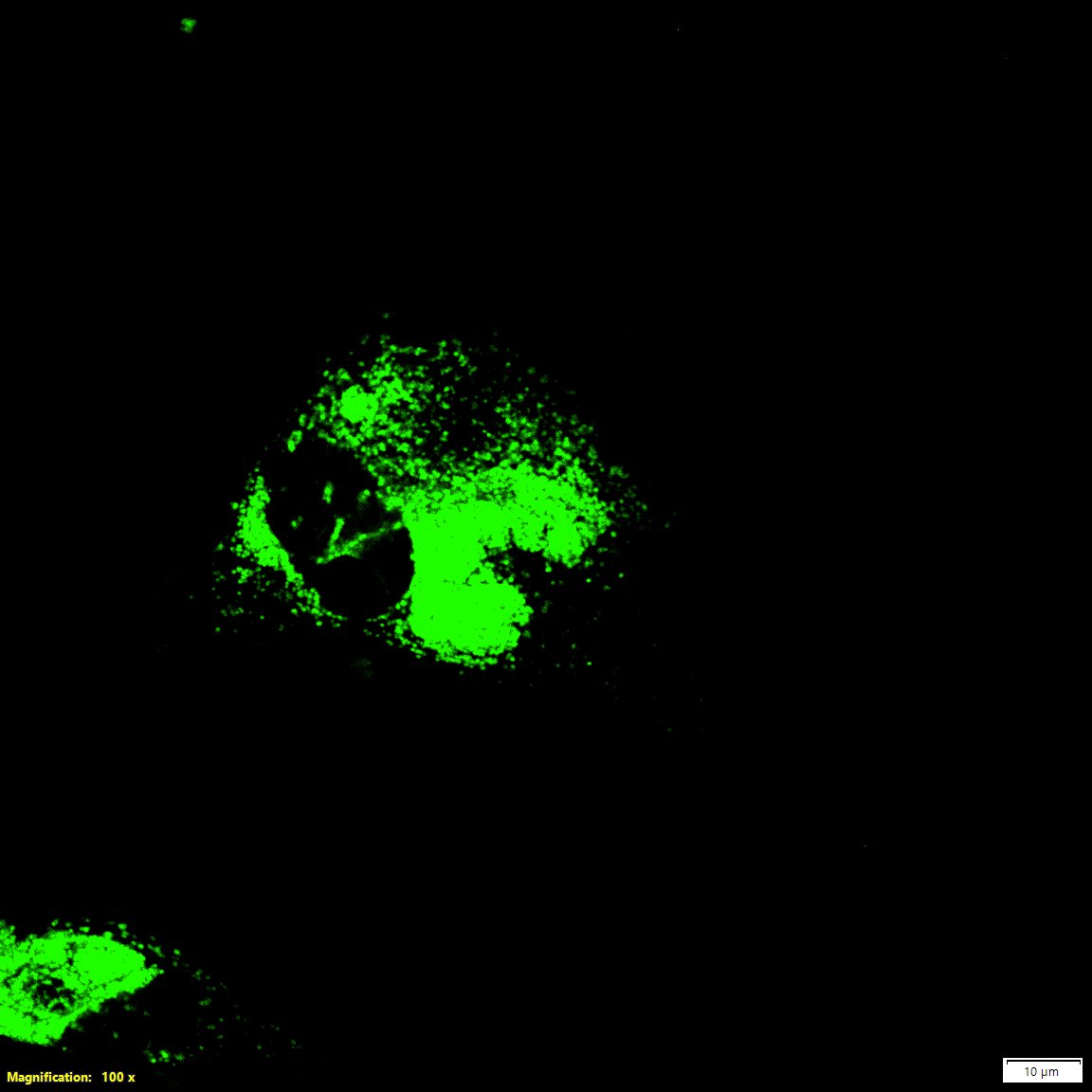

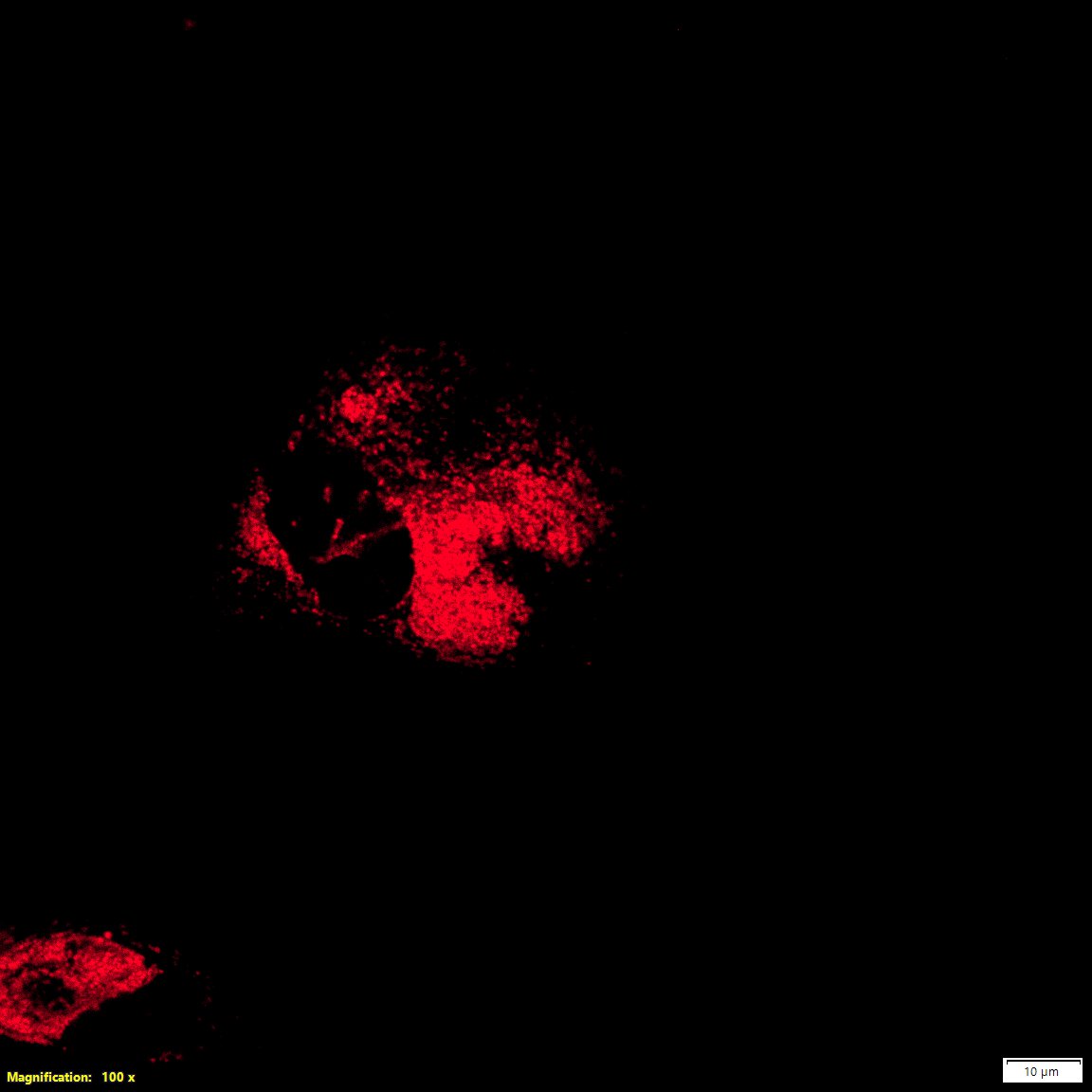

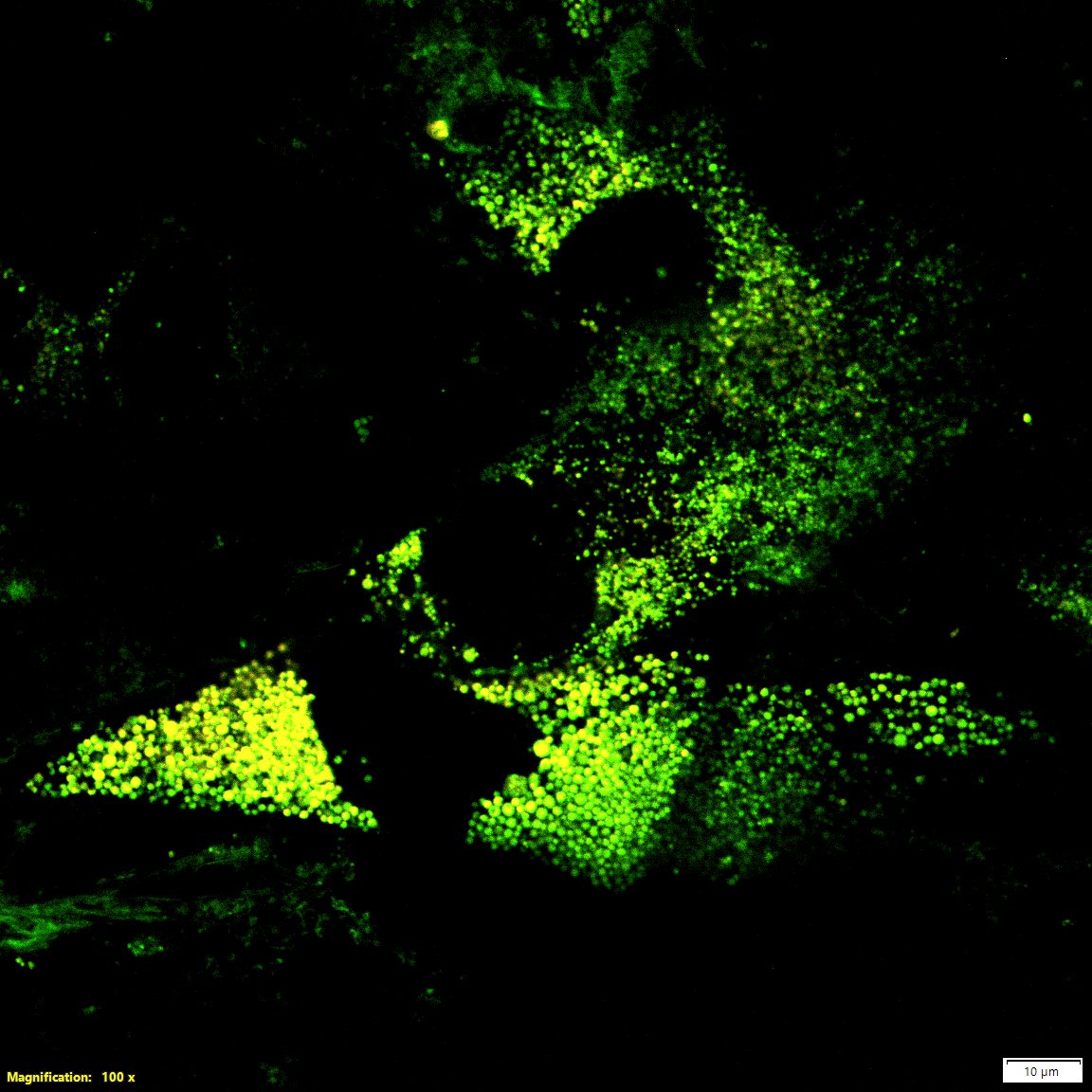

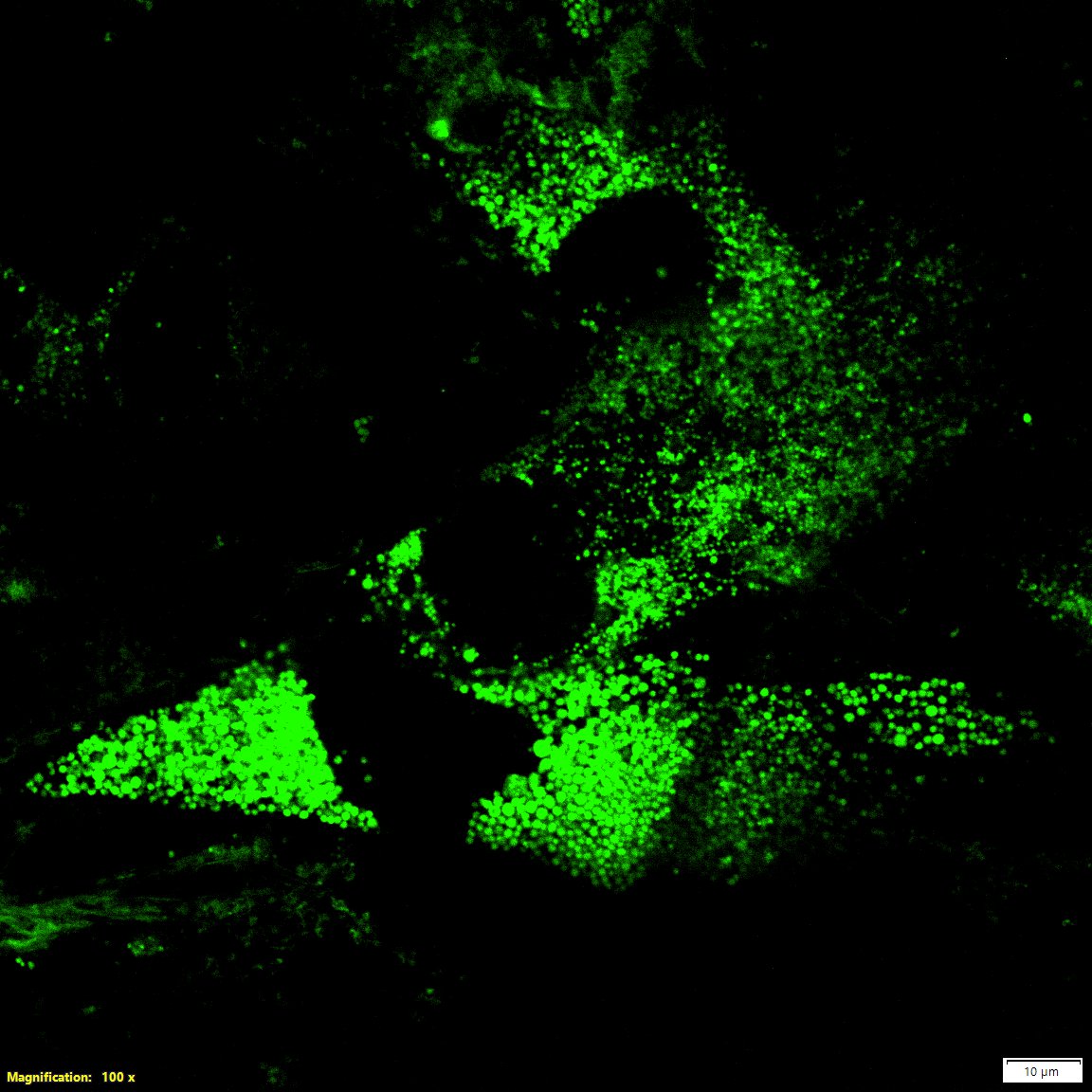

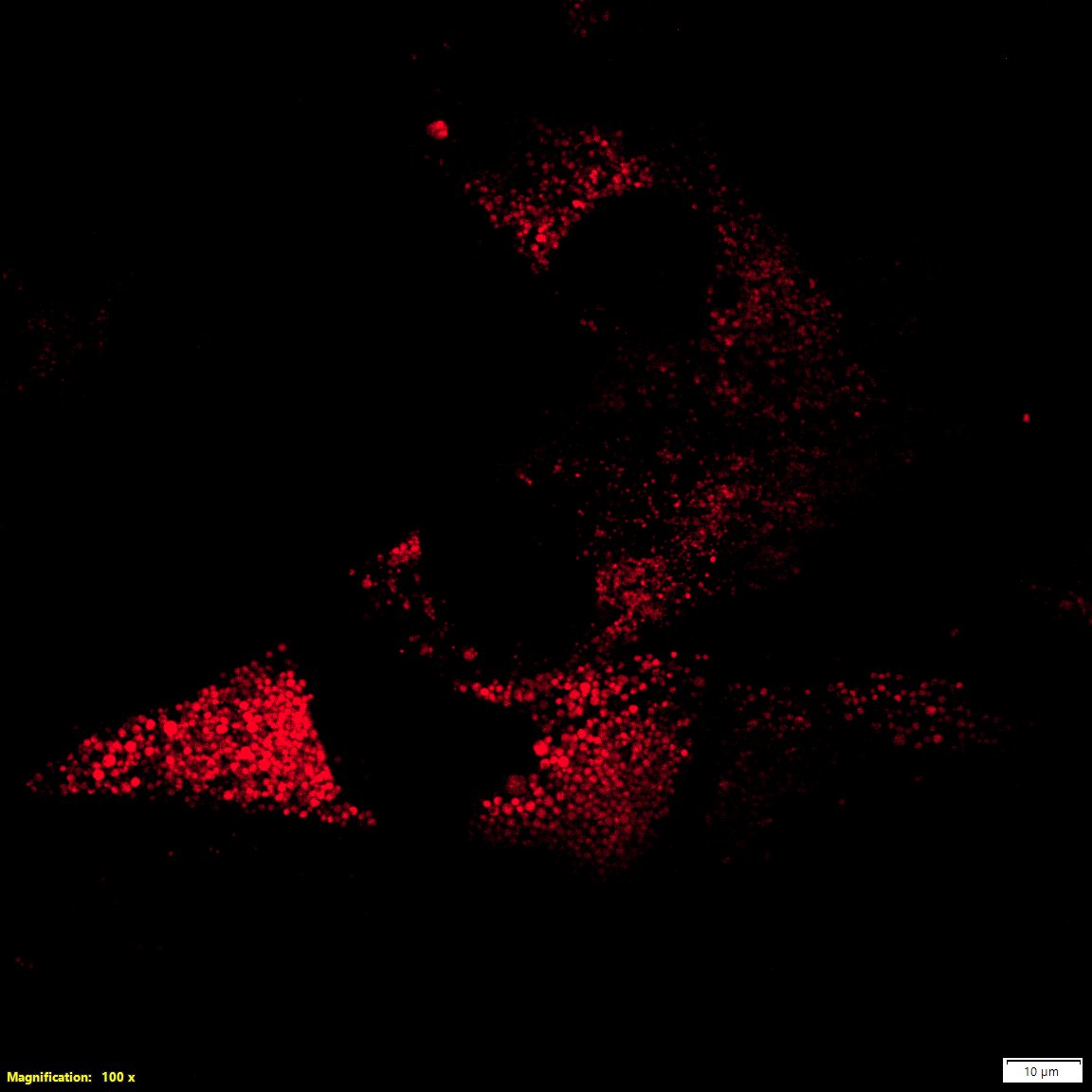

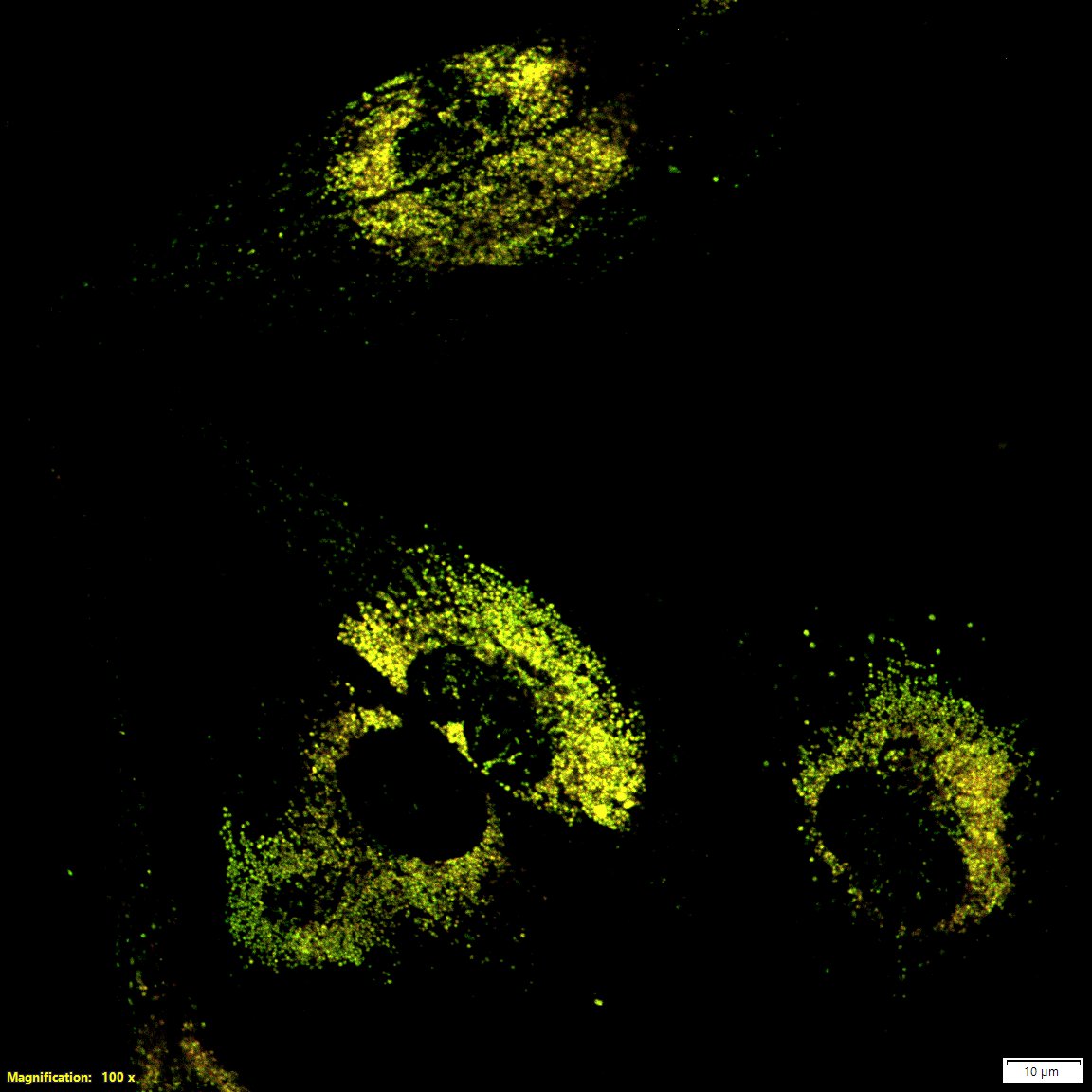

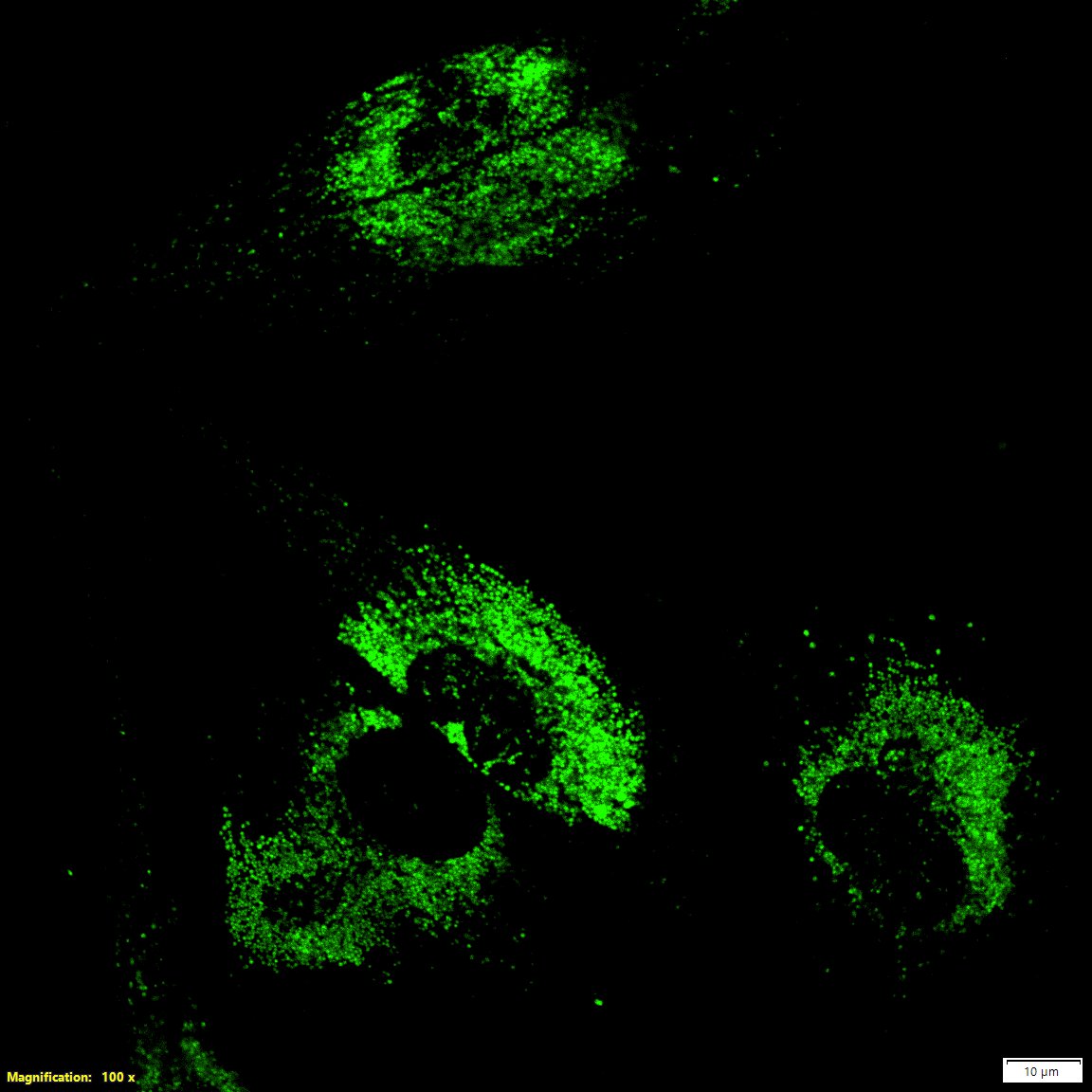

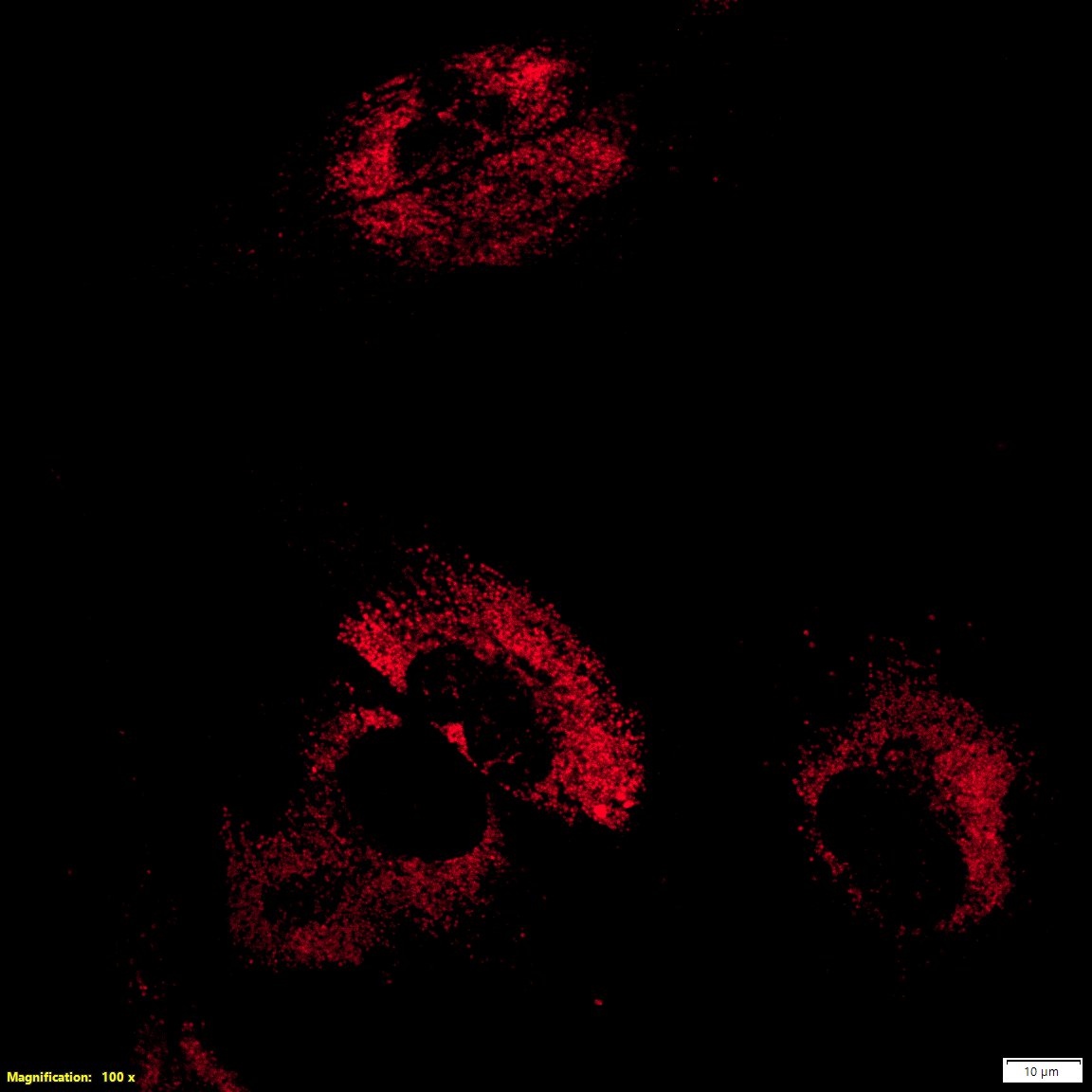

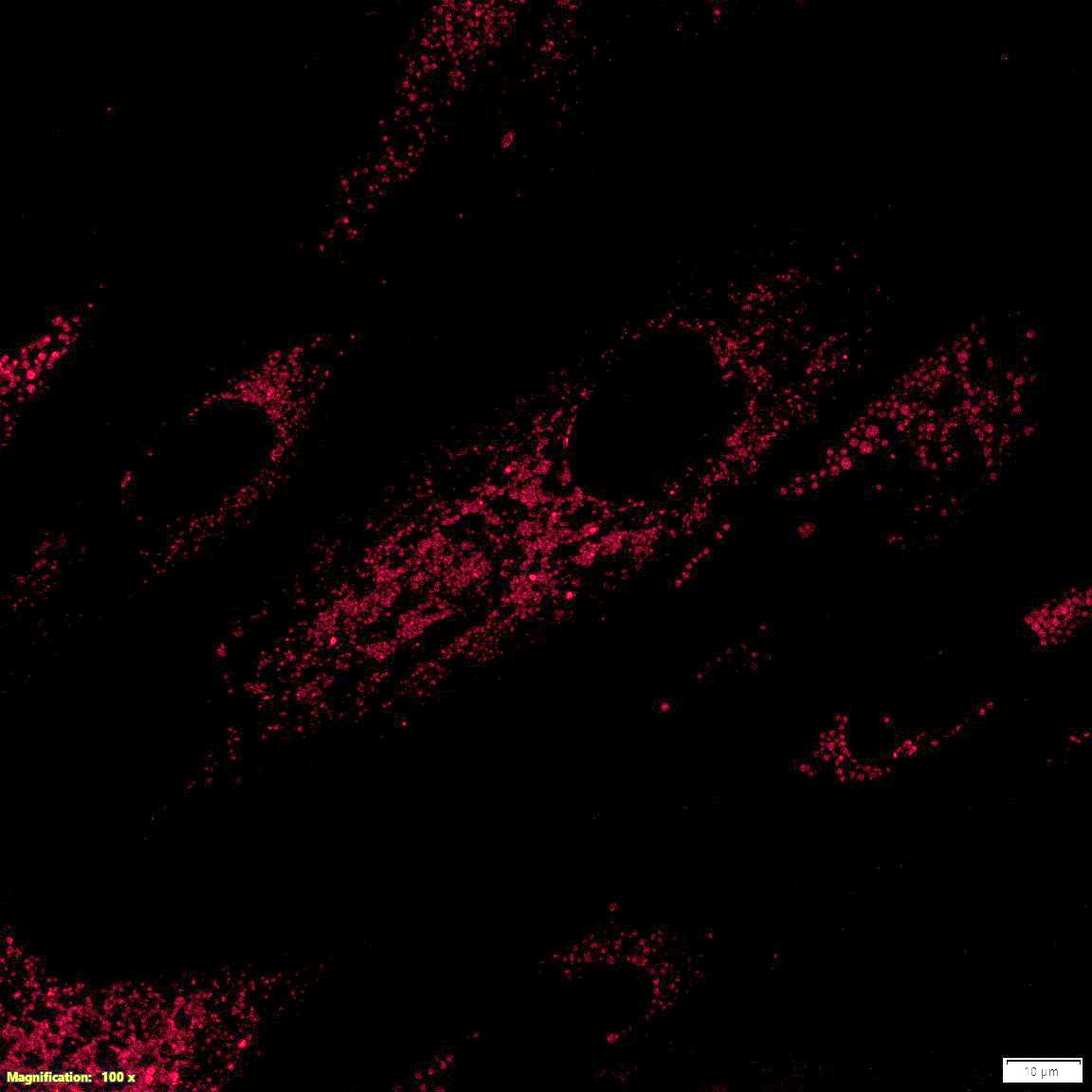

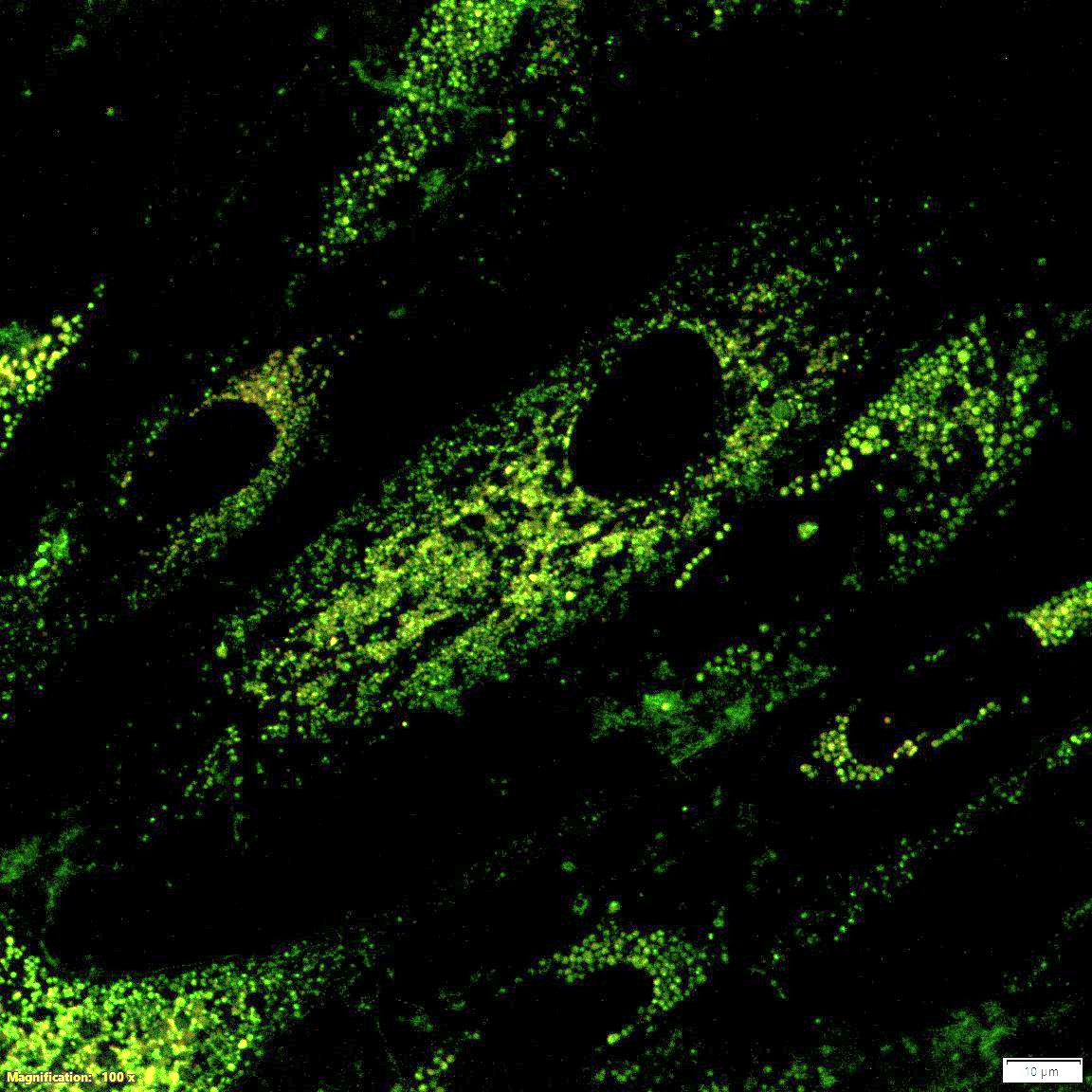

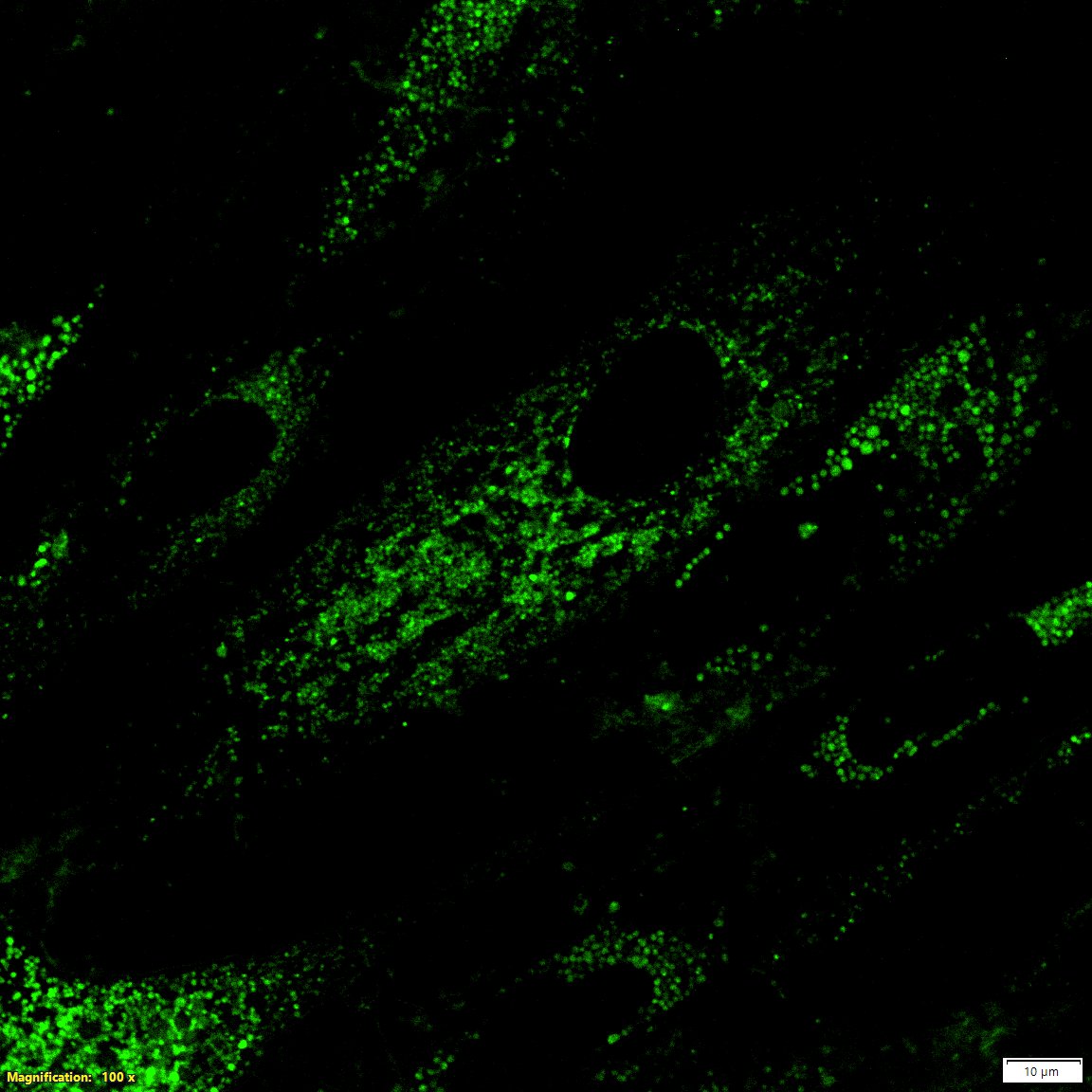


LC3-RFP

LC3-GFP

Merge

**B**

**A**

Control

LFU

CCD

CCD-LFU

A

**Fig. S11 LFU Treatment Increases Autophagy and Decreases mTORC1 Activity** (A) Representative immunofluorescence images of GFP-LC3-RFP transfected in P18 HFF cells treated with LFU with or without Chloroquine diphosphate (CCD, 10 Um). Control cells were not treated with CCD or LFU. Cells were transfected with autophagy sensor, GFP-LC3-RFP and incubated overnight as per manufacturer’s instructions (See the methods sections for details). Cells were then treated with CCD or LFU and incubated for 24 h. Confocal images were captured after 24 h incubation. Scale bar= 10 μm. (B), violin plots show the quantification of relative intensity of GFP to RFP fluorescence. Autophagy flux is inversely correlated with GFP relative intensity because active autophagy quenches the GFP fluorescence. Result is plotted as mean± s.d., n=80 fields ROI and a minimum of 10 cells were analyzed. * p- value <0.05, ** p-value <0.01 and **** p value<0.0001, two tailed Mann Whitney test was used.

**
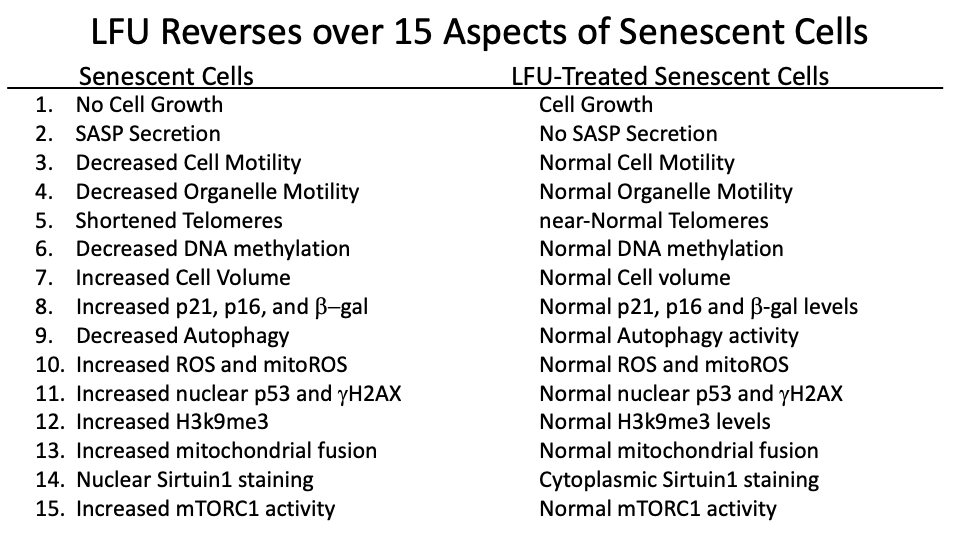
**

Fig. S12 Summary figure.


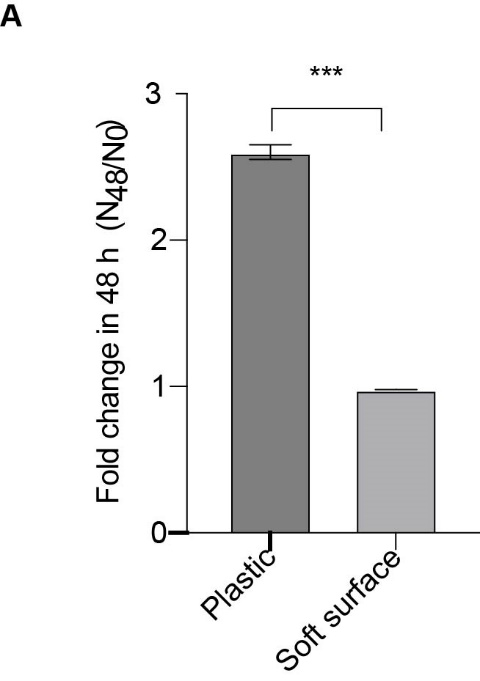


**Fig. S13 Rigidity Dependence of Rejuvenated Cell Growth.** Rejuvenated cells grow on stiff substrate whereas their growth ceased on soft surface. Results are shown as mean ± SD. *** p value <0.0001.

**Table S1:** List of Genes upregulated in LFU-treated late-passage HFFs.

| **Bio Process** | **p-value** | **q-value** | **overlap_genes** |
| --- | --- | --- | --- |
| Influenza A | 1.619929e-08 | 9.881566e-07 | [IFIH1, RSAD2, OAS1, OAS2, MX2, OAS3, CCL5, MX1] |
| Measles | 7.942201e-08 | 2.422371e-06 | [IFIH1, OAS1, OAS2, MX2, OAS3, MX1, HSPA6] |
| Hepatitis C | 1.828574e-07 | 3.718100e-06 | [RSAD2, OAS1, OAS2, MX2, OAS3, MX1, IFIT1] |
| Coronavirus disease | 2.527530e-06 | 3.854483e-05 | [IFIH1, OAS1, OAS2, MX2, OAS3, MX1, ISG15] |
| NOD-like receptor signaling pathway | 1.344502e-03 | 1.640292e-02 | [OAS1, OAS2, OAS3, CCL5] |
| Herpes simplex virus 1 infection | 1.970480e-03 | 1.750871e-02 | [BST2, IFIH1, OAS1, OAS2, OAS3, CCL5] |
| Epstein-Barr virus infection | 2.009196e-03 | 1.750871e-02 | [OAS1, OAS2, OAS3, ISG15] |
| Human papillomavirus infection | 1.141362e-02 | 8.702885e-02 | [MX2, MX1, ISG15, OASL] |
| RIG-I-like receptor signaling pathway | 1.483205e-02 | 1.005283e-01 | [IFIH1, ISG15] |
| Lipid and atherosclerosis | 1.935232e-02 | 1.180492e-01 | [CCL5, HSPA6, CD36] |

**Table S2:** List of Genes downregulated in LFU-treated late-passage HFFs.

| **Bio Process** | **p-value** | **q-value** | **overlap_genes** |
| --- | --- | --- | --- |
| GnRH secretion | 0.001055 | 0.119259 | [KCNJ6, SPP1, KCNN2, CACNA1H] |
| Calcium signaling pathway | 0.006934 | 0.309739 | [CHRM2, FGF7, HTR2B, BDKRB1, TACR1, CACNA1H] |
| Serotonergic synapse | 0.008223 | 0.309739 | [GABRB3, KCNJ6, HTR2B, KCNN2] |
| Regulation of actin cytoskeleton | 0.018747 | 0.394402 | [CHRM2, ACTR3C, FGF7, SCIN, BDKRB1] |
| GABAergic synapse | 0.024628 | 0.394402 | [GABRB3, KCNJ6, SLC38A5] |
| Morphine addiction | 0.026083 | 0.394402 | [GABRB3, KCNJ6, PDE3B] |
| Circadian entrainment | 0.030721 | 0.394402 | [KCNJ6, PRKG2, CACNA1H] |
| Neuroactive ligand-receptor interaction | 0.033034 | 0.394402 | [CHRM2, GABRB3, GRID1, HTR2B, BDKRB1, TACR1] |
| Pathways in cancer | 0.033440 | 0.394402 | [ARNT2, FGF7, BMP2, LAMC3, IGF2, BDKRB1, WNT2, GSTM5] |
| Tryptophan metabolism | 0.034903 | 0.394402 | [KYNU, CYP1B1] |
