# Supplementary material for: Rejuvenation of Senescent Cells, In Vitro and In Vivo, by Low‐Frequency Ultrasound
Source: Aging Cell. 2025 Mar 3;24(6):e70008. doi: 10.1111/acel.70008 (PMC12151899; doi:10.1111/acel.70008)
Supplement: Supplementary file 2 — Data S2. [file ACEL-24-e70008-s002.docx]

Materials and methods:

**Cell lines and cell culture**

Human Foreskin Fibroblasts (HFFs) and Bone marrow-derived mesenchymal stem cells (MSCs) were purchased from the ATCC. African monkey kidney-derived Vero cells were obtained from the M. Garcia-Blanco lab as a gift. All these cell lines were cultured as per manufacturer’s protocol. Vero cells and HFFs were in growth medium containing Dulbecco’s Modified Eagle’s Medium (DMEM) 10% fetal bovine serum (FBS; Gibco) and 1% Penicillin/ Streptomycin. Human MSCs were cultured in MSCs-approved medium (ATCC) and expanded as per the supplier’s protocol. Culture medium was changed every 48 h unless otherwise stated. Cells plated at 20-40% confluency were maintained in an incubator at 37^0^C and 5% CO_2_. Cells were passaged every 48-72 h using Trypsin/EDTA (Gibco). Cells were counted manually using a hemocytometer and ImageJ in at least three independent replicates unless otherwise stated. Minimally 5 fields were counted from each replicate.

**Antibodies for immunofluorescence**

Rabbit polyclonal p21 antibody (cell signaling) Cat#12D1, Mouse p21 antibody Santa Cruz #SC6246, Mouse monoclonal p16 antibody (abcam) Cat#ab211542, Mouse anti-5mc abcam #ab10805, Rat anti-BrdU abcam #ab6326, Mouse anti SIRT1 monoclonal #S5196, Donkey anti-rabbit #A21206, Goat anti-mouse #A32727, Alexa Fluor 594 rabbit anti-mouse #A11062, Alexa Fluor 488 Goat anti-rat #112-545-175, and Alexa Fluor 488 Donkey anti-Goat #ab150129.

### **MSC differentiation assays**

LFU-treated and untreated P18 MSCs were cultured in a 12-well culture plate in growth medium for 24 h. Then, growth medium was replaced by adipogenic (Invitrogen) or osteogenic (Invitrogen) differentiation media as per the manufacturer's protocol. Adipocytes were assayed after 12 days by Oil Red O (Sigma Aldrich) staining and osteocytes were assayed using an Alizarin red S dye (Sigma Aldrich) solution. Images were acquired using a 10x evos RGB objective.

**Senescence induction and quantification**

Vero cells were treated with various stressors, including 200 μM H_2_O_2_, 4 mM of sodium butyrate (SB), 25 μM bleomycin sulphate (BS) or 200 nM doxorubicin and incubated for 36-48 h. After washing with PBS and then adding fresh medium, cells were incubated for 4 days to confirm the growth arrest of senescent cells.^56^ HFFs were serially passaged until P15 as replication of these cells was dramatically reduced by P15-17. We used four criteria to determine if cells were senescent; (1) Cell cycle arrest by determining the growth rate, (2) Increase in cell spread area, (3) Development of a senescence-associated secretory phonotype (SASP) in culture medium and (4) β-galactosidase staining. We captured images of cells with an Evos microscope at 10X magnification after LFU treatment and 48 h post treatment. To measure growth by the increase in cell number, 15 random images were captured, then the average number of cells were determined, which was divided by the area of one frame to get the cell density (cells/cm^2^). Then this seeding density was multiplied by the total area of the dish or well to obtain the total number of cells after LFU treatment and after 48 h of incubation. The total number of cells at 48 h was divided by the total number of cells just after LFU treatment to determine the growth rate. If the ratio was one, there was no growth.

Senescence was detected by the β-galactosidase senescence staining kit as per the manufacturer’s protocol. Briefly, sub-confluent senescent cells were stained by the SA-β-galactosidase staining solution and incubated overnight at 37^0^C. The β-galactosidase-stained cells appeared blue and were considered senescent cells. The percentage of β-galactosidase-positive cells was determined by counting the number of blue cells and dividing by the total number of cells. Cell spread area was determined by capturing the images of cells with a 10X objective using an Evos microscope. Then, we used ImageJ software to calculate spread area by manually encircling the cell periphery of each cell. We used a minimum of 150 cells for the analysis. To determine the SASP activity, we cultured the senescent cells for 3-4 days and then supernatant was collected from each dish. This supernatant was used to culture normal cells. Development of a senescence phenotype by the normal cells in the supernatant medium confirmed that senescent cells were secreting SASP.

**LFU treatment of cells**

Prior to LFU treatment, the plates containing senescent Vero cells or late nssage HFF cells were wrapped with parafilm to avoid contamination and water influx into the plate. The samples were placed on the plastic mesh, which was mounted on the water tank with an ultrasound transducer. Water in the tank was degassed and heated to 35^0^C. The distance between the sample and transducer was approximately 9-10 cm. We also ensured that there were no air-bubbles or air-water interfaces between the water and the sample. Output power of the transducer was measured at the plate location by a calibrated needle hydrophone (ONDA MCT-2000). Cells were treated with pressure pulses of intermediate power and low frequency for 30 mins. Cells were treated with a 50% on-off duty cycle. After LFU treatment, cell plates were returned to the incubator for 48 h to determine the growth of senescent cells.

**Reversal of senescence**

Firstly, we induced senescence in Vero cells using sodium butyrate, then we confirmed senescence using growth arrest and β-galactosidase staining after four days of incubation which normally eliminated quiescent cells *^56^*. The senescent cells were treated by LFU with optimized parameters (power, frequency, and duty cycle) and incubated for 48 h to measure the growth and morphology of the cells before trypsinization (passage P0). These cells were then trypsinized, reseeded and incubated for 48 h for the P1 passage. This process was repeated to a P3 passage. Growth in number, morphology, β-galactosidase and EdU incorporation were measured and all indicated that LFU reversed the senescence. Control cells without LFU treatment remained senescent. Typically, by P3, the senescent cells exhibited the phenotype of normal proliferating cells. Passage 15-24 HFFs were treated with LFU at the optimized frequency and power. Cell proliferation was determined by counting the number of cells at the time of seeding and 48 h post LFU treatment. LFU-treated HFFs showed a higher growth rate than the untreated HFF cells. In the case of control P24 HFFs, they were treated with LFU and incubated for 96 h prior to trypsinization, reseeding and incubation for 48 h. Proliferation and morphology were measured after 48 h of incubation. P24, LFU-treated HFF cells became smaller in size, and they also showed dramatically greater proliferation than the untreated P24 HFFs.

**Senescence assay**

Senescence was detected by the senescence-associated b-galactosidase staining (Sigma Aldrich) as per manufacturer’s protocol. Briefly, senescent, non-senescent and LFU-treated cells were seeded in 27 mm ibidi glass bottom dish and incubated for 48 h. Then, these cells were fixed and stained per the manufacturer’s protocol. After adding staining solution into the dish, cells were incubated overnight at 37°C in absence of CO_2_. A minimum of 100 cells were counted manually for each condition of the analysis. 10–15 random images were captured for each condition for analysis of b-galactosidase-positive cells, which were counted manually.

**Mitochondrial morphology**

Ultrasound-treated cells were incubated with Mitotracker (Invitrogen) at 250 nM at 37^o^C for 30 min. Then, images were captured in by a confocal microscope for quantification (15 randomized fields per sample). Mitochondrial velocity was determined from the time lapse image by an Olympus microscope at 60X. Images were captured after every 5 seconds for 10 minutes. Mitochondrial mobility was determined by ImageJ software with manual tracking. 10-15 mitochondria were traced manually.

**Immunofluorescence staining**

Cells were fixed with 4% paraformaldehyde for 15 minutes, permeabilized with 0.5% Triton-X for 5 min and blocked with 3% Bovine serum albumin (BSA) for 1 h at room temperature and then incubated in primary antibody overnight at 4^0^C. The following primary antibodies were used: rabbit polyclonal p21 antibody (cell signaling) (1:400), Mouse monoclonal p16 antibody (1:300) (Abcam), Mouse H3k9me3 (1:500) (ThermoFischer), Mouse p53 (1:200:) (SantaCruz Bio), Rabbit Gamma H2XA (1:200:) (Cell Signaling), Mouse monoclonal SIRT1 antibody (1:300:) (Sigma), p-mTOR, Ser 2448 (Cell-signaling D9C2) (1:200:), Rabbit Anti-YAP (ab52771) (1:300:), α-Tubulin (ab7291) (1:500:). After washing with PBS, cells were incubated with goat anti-mouse 488 (1: 1000) (Sigma) and Donkey anti-rabbit 555 (1: 1000) (Invitrogen) for 2 h. Nuclei were stained with Hoechst at a 1: 5000 dilution. Images were acquired by spinning-disk confocal microscope. Mitotracker green (Invitrogen) at 100 nM, Lysosome tracker deep red (Invitrogen) at 50 nM, and Tubulin tracker deep red 1: 1000: were used as fluorescent tags. Sodium butyrate-treated HFF cells were incubated in MitoSOX Red (Invitrogen) and ROS probe (Dojindo’s ROS Assay Kit) as per manufacturer’s protocol. Briefly, cells were treated with 5 mM of MitoSOX Red and 1:1000 ROS detection dye in HBSS for 30 mins. Then, cells were washed twice with HBSS and treated with LFU and Cells were imaged at Olympus microscope using Ex/EM 490/520 and EX/EM 396/610 in controlled environment at 5% CO_2_ and 37^0^C.

**Cytokine profiling by multiplex cytokine assay**

To analyze the level of chemokines, cytokines and immunoregulatory proteins in the supernatant of LFU-treated late passage and P3 HFFs, we used Bio flex cytokines 27-flex kit assay (Bio-Rad). This multiplex immunoassay assay contained fluorescence nanoparticles conjugated with a specific antibody and was used according to the manufacturer’s protocol. Briefly, supernatant collected from the late passage HFFs and P3 cells treated with or without LFU, were diluted 1:4 and incubated for 2 h at room temperature in shaking. Then, wells containing samples were washed, and incubated with biotinylated antibody for 1 h at room temperature, then with streptavidin-phycoerythrin for another 30 min. A calibration curve was established using the standard given with the assay kit. Bioplex-200 plate reader (UTMB Galveston, Texas core facility) was used to assess the cytokine level. The concentrations of molecules were determined using the standard curve provided by the manufacturer.

**Analysis of autophagic flux**

To analyze LFU-induced autophagy in late passage HFFs and in LFU treated HFFs, we used Prema autophagy Tandem sensor RFP-GFP-LC3 kit (Invitrogen). Tandem sensor has acid sensitive GFP and acid insensitive RFP. In active autophagy, fusion of lysosomes with autophagosomes increases acidity, which quenches the GFP. Cells were cultured in 35 mm glass bottom dishes for 24 h before LFU treatment. Cells were transduced with autophagy tandem sensor according to the manufacturer’s instructions. Briefly, 40 particles per cells were used for transduction, and incubated overnight in culture medium. Chloroquine diphosphate (100 μM) was used as an autophagy blocker overnight. Cells were visualized and images were captured at 100x on a spinning-disk confocal microscope (Olympus). Autophagy was determined as the ratio of intensities of GFP and RFP.

**Telomere length measurement**

The length of telomere was determined using Absolute Human Telomere Length Quantification qPCR Assay Kit (ScienCell AHTQL-8918) according to the manufacturer’s protocol. Total genomic DNA was extracted from Human foreskin fibroblasts of various conditions including early passage (P2), late passage (P18) cells, LFU-treated and untreated control cells using Invitrogen Pure Link Genomic DNA kit (K182001). 5 ng of genomic DNA was used as a template in PCR. Every experimental genomic DNA was processed four times. The reference DNA was analyzed in two triplicates. Telomere length was calculated according to the manufacturer’s protocol.

**DNA methylation assay**

The protocol for DNA methylation was performed as reported previously.^82^ Cells were incubated in 20 µM CldU for 16 h. After trypsinization, cells were harvested and resuspended in ice-cold PBS. A 2 µl droplet containing 200-300 cells was diluted in 8 µl of Lysis buffer (0.5% SDS, 50 mM EDTA, 200 mM Tris-HCl, pH 7.4). A 10 µl droplet containing cells diluted in Lysis buffer was poured onto the silane prepared slide (Sigma Aldrich), tilted, and allowed to flow by gravity. The slide was then fixed in methanol/ acetic acid (3:1) for 25-30 min and rinsed with PBS thrice. Slides were then denatured in 2.5 M HCl for 1 h; neutralized in 0.4 M Tris-HCl for 5 min and blocked in 5% BSA buffer for 1 h. Slides were stained with anti-BrdU (1:200) and 5mc (1:200) antibodies overnight at 4^O^C. Slides were stained with Alexa flour 488 goat anti-rat (1:1000) and Q-dot 655 goat anti-mouse (1:2000) for 1 h for BrdU and 5mc antibody respectively.

**RNA extraction and sequencing**

RNA was extracted from early passage HFFs (P2, P3, and P4) and rejuvenated senescent cells (P18, P19, and P20) using Qiagen RNeasy Mini Kit. (Germantown, MD). The UTMB Next Generation Sequence (NGS) core laboratory assessed RNA concentrations and quality using a Nanodrop ND-1000 spectrophotometer (Thermofisher, Waltham) and an Agilent Bioanalyzer 2100 (Agilent Technologies, Santa Clara, CA). PolyA+ RNA was purified from ~100 ng of total RNA and sequencing libraries were prepared with the NEBNext Ultra II RNA library kit (New England Biolabs) following the manufacturer’s protocol. Libraries were pooled and sequenced on an Illumina NextSeq 550 High Output flow-cell with a single-end 75 base protocol. Reads were mapped to the human GRCh38 reference genome with STAR version 2.7.10a with the parameters recommended for the ENCODE consortium. Reads mapping to genes were quantified with the STAR –quantMode.^83^ GeneCounts option using the Gencode v41^84^ primary assembly annotation file. Differential gene expression was estimated with the DESeq2 software package, version 1.38.3, following the vignette provided with the package.^83^ The complete RNA-seq data will be posted online.

**Ca^2+^ influx measurement via live imaging**

Ca^2+^ influx was measured via live Ca^2+^ imaging using replicative mesenchymal stem cells. Briefly, cells were seeded on fibronectin coated glass bottom 35mm dishes overnight. Cells were then incubated with Ca^2+^ indicator Calbryte 520 AM for 30 minutes. After 30 minutes, staining media was replaced with live-cell imaging media with no Ca^2+^. Live imaging was done at 10X magnification using 488 laser line. Each imaging session was obtained for 10 minutes with 2 seconds of interval. LFU or drug treatment was administered after about 2 minutes of the starting of each session. Data was analyzed by obtaining multiple ROIs using ImageJ software. These values were then subtracted by the background fluorescence and then normalized by the minimum values to obtain F/F_0_. These F/F_0_ values were then used to plot Ca^2+^ curves using GraphPad Prism.

**Soft surface preparation:**

PDMS of 2-5 kPa elastic modulus was prepared by mixing the Sylgard 184 silicone elastomer kit at an Elastomer/curing ratio in 80:1. The combination was mixed, degassed and spin coated at 4000 rpm for 20 seconds on 27 mm ibidi glass bottom dishes. PDMS coated dishes were incubated at 65°C overnight. Dishes were cleaned, activated by oxygen plasma treatment, then coated with 25 μg/ml Fibronectin (Sigma Aldrich) and kept overnight at 4°C. Coated dishes were washed with PBS before plating the cells

**Animals**

All mice used in the study were purchased from Jackson’s lab (JAX 000664 and C57BL/6J strain) and maintained in the Animal research center (ARC) at UTMB. All mice were received regular chow diet. All animal-related procedures including housing, euthanasia, non-survival surgery, tissue collections and experimental procedures were approved by the Institutional Animal Care and Use Committee (IACUC) in the protocol number 2102013, at UTMB Galveston. Each experimental group was comprised of 22-25 month old mice with males and females. Equal numbers of mice were used in each group unless otherwise stated.

**LFU treatment of aged mice**

Aged mice (21-24 months old) were treated in a 4L glass beaker with an internal plastic cylinder of 13 cm height and 15.2 cm in diameter. A plastic mesh was placed on top the cylinder that supported the mice and enabled them to rest with their four limbs and body in the water. Degassed, 32-35°C water was poured into the beaker to a level of 1 inch above the plastic mesh so that half of the bodies of the mice were in water. Once the mice were placed in the water, intermittent ultrasound of low frequency and intermediate power was applied to the mice. The reason for putting animals in water was that ultrasound was attenuated dramatically at air-water interfaces. Animals in the ultrasound groups were treated at 72 h intervals for one month (10 treatments). During the ultrasound treatment, we carefully observed mouse activity and their adaptation to the system. After treatment, the animals were placed in a separate cage with tissue paper to dry the animals and then they were returned to their home cage. Control mice were placed in the same water bath for 30’ without ultrasonication.

**Physical assessment of the mice**

For assessment of the effect of LFU treatment on physical performance of the mice, we used 6 groups of old mice, 1. Sham, 2. LFU treatment, 3. Exercise treatment, 4. Rapamycin, 5. Exercise plus LFU, and 6. LFU plus Rapamycin. Each group contained four males and four females. In the case of the rapamycin-treated animals, the C57BL/6J mice were fed with encapsulated rapamycin and monitored daily for a month. Animals of LFU groups were treated every 72-96 h for a month. Animals in the exercise group were trained three times per week on a treadmill for 25’ in each exercise training session. Prior to starting the experiment, we assessed the physical functions and health condition of the mice, identified as pre-assessment. After one month of LFU treatment and/or exercise sessions, we again assessed the physical performance and health conditions of the animals, identified as post-assessment. Physical performance was determined by the functional assessment tests including Grip test, Rotarod, Treadmill, and Inverted Cling tests (33).

a) Inverted Cling: This grip test was useful to quantify muscle strength and endurance by measuring how long the mouse held onto the grid while inverted. Each animal was tested 2 times with a resting break of 10 min between trials. A minimum holding time of 10 seconds was required for test validation to exclude a slip. Three trials were conducted with a gap of 10 mins between trials.

b) Treadmill: The mice were tested for the maximum power output/maximum gait speed and endurance (to exhaustion) by running on a treadmill. The measurement was the duration of the running. During the training session, the mice were familiarized with the device, first running at a constant speed, later increasing the speed progressively one unit in every 20 seconds. The mice were allowed to rest 10 min between trials. Three electric shocks of 0.4 mA ended the trial and animals were given three trials. During each trial, the speed was increased one unit in every 20 seconds, and each mouse was allowed to run as long as they could before getting three shocks.

c) Activity Wheel: Mice treated with 4 kPa, 5.2 kPa, and 8 kPa at every day (D1), 4 kPa every 48 h (D2) and 4 kPa every 72 h (D3). Each group had 6-8 mice per group. To measure activity, mice were housed singularly in wheel cages (Columbus Instruments, USA). All mice were kept for 72 h to determine the activity of each mouse. After 72 h, data were acquired in terms of total number of turns in the same period.

d) Exercise training sessions: Animals of Exercise and Exercise + LFU groups were exercised on a treadmill for 25 min at three times in a week for one month. Every exercise session was preceded by 10 min warm up at 6 cm/s, followed by 10 min training at 8 cm/s, and 5 min cool down at 6 cm/s. Training was progressive starting at 8 cm/s in the first week and increasing to 11 cm/s in week 4. The mice were encouraged to run on treadmill with a light electric shock when they stopped running. There were 12 exercise training sessions and 10 LFU treatments in the one month experimental period.

**Lifespan analysis**

Mice were regularly treated as per the experiment design and their health was monitored. Sham mice were placed in the LFU treatment setup in OFF condition for 30’ and the mice housed in the animal facility without any treatments were our negative control. Survival curve was determined based on the number of mice alive at various time points.

**Immunohistochemistry**

Kidney and pancreas were collected after completion of post physical assessment under deep anesthesia. Both the organs were flash frozen in isopentane -liquid nitrogen. Organs were sectioned in a -20°C cryostat and stored at -20°C. Kidney and pancreas sections of 6-8 um thickness were fixed and stained with SA β-galactosidase as described in the manufacturer’s protocol. Sectioned samples were incubated at 37°C in a CO2-free incubator. Sections were stained with hematoxylin and eosin y for cytoplasmic and nuclear staining per standard protocol. Images were captured at 20X in the RGB channel of EVOS microscope. The fraction of β-galactosidase stained tissue was determined using ImageJ.

**Immunofluorescent detection of p21 and p16 in Kidney and Pancreas**

Pancreas and Kidney sections were immunoassayed by treating with 4 % paraformaldehyde for 15 minutes, permeabilizing with 0.5% Triton X-100 in PBS for 2 min at 25°C, blocking with 10% Bovine serum albumin for 1 hour at 25°C, and incubating with rabbit anti-p21 antibody (Abcam, ab188224) I and mouse p16 antibody (Cell signaling) overnight at 4°C. Fluorescent secondary antibodies [Invitrogen, goat anti- mouse 488 (800:1) and donkey anti-rabbit 555 (800:1) were added, and the samples were incubated at 4°C for 4 hr in a humidified chamber followed by washing with PBS. Images were acquired with an Olympus light microscope using 60x oil objective. Images of were captured in Evos microscope at 20x. A minimum of five unique fields of view were analyzed per sample of pancreatic and kidney tissue obtained from four mice per group.

**Statistical analysis**

All experiments data reported were obtained from the minimum of three samples or pre group otherwise mentioned in the figure legends. Data is represented as the mean ± standard deviation and Statistical analysis was performed using GraphPad prism 10.0. Differences in the two group is determined using the two-tailed paired, unpaired Student’s *t*-test, Mann Whitney tests, and Kruskal Wallis, and One way ANOVA test followed by post hoc Dunn's test used for the multiple groups. Statistical significance was analyzed and reported in the figures and figure legends *, *P* values < 0.05, **, *P* values < 0.002, ***, *P* values < 0.001, ****, *P* values < 0.0001 and non-significant (ns) *P*-value > 0.05.
